# Unimolecular Exciplexes by Ugi Four-Component Reaction

**DOI:** 10.3389/fchem.2019.00717

**Published:** 2019-11-01

**Authors:** Maria Ochs, Bernhard Mayer, Thomas J. J. Müller

**Affiliations:** ^1^Institut für Organische Chemie and Makromolekulare Chemie, Heinrich-Heine-Universität Düsseldorf, Düsseldorf, Germany; ^2^Ernst-Berl Institut für Technische and Makromolekulare Chemie, Technische Universität Darmstadt, Darmstadt, Germany

**Keywords:** absorption, bichromophores, DFT, emission, energy transfer (ET) dyes, exciplexes, isonitrile, multicomponent reaction

## Abstract

Exciplex or excited complex emission is an excited state process, arising from considerable charge transfer of an excited energy donor to an acceptor, which can be identified by the occurrence of a redshifted emission band that is absent in the individual constituents. Particularly interesting are exciplexes that are formed by intramolecular excited state interaction, which are inherently concentration independent. Based upon our previous experience in the Ugi-4CR syntheses of donor-acceptor conjugates capable of photo-induced intramolecular electron transfer (PIET), that is, generation of light-induced charge separation, we now disclose the diversity-oriented approach on unimolecular exciplex emitters and their reference systems by Ugi-4CR. The photophysics is studied by absorption and emission spectroscopy and accompanied by density functional theory (DFT) and time-dependent density functional theory (TDDFT) calculations.

## Introduction

**Figure 1:**
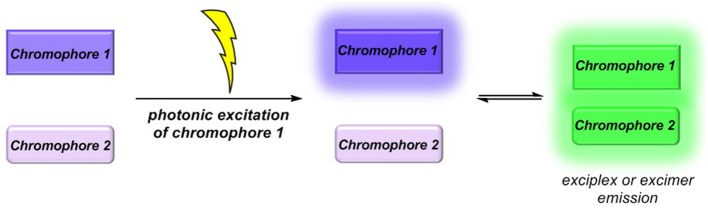
Exciplex or excimer emission as a radiative quenching pathway by intermolecular chromophore-chromophore interaction in the excited state.

Molecular luminescence (Lakowicz, 2006; Valeur and Berberan-Santos, 2012) is a widespread phenomenon of functional organic materials (Müller and Bunz, 2007) and finds broad application in many fields of science and technology, ranging from fundamental science (luminescence spectroscopy) (Wolfbeis, 1993; Valeur and Brochon, 2012) over biophysical analytics (Chen et al., 1998; Nilsson et al., 2002; Wagenknecht, 2008; Demchenko et al., 2009; Kim and Park, 2009; Cairo et al., 2010), diagnostics (Kobayashi et al., 2010; Carter et al., 2014), and dye lasers (Thiel, 2000; Shankarling and Jarag, 2010) to sensors (Loving et al., 2010; Klymchenko, 2017; Zhang et al., 2017) and organic light-emitting diodes (OLED) (Müllen and Scherf, 2006; Park et al., 2011; Thejo Kalayani and Dhoble, 2012; Li, 2015). Besides fluorescence and phosphorescence as radiative deactivation of electronically excited singlet or triplet states, also radiative states arising from inter- or intramolecular electronic interaction, eventually by energy or electron transfer, are particularly interesting. With this respect, exciplexes (excited complexes) and excimers (excited dimers) are emissive charge-transfer complexes, which are formed by excitation of one of the constituting chromophores that in turn collides with a second chromophore in its electronic ground state (Balzani et al., 2003; Balzani, 2008). In contrast to charge-separated charge transfer states, exciplexes and excimers can deactivate by broad structureless emission (Lakowicz, 2006; Valeur and Berberan-Santos, 2012), however, redshifted with respect to the constituting individual chromophores (Figure 1) (Balzani et al., 2003; Balzani, 2008). While excimers constitute from identical chromophores in exciplexes, the two colliding chromophores differ in their structure and nature. In either case, stability results from Coulomb and favorable orbital interactions at distances between 3 and 4 Å. Since exciplex emission represents a pathway of quenching the excited molecule's fluorescence, this phenomenon might be employed for sensing proximity of two interacting chromophores by a radiative process. This excited state phenomenon is particularly interesting—absorption spectra are simple superpositions of their constituting chromophores—as the emission will be dependent on the solvent polarity in terms of energy of the exciplex state as well as in terms of the intensity (Kavarnos, 1993).

If two chromophores are placed into a unimolecular setting, that is, by covalent ligation, the unimolecular exciplex emission is inherently independent of concentration and diffusion control, in contrast to exciplex emission arising from two particles. The positive solvatochromicity of the exciplex emission band, that is, a redshift of the longest wavelength emission band with increasing solvent polarity, as well as its decreasing effect on the fluorescence quantum yield and fluorescence lifetimes has impressively been shown for ether-tethered dyads consisting of pyrene and 2-benzylidenemalononitrile as constituting chromophores (Piuzzi, 1993; Zhang et al., 1997; Piuzzi et al., 1999). Likewise, alkyl-tethered pyrene/anthracene-*N,N*-dimethylaniline dyads were studied in detail by time-resolved laser spectroscopy (Mataga and Miyasaka, 1999). In hexane, the formation of the compact exciplex, where the hydrocarbon and the *N,N*-dimethylaniline moieties are sandwiched, occurs in the nanosecond time regime, while in acetonitrile, the loose exciplex forms within picoseconds after excitation, obviously due to a facilitated charge-transfer proceeding without conformational changes. Time-resolved transient absorption spectroscopy moreover supports that the compact exciplex bands are strongly broadened in contrast to the acetonitrile spectra, which clearly support the well-resolved signature of superimposing radical ion spectra, that is, a strong ion pair character. Intermediate solvent polarities account for signal broadening, that is, the population of more ordered compacted conformations.

As part of our program to methodologically develop concise syntheses of functional chromophores by multicomponent reactions (MCR) (Levi and Müller, 2016a), we are particularly interested in MCR syntheses of fluorophores (Levi and Müller, 2016b; Riva et al., 2016; Merkt and Müller, 2018; Müller, 2018) and donor-acceptor systems that interact in the excited state *via* photo-induced electron transfer (PIET) (Kavarnos, 1993; Lemmetyinen et al., 2011; Wenger, 2011; Ricks et al., 2012; Vauthey, 2012), as shown by non-radiative fluorescence quenching (Bucci and Müller, 2006; Bay et al., 2013, 2014; Bay and Müller, 2014). This can be considered as chromophore-chromophore interactions at short distances, that is, between Dexter and Förster radii.

**Figure 2:**
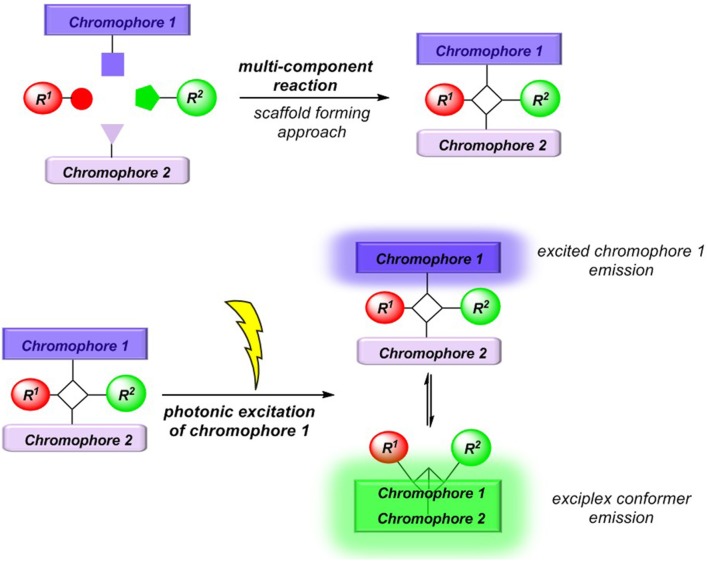
MCR synthesis (scaffold-forming approach) of exciplex forming bichromophores.

Conceptually, we reasoned that employing a scaffold-forming MCR approach (Levi and Müller, 2016a) to exciplex-forming bichromophores could establish a concise general synthetic approach to these types of unimolecular exciplex emitters (Figure 2). In addition, if emission of non-interacting and exciplex conformers occurs simultaneously from this Boltzmann distribution, dual emission may arise, furnishing emission color mixing. Here, we report the Ugi-4CR synthesis of several representative unimolecular exciplex emitters consisting of an *N,N*-dimethyl aniline moiety as a donor and anthracene, naphthalene, or pyrene as acceptor chromophores, as well as reference chromophores. Furthermore, the absorption and emission spectroscopic characteristics are reported and discussed in the light of DFT and TDDFT calculations of the donor-acceptor systems.

## Results and Discussion

### Synthesis

The Ugi four-component reaction (4CR) (Dömling and Ugi, 2000; Dömling, 2006; Biggs-Houck et al., 2010) generates the chemically robust α-aminoacyl amide scaffold with a high level of diversity and has widely and successfully been applied in biological and medicinal chemistry (Dömling et al., 2012). Encouraged by Ugi 4CR syntheses of a phenothiazine-anthraquinone-based PIET system and related dyads (Bay et al., 2013, 2014; Bay and Müller, 2014), characterized by cyclic voltammetry, steady-state UV/vis, and fluorescence spectroscopy, as well as femtosecond transient absorption spectroscopy for identification of the desired charge separated state after light excitation, we decided to transpose the Ugi 4CR to a novel synthesis of *N,N*-dimethylaniline-anthracene dyads, which are known to form exciplex emitters (Hui and Ware, 1976).

Methanol as a solvent is often most ideal for Ugi 4CR, however, portions of dichloromethane were found to beneficially increase the solubility and to ensure a homogeneous solution. In a model reaction employing 9-aminomethylanthracene hydrochloride (**1a**) (Stack et al., 2002), commercially available 4-(*N,N*-dimethylamino)benzaldehyde (**2a**), acetic acid (**3**), and *tert-*butylisocyanide (**4**), we set out to identify optimal conditions for the preparation of the targeted potential exciplex system **5** by variation of the base, reaction time, and conditions (see Table S1).

**Scheme 1:**
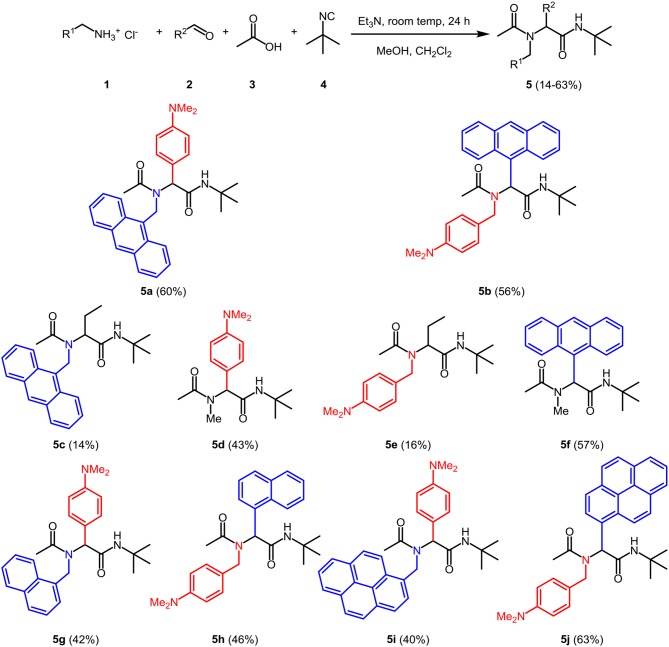
Ugi 4CR synthesis of donor-acceptor dyads and reference compounds **5**.

The optimal conditions at the equistoichiometric ratio (Table S1, entry 6) were therefore employed for the synthesis of other bichromophores and their reference systems **5** in this study, which were obtained in moderate to good yield (Scheme 1). For the donor-acceptor bichromophores, 4-*N,N*-dimethylamino phenyl derivatives were used as donor moieties, whereas the acceptor unit was altered from anthracene over pyrene to naphthalene. For solubility issues of pyrene derivatives in methanol, the amount of dichloromethane had to be increased.

The appearance of single-signal sets in the ^1^H and ^13^C NMR spectra of **5** unambiguously supports the structural assignment of the bichromophores, and rotamer mixtures due to restricted amide bond rotation can be excluded. The depicted structural assignments were made by identifying the lowest energy conformations by force field calculation scans of the corresponding structures **5**. Distinct resonances in the aromatic region of the spectra account for 9-anthracenyl, 3-pyrenyl, and 1-naphthyl and the *p*-*N,N*-dimethylamino phenyl substitutions, respectively. Mass spectrometry and combustion analysis are additionally in agreement with the structures of bichromophores **5a,b,j-h** and the corresponding reference compounds **5c-f**.

### Photophysical Properties

**Figure 3:**
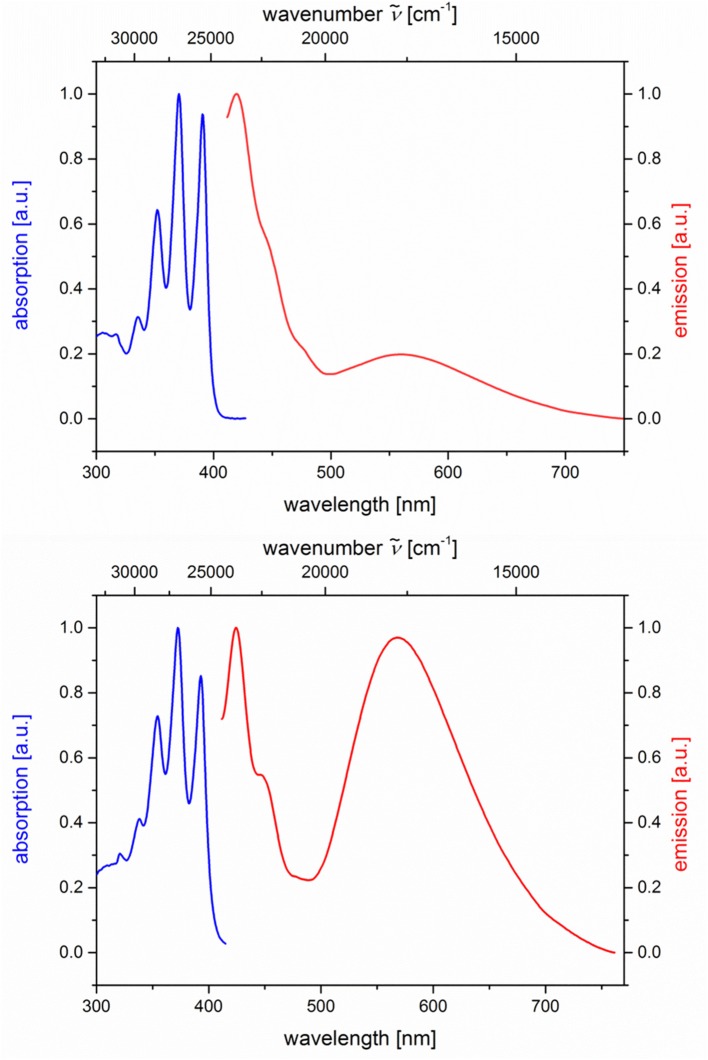
Normalized absorption (blue lines) and emission (red lines) spectra of compounds **5a (top)** and **5b (bottom)** (recorded in CH_2_Cl_2_ (chromasolv), *T* = 298 K, λ_*exc*_ = 391 nm).

The absorption and emission spectra of all donor-acceptor dyads and their reference compounds were measured in dichloromethane at room temperature (Table 1). By comparison of the UV/Vis spectra of *N,N*-dimethylaniline-anthracene dyads **5a** and **5b** with *N,N*-dimethylaniline (**5d** and **5e**) and anthracene (**5c** and **5f**) reference compounds, it can be clearly seen that in the electronic ground state, which is reflected by absorption spectroscopy, no interaction of donor and acceptor subchromophores can be detected by the absence of charge transfer bands. In addition, the spectra of the dyads **5a** and **5b** show an additive behavior with respect to the spectra of the reference compounds **5c** and **5d** and **5e** and **5f**, respectively (Table 1). However, in the excited state upon excitation at λ_*exc*_ = 391 nm, that is, the longest wavelength absorption band of the anthracene, in the emission spectra besides the anthracene characteristic vibrationally resolved fluorescence for both dyads, **5a** and **5b**, a broad unstructured and redshifted emission band at λ_*max,em*_ = 561 (dyad **5a**) and 568 nm (dyad **5b**) is detected (Figure 3). This redshifted band is most characteristic for the *N,N*-dimethylaniline-anthracene exciplex. With respect to the isomer correlation of the dyads **5a** and **5b** and the essentially identical energy of this transition, it can be concluded that the placement of the aniline and the anthracene can be introduced in either case *via* the aldehyde or the primary amine.

**Table 1:** Selected absorption and emission spectra of exciplex bichromophores (exciplex emission bands are highlighted in bold face) and reference compounds **5** (recorded in CH_2_Cl_2_ (chromasolv), *T* = 298 K).

**Compound**	**Absorption ***λ_max_*** [nm] (ε [Lmol^**−1**^ cm^**−1**^])**	**Emission ***λ_max,em_*** [nm]**
**5a**^**a**^	258 (112,100), 317 (1,600), 335 (2,100), 352 (5,100), 371 (8,300), 391 (7,700)	417, 444, 475, **561**
**5b**^**a**^	259 (128,600), 321 (2,300), 339 (3,200), 355 (5,600), 373 (7,800), 393 (6,600)	423, 448, **568**
**5c**^**a**^	259 (110,200), 336 (2,800), 352 (6,800), 370 (10,500), 391 (9,800)	418, 443, 471
**5d**^**b**^	268 (21,000), 302 (2,500)	353
**5e**^**b**^	264 (14,500), 307 (1,100)	356
**5f**^**a**^	258 (123,800), 337 (2,500), 353 (5,200), 371 (7,700), 391 (6,900)	420, 445, 475
**5g**^**b**^	270 (24,000), 283 (sh), 295 (sh), 309 (sh), 316 (sh), 353 (200)	337, **455**
**5h**^**b**^	265 (19,000), 284 (sh), 296 (sh), 312 (sh), 316 (sh), 365 (sh)	334, **472**
**5i**^**c**^	258 (sh), 268 (40,600), 278 (48,000), 300 (sh), 317 (13,500), 331 (27,000), 347 (36,000), 377 (800)	383, 397, **499**
**5j**^**c**^	259 (sh), 269 (35,600), 279 (43,400), 300 (sh), 317 (14,000), 331 (28,000), 348 (38,000), 377 (900)	380, 395, **538**

a *Excitation wavelength λ_exc_ = 391 nm*.

b *Excitation wavelength λ_exc_ = 268 nm*.

c *Excitation wavelength λ_exc_ = 348 nm*.

**Figure 4:**
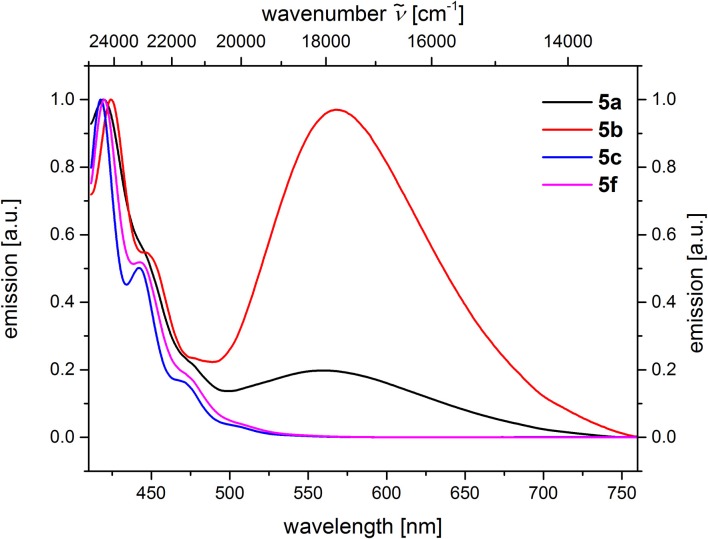
Comparison of emission spectra of compounds **5ac, f** (recorded in CH_2_Cl_2_ (chromasolv), *c*(**5**) = 10^−5^ m, *T* = 298 K, λ_*exc*_ = 391 nm).

**Figure 5:**
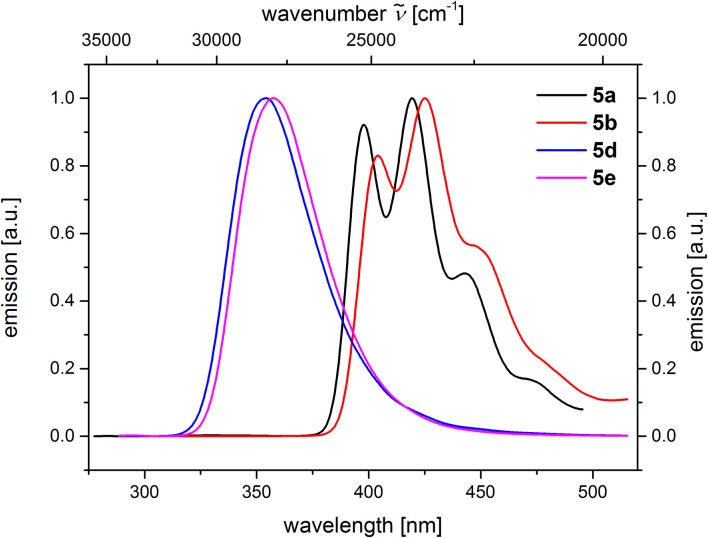
Comparison of emission spectra of compounds **5a** and **5b** (*c*(**5**) = 10^−5^ m, λ_*exc*_ = 268 nm), as well as compounds **5d** and **5e** (*c*(**5**) = 10^−4^ m, λ_*exc*_ = 258 nm) (recorded in CH_2_Cl_2_ (chromasolv), *T* = 298 K).

From the comparison of the emission spectra of dyads **5a** and **5b** and the anthracene reference compounds **5c** and **5f** at identical concentration and identical excitation wavelength [*c*(**5**) = 10^−5^ m, *T* = 298 K, λ_*exc*_ = 391 nm], it can be clearly seen that for both dyads, a radiative deactivation pathway of the anthracene luminescence by structureless broad exciplex emission bands exists (Figure 4). From the relative emission intensities of the exciplex bands and the residual anthracene emission, it can be seen that the exciplex band of dyad **5b** is more intense than that of dyad **5a**. Upon excitation of the dyads **5a** and **5b** at the main absorption bands of the *N,N*-dimethylaniline references **5d** and **5e** (λ_*exc*_ = 268 nm), the comparison of the emission spectra reveals that for the dyads, the anthracene emission arises from complete energy transfer from the excited *N,N*-dimethylaniline moieties on expense of the characteristic aniline emission, which can be identified in the emission spectra of the reference compounds **5d** and **5e** (Figure 5). Furthermore, the emission spectrum of dyad **5a** when excited at 268 nm reveals at this excitation wavelength that both the anthracene emission bands and the broad exciplex emission band are can be unambiguously detected (see Figure S24). The energy excitation of the exciplex emission arising from reductive electron transfer (from *N,N*-dimethylaniline to excited anthracene) clearly is apparent.

**Figure 6:**
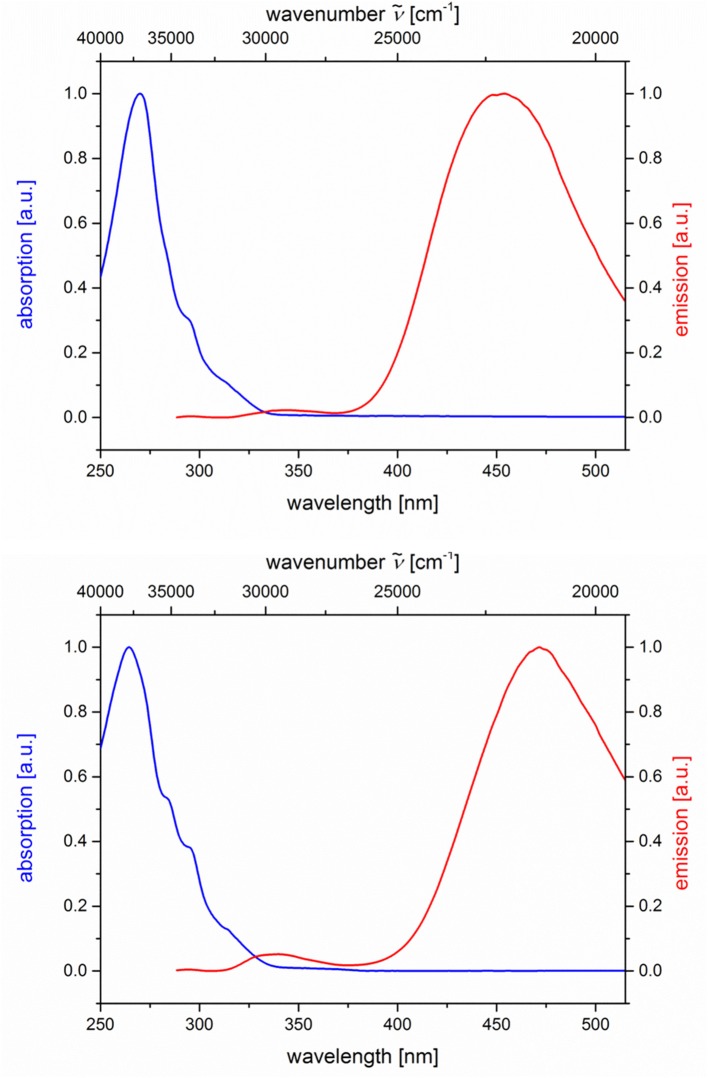
Normalized absorption (blue lines) and emission (red lines) spectra of compounds **5g (top)** and **5h (bottom)** (recorded in CH_2_Cl_2_ (chromasolv), *T* = 298 K, λ_*exc*_ = 268 nm).

The dyads **5g** and **5h** consist of naphthalene as an acceptor and *N,N*-dimethylaniline as a donor (Table 2; Figure 6). Upon excitation at λ_*exc*_ = 268 nm, the emission spectra reveal weak naphthalene emission bands that are quenched in favor of exciplex emission at 455 and 472 nm, respectively. These differences already indicate that the overlap of naphthalene and *N,N*-dimethylaniline in the sandwich conformer might differ in both isomers.

**Table 2:** Rehm-Weller estimation of the PIET's Gibbs free energies Δ*G*_*PIET*_ calculated from the energy differences of the redox potentials *E*_0_(Me_2_NPh^+^/Me_2_NPh) – *E*_0_(hydrocarbon/hydrocarbon ^−^), the excitation wavelengths *E*_00_, and the solvent correctional term ${\Delta G^{0}}_{solv}$ for *syn*-conformers of selected dyads **5**.

**Compound**	***E_**0**_*(Me_**2**_NPh ^**+**^/Me_**2**_NPh) – *E_**0**_*(hydrocarbon/hydrocarbon ^**−**^)^a^ [V]**	***E_**00**_*^b^ [eV]**	**${\Delta G^{0}}_{solv}$^c^***[eV]*	**Δ*G_**PIET**_* [eV]**
**5a**	2.74	3.17^a^	0.44	−0.87
**5b**	2.74	3.17^a^	0.39	−0.82
**5g**	3.10	4.63^b^	0.37	−1.90
**5h**	3.10	4.63^b^	0.33	−1.86
**5i**	2.85	3.56^c^	0.34	−1.05
**5j**	2.85	3.56^c^	0.34	−1.05

a *Determined from E_0_(Me_2_NPh ^+^/Me_2_NPh) = 0.81 V, and E_0_(anthracene/anthracene ^−^) = −1.93 V, E_0_(naphthalene/naphthalene ^−^) = −2.29 V, and E_0_(pyrene/pyrene ^−^) = −2.04 V, respectively*.

b *Calculated from excitation wavelength of **5a** and **5b** (λ_exc_ = 391 nm), **5g** and **5h** (λ_exc_ = 268 nm), and **5i** and **5j** (λ_exc_ = 348 nm)*.

c *Calculated for dichloromethane with a relative permittivity ε_r_ = 8.93. Donor-acceptor distances r_DA_ taken from MM2 calculations of the conformers with syn-orientation of donor and acceptor (r_DA_(**5a**) = 3.69 Å; r_DA_(**5b**) = 4.12 Å; r_DA_(**5g**) = 4.33 Å; r_DA_(**5h**) = 4.87 Å; r_DA_(**5i**) = 4.70 Å; r_DA_(**5j**) = 4.80 Å)*.

**Figure 7:**
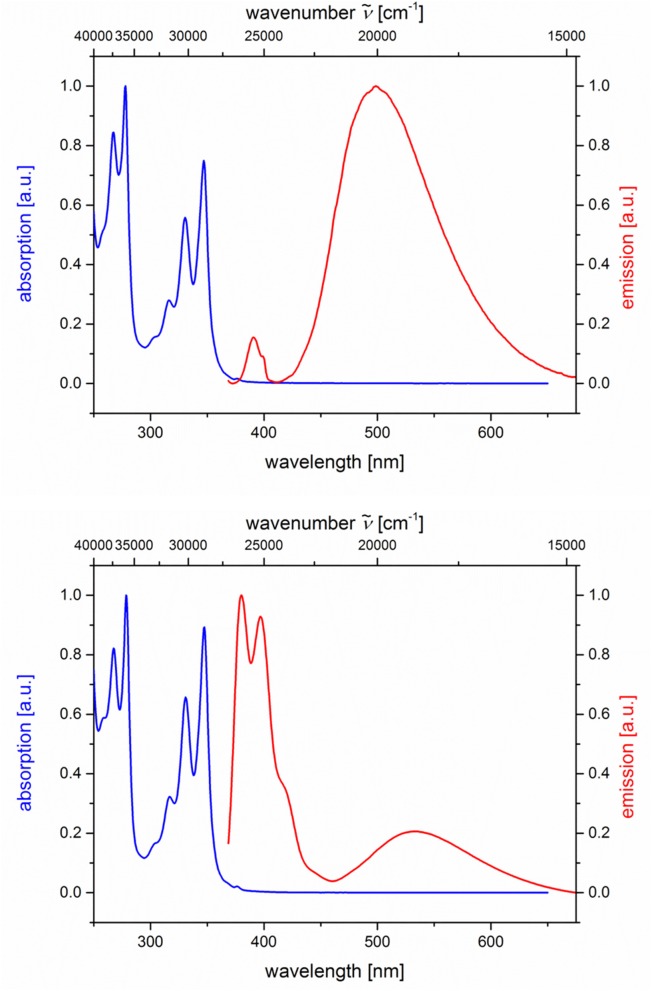
Normalized absorption (blue lines) and emission (red lines) spectra of compounds **5i (top)** and **5j (bottom)** (recorded in CH_2_Cl_2_ (chromasolv), *T* = 298 K, λ_*exc*_ = 348 nm).

Likewise and even more pronounced is the difference in exciplex emission of the pyrene-based dyads **5i** and **5j**, which, upon excitation at λ_*exc*_ = 348 nm, show well vibrationally resolved pyrene emission bands for isomer **5i** and a broad weaker exciplex band at 499 nm, whereas for isomer **5j** displays a broadened weaker pyrene emission and a more intense, broad exciplex band at 538 nm (Table 2; Figure 7).

For all three combinations of *N,N*-dimethylaniline-hydrocarbon dyads, exciplex formation occurs by photoexcitation of the hydrocarbon moieties and subsequent interaction with the ground state of the *N,N*-dimethylaniline moiety (Ceroni and Balzani, 2012). The exciplex formation depends on spatial proximity and as a partial electron transfer by the redox potentials of donor and acceptor moieties. The Rehm-Weller approximation (Rehm and Weller, 1970) allows an estimation of the PIET's Gibbs free enthalpy Δ*G*_*PIET*_ (Equation 1) by

(1) $$\begin{array}{r} \Delta G_{PIET}=E_{0}\left({\text{D}}^{+}/D\right)-E_{0}\left(\text{A}/{\text{A}}^{-}\right)-E_{00}-\Delta G_{solv}^{0}\left[\text{eV}\right], \end{array}$$

where *E*_0_(D^+^/D) is the donor's normal oxidation potential, *E*_0_(A/A^−^) is the acceptor's normal reduction potential (both are often determined from cyclic voltammetry and are given in V), *E*_00_ is the excitation energy of absorption band in eV (Nishizawa et al., 1998), and $\Delta G_{solv}^{0}$ (in eV) is a correctional term (Equation 2) describing the Coulomb stabilization of two point charges at a distance *r*_*DA*_ in a solvent according to

(2) $$\begin{array}{r} \Delta G_{solv}^{0}\;=\;\frac{e^{2}}{4\pi \varepsilon_{0}\varepsilon_{r}r_{DA}} \end{array}$$

with ε_*r*_ (solvent's relative permittivity), ε_0_ (vacuum's permittivity, 8.8542 · 10^−12^ C V^−1^ m^−1^), *e* (elementary charge, 1.6022 · 10^−19^ C), and *r*_*DA*_ (donor-acceptor distance of the corresponding centroids of the molecule parts).

For *N,N*-dimethylaniline-hydrocarbon dyad conformers with a *syn*-orientation, the Rehm-Weller approximation with literature redox potentials of the constituting donor and acceptor moieties (Kavarnos and Turro, 1986) and MM2-calculated donor-acceptor distances *r*_*DA*_ reveals that Δ*G*_*PIET*_ is exergonic in all cases (Table 2).

**Figure 8:**
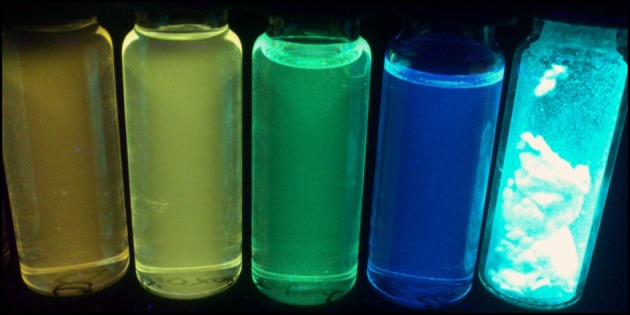
Dyad **5a** in the solid state and in cyclohexane, toluene, 1,4-dioxane, and dichloromethane solutions (from left to right) under a handheld UV lamp (λ_*exc*_ = 365 nm).

The peculiar emission behavior of the dyads **5a, b, g-j** can already be seen upon eyesight under a handheld UV lamp as shown for dyad **5a** in solvents of different polarity and in the solid state (Figure 8). A pronounced positive emission solvatochromism, that is, a red shift of the emission with increasing polarity, immediately becomes apparent. The solid state emission band is very broad and unstructured, and the maximum is detected at 474 nm (λ_*exc*_ = 391 nm) (see Figure S32) and luminesces intensively blue with an external quantum yield *Φ*_*f*_ = 0.320, as determined by an integrating sphere. The broad structureless emission accounts for π-π-stacking of anthracene moieties, as shown for single crystals of 1-acetyl-3-phenyl-5-(9-anthryl)-2-pyrazoline (Feng et al., 2014). As a consequence, the dyad **5a** preferentially adopts the *anti*-conformation in the solid state, where the exciplex emission is absent.

The change of dipole moment from ground to excited state μ_*E*_ − μ_*G*_ can be calculated using the Lippert-Mataga equation (Equation 3),

(3) $$\begin{array}{r} {ṽ}_{a}-{ṽ}_{e}=\frac{2\Delta f}{4\pi \varepsilon_{0}hca^{3}}{\left(\mu_{E}-\mu_{G}\right)}^{2}+const, \end{array}$$

where $\tilde{\nu }$_*a*_ and $\tilde{\nu }$_*e*_ are the absorption and emission maxima (in cm^−1^), ε_0_ is the vacuum permittivity constant (8.8542 · 10^−12^AsV^−1^m^−1^), *c* is the speed of light (2.9979 · 10^10^ cms^−1^), *h* is the Planck's constant (6.6256 · 10^−34^Js^−1^), and the orientation polarizability Δ*f* (Equation (4),

(4) $$\begin{array}{r} \Delta f=\;\frac{\varepsilon_{r}-1}{2\varepsilon_{r}+1}-\;\frac{n^{2}-1}{2n^{2}+1} \end{array}$$

The solvent orientation polarizability Δ*f* of four representative solvents (1,4-dioxane, dichloromethane, acetone, and acetonitrile) gives a very good fit (*r*^2^ = 0.9594) using the Lippert-Mataga plot (Mataga et al., 1956; Lippert, 1957) (see Figure S42), the linear correlation of the Stokes shifts Δ$\tilde{\nu }$ with the relative permittivity ε_*r*_ and the optical refractive index *n* of the corresponding solvent (for details, see Supplementary Material). Since *syn*- and *anti*-conformers might be involved in the electronic ground and excited states, we decided to calculate the Onsager radii *a* for both *syn-* and *anti*-conformations from the optimized ground state structures of dyad **5a** by DFT calculations (Frisch et al., 2016) (vide infra). Assuming a *syn*-conformation for dyad **5a** with an Onsager radius of 3.42 Å (3.42 · 10^−10^ m), a change of dipole moment upon excitation from the ground state to the emissive exciplex state Δμ of 6.02 D (2.008 · 10^−29^ Cm) can be calculated. Likewise, for an *anti*-conformation for dyad **5a** with an Onsager radius of 7.92 Å (7.92 · 10^−10^ m), Δμ can be calculated to 21.19 D (7.067 · 10^−29^ Cm).

### Calculated Electronic Structure

**Figure 9:**
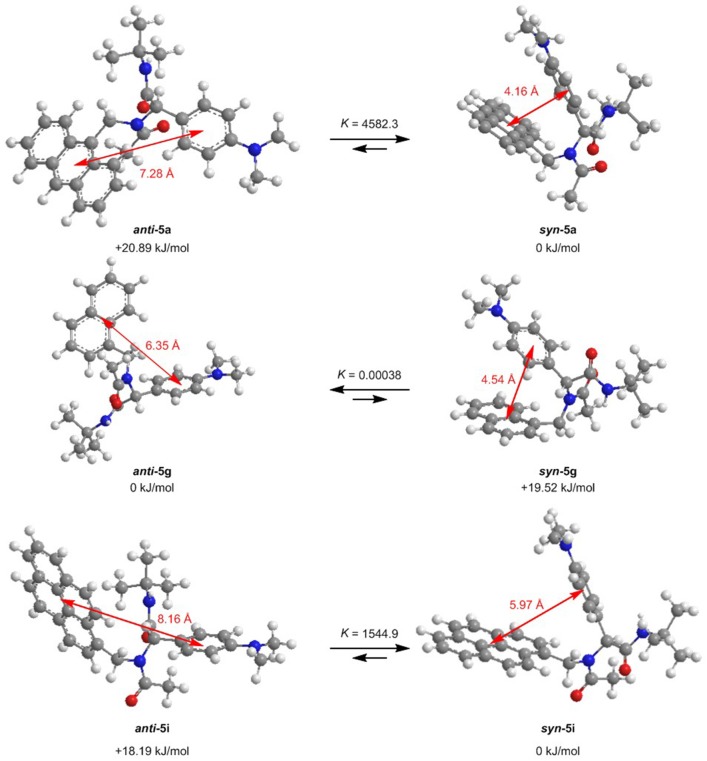
Ground state *anti*-*syn*-conformation equilibria (Δ*G, K*) and donor-acceptor centroid distances according to DFT calculations on the minimum energy conformers of dyads **5a**, **5g**, and **5i** (B3LYP/6-311++G^**^ at *T* = 298 K).

For further elucidation of the electronic structure, the geometries of three representative dyads **5a**, **5g**, and **5i** in their *syn*- and *anti*-conformations were optimized by DFT calculations using Gaussian 16 (Frisch et al., 2016) with the B3LYP functional (Lee et al., 1988; Becke, 1993; Kim and Jordan, 1994; Stephens et al., 1994) and the Pople 6-311++G^**^ basis set (Krishnan et al., 1980), applying vacuum calculations as well as the Polarizable Continuum Model (PCM) with dichloromethane as a solvent (Scalmani and Frisch, 2010) (for details on the DFT calculations, see Supplementary Material). The optimized geometries were verified by frequency analyses of the local minima. Comparison of the calculated ground state energies of the *syn*- and *anti*-conformers reveals that for the anthracene dyad **5a** and for the pyrene dyad **5i**, the *syn*-conformers are stabilized while for the naphthalene dyad **5g**, the *anti*-conformer is slightly favored (Figure 9). Equilibrium constants account for a contribution of both conformers in the absorption and, thus, emission behavior of the dyads **5a**, **5g**, and **5i**. The Onsager radii *a* for the *syn-* and *anti*-ground state conformations of dyad **5a** were estimated from the calculated geometry optimization, which led to the Lippert-Mataga plot to the calculation of the change of dipole moment Δμ between the electronic ground state and the vibrationally relaxed exciplex state (vide supra).

Based on the optimized ground state structures of the *anti*- and *syn*-conformers, the electronic absorptions of the three dyads **5a**, **5g**, and **5i** were calculated on the level of TDDFT theory employing the gradient-corrected exchange and correlation Perdew-Burke-Ernzerhof functionals PBE1PBE/6-31G^**^) (Krishnan et al., 1980; Perdew et al., 1996, 1997; Adamo and Barone, 1999; Ernzerhof and Scuseria, 1999), with dichloromethane (IEFPCM) (Scalmani and Frisch, 2010) as a solvent (for details on the TDDFT calculations, see Table S4). The TDDFT calculation of structure *syn*-**5a** gives a lowest energy transition at 431 nm for the S_1_ Franck-Condon absorption, which is represented to 99% as a HOMO to LUMO transition with a significant oscillatory strength of 0.0167 due to coefficient density on both donor and acceptor moieties. This transition represents considerable charge transfer character from the *N,N*-dimethylaniline substituent to the anthracene unit. The experimental spectrum displays an intense structureless band with a maximum at 391 nm, which is in agreement with an excitation of the anthracene part. The calculated S_2_ Franck-Condon absorption, which is represented to 98% as a HOMO-1 to LUMO transition with an oscillatory strength of 0.0850, accounts for a dominant anthracene excitation and appears at 388 nm. This transition correlates with the anthracene-type transition at 371 nm in the experimental spectrum. Likewise, the TDDFT calculation of structure *anti*-**5a** gives a lowest energy transition at 415 nm for the S_1_ Franck-Condon absorption, which is represented to 99% as a HOMO to LUMO transition with a significant oscillatory strength of 0.003, due to coefficient density on both donor and acceptor moieties. This transition represents considerable charge transfer character from the *N,N*-dimethylaniline substituent to the anthracene unit. The experimental spectrum displays an intense structureless band with a maximum at 391 nm, which is in agreement with an excitation of the anthracene part.

**Figure 10:**
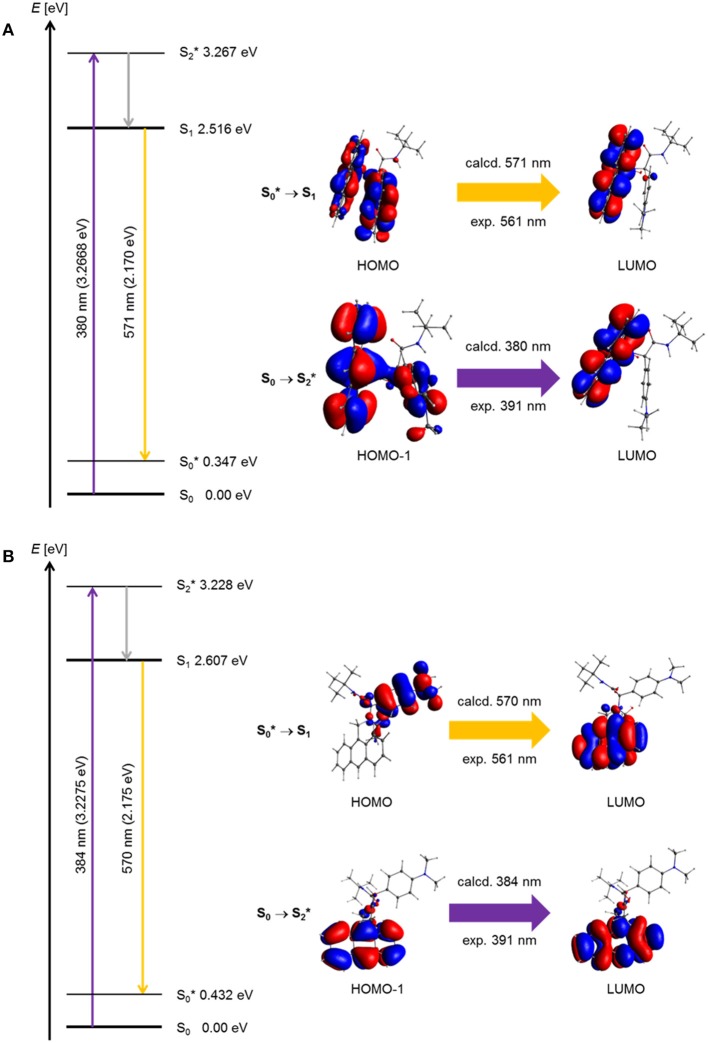
Jablonski diagrams of dyads **(A)** *syn*-**5a** and **(B)** *syn*-**5b** with assignment of the FMO-transitions in the experimental and calculated longest wavelength absorption bands and the exciplex emission bands [*E*(S_0_) = 0 eV; PBE1PBE 6-31G^**^ IEFPCM CH_2_Cl_2_, isosurface value at 0.03 a.u.].

For a general discussion of a model system, the absorption and exciplex emission of dyads *syn*-**5a** and *anti*-**5a** were visualized by plotting in a Jablonski term scheme (Figure 10). Starting for structure *syn*-**5a** from the ground state S_0_ according to TDDFT calculations Franck-Condon absorption to the vibrationally excited ${\text{S}}_{2}^{\;*}$ state proceeds at 380 nm (experiment: 391 nm) as represented by a HOMO-1-LUMO transition, which simultaneously comprises local excitation in the anthracene moiety and coefficient density transfer from *N,N*-dimethylaniline to anthracene (Figure 10A). The vibrationally relaxed S_1_ state lies 0.751 eV lower in energy. This geometry corresponds to that of the vibrationally excited state ${\text{S}}_{0}^{\;*}$. Therefore, the excitation from the vibrationally excited ground state ${\text{S}}_{0}^{\;*}$ to the relaxed first excited state S_1_ translates into the process of exciplex fluorescence, which proceeds at 571 nm (experiment: 561 nm). The geometry of the vibrationally excited ground state ${\text{S}}_{0}^{\;*}$ lies 0.347 eV above the vibrationally relaxed ground state S_0_ and is slightly tighter. This exciplex emission retransmits coefficient density from the anthracene part to the *N,N*-dimethylaniline unit and locally in the anthracene moiety.

Geometrically, the coefficient density in structure *anti*-**5a** is clearly separated in HOMO and LUMO (Figure 10B). However, a similar Franck-Condon absorption energy to the vibrationally excited ${\text{S}}_{2}^{\;*}$ state proceeds at 384 nm (experiment: 391 nm), and an almost identical exciplex emission from the vibrationally relaxed S_1_ state to the vibrationally excited ${\text{S}}_{0}^{\;*}$ state is calculated at 570 nm (experiment: 561 nm). As can be seen from the calculated oscillatory strength (vide supra), the dominant absorption of the *anti*-**5a** conformer is a local excitation of the anthracene moiety, whereas the exciplex emission is completely charge transfer in its character. Expectedly, the oscillatory strength for this process is considerably smaller. Therefore, the experimentally observed exciplex emission preferentially might occur rather from the *syn*-conformer.

Indeed, the simultaneous occurrence of exciplex and hydrocarbon emission in all dyads **5** represents an interesting design principle of novel types of dually emissive bichromophores, arising from selective local excitation followed by acceptor emission as well as redshifted exciplex emission.

## Conclusion

The Ugi 4CR is an excellent synthetic tool for accessing libraries of donor-acceptor dyads that are covalently ligated, yet, electronically decoupled in the ground state, but electronically coupled in the excited state by virtue of the appearance of exciplex emission. The placement of donor and acceptor moieties has been probed in this study for *N,N*-dimethylaniline as a donor moiety and anthracene, naphthalene, and pyrene as hydrocarbon acceptors with favorable redox potentials for enabling exergonic photo-induced intramolecular electron transfer (PIET) upon local hydrocarbon excitation and estimated by Rehm-Weller approximation. The polar nature of the excited state responsible for exciplex emission is unambiguously assigned by emission solvatochromicity measurement and finally by Lippert-Mataga analysis of the Stokes shifts. The change of dipole moment upon excitation from the ground state to the emissive exciplex state can be calculated to Δμ = 6.02 D for the *syn*-conformer of the *N,N*-dimethylaniline-anthracene dyad and to Δμ = 21.19 D for the corresponding *anti*-conformer. For the same dyad, TDDFT calculations with the gradient-corrected exchange and correlation Perdew-Burke-Ernzerhof functionals PBE1PBE were carried out, allowing a qualitative assignment of the experimental absorption and emission bands and, thus, rationalizing the particular relevance of the exciplex' electronic structure. In conclusion, the synthetic scaffold concept employing the isonitrile-based Ugi 4CR enables the design and photophysical proof of concept of novel types of unimolecular exciplex emitters. In principle, even unimolecular multichromophore arrays can be quickly assembled according to the same principle. Synthetic and photophysical studies of dually emissive unimolecular multichromophores are currently underway.

## Experimental

### General Considerations

All reactions were performed in oven-dried Schlenk vessels under a nitrogen atmosphere. The employed solvents were dried with a solvent drying system *MB-SPS 800* by *M. Braun Inertgas-Systeme GmbH* and used as obtained. Commercial grade reagents were used as supplied without further purification and were purchased from abcr GmbH & Co. KG, Acros Organics, Alfa Aesar GmbH & Co. KG, Sigma-Aldrich Chemie GmbH. The purification of Ugi-compounds **5** was performed by column chromatography on silica gel 60 M (0.04–0.063 mm) from Macherey-Nagel GmbH & Co. KG using flash technique under pressure of 2 bar. The crude mixtures were adsorbed on Celite^®^ 545 from Carl Roth GmbH Co. KG before chromatographic purification. The reaction progress was monitored qualitatively using TLC Silica gel 60 F254 aluminum sheets obtained from Merck KGaA, Darmstadt. The spots were detected with UV light at 254 nm and using an iodine chamber. ^1^H, ^13^C, and 135-DEPT ^13^C NMR spectra were recorded on Bruker Avance DRX 300 and Bruker Avance DRX 500. CDCl_3_ and DMSO-d_6_ were used as deuterated solvents. The resonances of the solvents were locked as internal standards (CDCl_3_: ^1^H δ 7.27, ^13^C δ 77.23; DMSO-d_6_: ^1^H δ 2.50, ^13^C δ 39.5). The muliplicities of the signals were abbreviated as follows: s: singlet; d: doublet; dd: doublet of doublets; t: triplet; m: multiplet. The type of carbon nucleus was determined on the basis of 135-DEPT ^13^C NMR spectra. For the description of the ^13^C NMR spectra primary carbon nuclei are abbreviated with CH_3_, secondary carbon nuclei with CH_2_, tertiary carbon nuclei with CH, and quaternary carbon nuclei with C_quat._. MALDI mass spectra were measured on a Bruker Ultraflex spectrometer, ESI mass spectra were measured on an Ion-Trap-API-mass spectrometer of Finnigan LCQ Deca (Thermo Quest). IR spectra were obtained on a Shimadzu IRAffinity-1, which works with the attenuated total reflection (ATR) method. The intensity of signals is abbreviated as follows: s (strong), m (medium), w (weak). UV/Vis spectra were recorded on a Lambda 19 spectrometer (Perkin Elmer) in dichloromethane at *T* = 293 K. Emission spectra were recorded on a Hitachi F-7000 instrument in dichloromethane and concentrations of 10^−6^ mol L^−1^ at *T* = 293 K. Data analysis was done with the software FL solutions 4.0 by Hitachi. The melting points (uncorrected) were measured on a Büchi Melting Point B-540 apparatus. Combustion analyses were carried out on a vario Micro Cube by Elementaranalysentechnik in the Microanalytical Laboratory of the Institut für Pharmazeutische und Medizinische Chemie der Heinrich-Heine-Universität Düsseldorf.

### General Procedure (GP) for the Ugi-4CR Synthesis of Exciplex Bichromophores and Reference Compounds 5

In a 25-mL Schlenk tube with a magnetic stir bar were placed methylamine hydrochloride **1** (0.50 mmol), triethylamine (0.07 mL, 0.50 mmol), and methanol, and the mixture was stirred for 30 min (for experimental details, see Table 3). Then, aldehyde **2** (0.50 mmol) dissolved in dichloromethane was added dropwise, and the reaction mixture was stirred for 1 h. Finally, acetic acid (**3**) (0.50 mmol) and *tert-*butyl isocyanide (**4**) (0.06 mL, 0.50 mmol) were added, and reaction mixture was stirred at room temperature for 24 h. After removal of the solvents, the crude products were purified by flash chromatography on silica gel (*n*-hexane/ethyl acetate) and crystallized from various solvents or solvent mixtures to give analytically pure Ugi-4CR products as colorless to yellow solids.

**Table 3:** Experimental details of the Ugi-4CR synthesis of exciplex bichromophores and reference compounds 5.

**Entry**	**MeOH [mL]**	**CH_**2**_Cl_**2**_ [mL]**	**R^**1**^CH_**2**_NH${\text{NH}}_{\text{3}}^{\text{+}}$Cl^**−**^ 1 [mg] (mmol)**	**R^**2**^CHO 2 [mg] (mmol)**	**Acetic acid (3) [mg] (mmol)**	**Yield [mg] (%)^**a**^**
1	1.00	0.25	123 (0.50) of **1a**	79.8 (0.52) of **2a**	30.0 (0.50)	144 (60) of **5a**
2^b^	1.00	0.25	121 (0.54) of **1b**	105.6 (0.51) of **2b**	30.0 (0.50)	134 (56) of **5b**
3	1.00	0.25	146 (0.60) of **1a**	34.8 (0.60) of **2c**	36.0 (0.60)	32.1 (14) of **5c**
4	1.00	0.25	47.9 (0.70) of **1c**	110 (0.72) of **2a**	42.0 (0.70)	92.4 (43) of **5d**
5^b^	1.00	0.25	137 (0.61) of **1b**	34.8 (0.60) of **2c**	36.0 (0.60)	31.5 (16) of **5e**
6	1.25	0.50	48.0 (0.71) of **1c**	144.4 (0.70) of **2b**	42.0 (0.70)	145 (57) of **5f**
7^c^	1.00	0.25	78.6 (0.50) of **1d**	79.1 (0.53) of **2a**	30.0 (0.50)	91.7 (42) of **5g**
8^b^	1.00	0.25	118 (0.52) of **1b**	78.1 (0.50) of **2d**	30.0 (0.50)	99.1 (46) of **5h**
9	0.75	0.50	142 (0.50) of **1e**	78.1 (0.51) of **2a**	30.0 (0.50)	102 (40) of **5i**
10^b^	1.00	1.00	119 (0.53) of **1b**	117 (0.51) of **2e**	30.0 (0.50)	160 (63) of **5j**

a *Isolated yield after flash chromatography*.

b *Methyl ammonium chloride **1b** was employed as a bishydrochloride and, therefore, the amount of triethylamine was doubled (0.14 mL, 1.00 mmol)*.

c *No triethylamine was added (**1d** was employed as the free base)*.

#### 2-(*N*-(9-Anthrylmethyl)acetamido)-*N*-(tert-butyl)-2-(4-(dimethylamino)phenyl) acetamide (5a)

According to the GP and after chromatography on silica gel (*n*-hexane/ethyl acetate 3:2) and crystallization from hexane/ethanol (50:3), compound **5a** (144 mg, 60%) was obtained as a light yellow solid, Mp 186–187°C, R_*f*_ (*n*-hexane/ethyl acetate 1:1) = 0.40.

^1^H NMR (300 MHz, CDCl_3_): δ = 0.83 (s, 9 H), 1.98 (s, 3 H), 2.90 (s, 6 H), 4.69 (d, *J* = 14.7 Hz, 1 H), 4.69 (s, 1 H), 5.38 (d, *J* = 14.7 Hz, 1 H), 6.26 (d, *J* = 14.1 Hz, 1 H), 6.51–6.69 (m, 2 H), 6.74–6.92 (m, 2 H), 7.387.63 (m, 4 H), 8.03 (d, *J* = 7.5 Hz, 2 H), 8.22 (d, *J* = 7.2 Hz, 2 H), 8.48 (s, 1 H). ^13^C NMR (75 MHz, CDCl_3_): δ = 24.1 (CH_3_), 28.3 (CH_3_), 40.6 (CH_3_), 41.8 (CH_2_), 51.3 (C_quat_), 62.9 (CH), 112.4 (CH), 122.0 (C_quat_), 124.0 (CH), 125.5 (CH), 127.5 (CH), 128.0 (C_quat_), 128.6 (CH), 129.0 (CH), 129.6 (CH), 131.6 (C_quat_), 131.7 (C_quat_), 150.0 (C_quat_), 168.3 (C_quat_), 173.5 (C_quat_). MALDI-TOF-MS (*m*/*z*): 480.1 ([M]^+^). IR: $\tilde{\nu }$ [cm^−1^] = 3,298 (w); 3,055 (w); 2,957 (w); 2,910 (w); 2,863 (w); 2,806 (w); 1,678 (s); 1,614 (s); 1,549 (m); 1,526 (s); 1,481 (w); 1,445 (m); 1,418 (w); 1,391 (w); 1,358 (m); 1,339 (w); 1,325 (w); 1,306 (w); 1,285 (w); 1,256 (w); 1,227 (m); 1,209 (w); 1,193 (w); 1,188 (w); 1,167 (w); 1,167 (w); 1,140 (w); 1,063 (w); 1,040 (w); 1,024 (w); 988 (w); 949 (w); 881 (m); 841 (w); 827 (w); 812 (w); 791 (s); 735 (s); 694 (w); 660 (w); 642 (w); 625 (w). UV/Vis (CH_2_Cl_2_): λ_*max*_ (ε [Lmol^−1^ cm^−1^]) [nm] = 352 (5,100), 417 (8,300), 391 (7,700). Fluorescence (CH_2_Cl_2_): λ_*max*__,em_ [nm] = 417, 444, 475, 561; Stokes shift Δ$\tilde{\nu }$ = 1,600 cm^−1^. Anal. calcd. for C_31_H_35_N_3_O_2_ (481.3): C 77.31, H 7.32, N 8.72; Found: C 77.03, H 7.31, N 8.57.

#### 2-(9-Anthryl)-*N*-(tert-butyl)-2-(*N*-(4-(dimethylamino)phenyl) acetamide) (5b)

According to the GP and after chromatography on silica gel (*n*-hexane/ethyl acetate 3:2) and crystallization from hexane/ethyl acetate (55:5), compound **5b** (134 mg, 56%) was obtained as a yellow solid, Mp 192°C, R_*f*_ (*n*-hexane/ethyl acetate 1:1) = 0.50.

^1^H NMR (300 MHz, CDCl_3_): δ = 1.211.23 (m, 0.5 H), 1.23 (s, 9 H), 2.02 (s, 0.5 H), 2.16 (s, 3 H), 2.63 (s, 6 H), 4.10 (q, *J* = 7.2 Hz, 0.3 H) 4.464.71 (m, 2 H), 5.06 (s, 1 H), 6.02 (d, *J* = 8.7 Hz, 2 H), 6.14 (d, *J* = 8.6 Hz, 2 H), 7.407.59 (m, 4 H), 7.63 (s, 1 H), 7.92 (d, *J* = 7.6 Hz, 2 H), 8.36 (s, 1 H), 8.37 (d, *J* = 8.5 Hz, 2 H). ^13^C NMR (75 MHz, CDCl_3_): δ = 14.35 (CH_3_), 21.18 (CH_3_), 22.94 (CH_3_), 28.71 (CH_3_), 40.74 (CH_3_), 50.14 (CH_2_), 51.94 (C_quat_), 57.62 (CH), 60.53 (CH_2_), 112.27 (CH), 124.96 (CH), 125.14 (CH), 125.73 (C_quat_), 126.25 (C_quat_), 126.56 (CH), 127.28 (CH), 129.58 (CH), 130.57 (CH), 131.63 (C_quat_), 132.29 (C_quat_), 149.24 (C_quat_), 171.05 (C_quat_), 171.28 (C_quat_), 173.01 (C_quat_). MALDI-TOF-MS (*m*/*z*): 480.1 ([M]^+^), 450.9 ([M – 2 CH_3_]^+^), 269.9 ([M – C_11_H_16_N_2_O]^+^). IR: $\tilde{\nu }$ [cm^−1^] = 3,422 (w) 2,979 (w); 2,882 (w); 2,849 (w); 2,799 (w); 1,736 (s); 1,680 (s); 1,620 (s); 1,514 (s); 1,449 (m); 1,406 (m); 1,391 (m); 1,364 (m); 1,256 (m); 1,341 (m); 1,327 (w); 1,314 (w); 1,287 (w); 1,275 (w); 1,258 (w); 1,223 (m); 1,192 (m); 1,169 (w); 1,070 (w); 1,036 (w); 1,022 (w); 997 (w); 968 (w); 949 (w); 939 (w); 908 (w); 853 (w); 812 (w); 785 (m); 741 (s). UV/Vis (CH_2_Cl_2_): λ_*max*_ (ε [Lmol^−1^ cm^−1^]) [nm] = 355 (5,600), 373 (7,800), 393 (6,600). Fluorescence (CH_2_Cl_2_): λ_*max*__,em_ [nm] = 423, 448, 568. Stokes shift Δ$\tilde{\nu }$ = 1,800 cm^−1^. Anal. calcd. for C_31_H_35_N_3_O_2_ 1/6 CH_3_CO_2_C_2_H_5_ (481.3 + 14.7): C 76.59, H 7.35, N 8.51; Found: C 76.24, H 7.21, N 8.54.

#### 2-(*N*-(9-Anthrylmethyl)acetamido)-*N*-tert-butylbutanamide (5c)

According to the GP and after chromatography on silica gel (*n*-hexane/ethyl acetate 2:1) and crystallization from hexane/ethyl acetate (6.5:0.5), compound **5c** (32.1 mg, 14%) was obtained as a yellow crystalline solid, Mp 150°C, R_*f*_ (*n*-hexane/ethyl acetate 2:1) = 0.20.

^1^H NMR (300 MHz, CDCl_3_): δ = 0.52 (s, 9 H), 1.09 (t, *J* = 7.5 Hz, 3 H), 1.35 (m, 1H), 2.04 (s, 3 H), 2.132.34 (m, 1H), 3.48 (t, *J* = 7.2 Hz, 1 H), 4.38 (s, 1 H), 4.91 (d, *J* = 15.0 Hz, 1 H), 6.59 (d, *J* = 15.0 Hz, 1 H), 7.407.65 (m, 4 H), 8.03 (d, *J* = 8.4 Hz, 2 H), 8.33 (d, *J* = 8.8 Hz, 2 H), 8.48 (s, 1 H). ^13^C NMR (75 MHz, CDCl_3_): δ = 12.3 (CH_3_), 22.8 (CH_3_), 24.1 (CH_2_), 27.8 (CH_3_), 42.0 (CH_2_), 50.6 (C_quat_), 61.3 (CH), 123.6 (CH), 125.7 (CH), 127.6 (C_quat_), 128.0 (CH), 129.2 (CH), 130.0 (CH), 131.5 (C_quat_), 131.7 (C_quat_), 169.4 (C_quat_), 171.8 (C_quat_). MALDI-TOF-MS (*m*/*z*): 391.0 ([M]^+^), 347.0 ([M – OC_2_H_3_]^+^). UV/Vis (CH_2_Cl_2_): λ_*max*_ (ε [Lmol^−1^ cm^−1^]) [nm] = 352 (6,800), 370 (10,500), 391 (9,800). Fluorescence (CH_2_Cl_2_): λ_*max*__,em_ [nm] = 418, 443, 471. Stokes shift Δ$\tilde{\nu }$ = 1,700 cm^−1^. Anal. calcd. for C_25_H_30_N_2_O_2_ (390.2): C 76.89, H 7.74, N 7.17; Found: C 76.88, H 7.78, N 6.99.

#### *N*-tert-butyl-2-(4-(dimethylamino)phenyl)-2-(*N*-methylacetamido)acetamide (5d)

According to the GP and after chromatography on silica gel (*n*-hexane/ethyl acetate 1:1) and crystallization from hexane/ethyl acetate (25:2.5), compound **5d** (92.4 mg, 43%) was obtained as a light brown solid, Mp 155°C, R_*f*_ (*n*-hexane/ethyl acetate 1:1) = 0.10.

^1^H NMR (300 MHz, CDCl_3_): δ = 1.30 (s, 9 H), 2.09 (s, 3 H), 2.80 (s, 3 H), 2.93 (s, 6 H), 5.52 (s, 1H), 6.07 (s, 1 H), 6.66 (d, *J* = 8.7 Hz, 2 H), 7.15 (d, *J* = 8.7 Hz, 2 H). ^13^C NMR (75 MHz, CDCl_3_): δ = 22.36 (CH_3_), 28.84 (CH_3_), 33.16 (CH_3_), 40.52 (CH_3_), 51.67 (C_quat_), 60.13 (CH), 112.43 (CH), 122.96 (C_quat_), 130.44 (CH), 150.36 (C_quat_), 169.84 (C_quat_), 171.56 (C_quat_). MALDI-TOF-MS (m/z): 304.3 ([M]^+^). IR: $\tilde{\nu }$ [cm^−1^] = 3,287 (w); 2.965 (w); 2.918 (w); 2,895 (w); 2,806 (w); 1,680 (s); 1,612 (s); 1,551 (m); 1,522 (s); 1,503 (w); 1,474 (w); 1,443 (w); 1,431 (w); 1,404 (m); 1,387 (w); 1,375 (w); 1,350 (m); 1,281 (w); 1,254 (w); 1,227 (m); 1,204 (w); 1,182 (w); 1,169 (w); 1,144 (w); 1,126 (w); 1,065 (w); 1,022 (m); 959 (w); 945 (m); 829 (w); 812 (w); 797 (m); 745 (w); 694 (w); 640 (w). UV/Vis (CH_2_Cl_2_): λ_*max*_ (ε [Lmol^−1^ cm^−1^]) [nm] = 268 (21,000). Fluorescence (CH_2_Cl_2_): λ_*max*__,em_ [nm] = 353. Stokes shift Δ$\tilde{\nu }$ = 9,000 cm^−1^. Anal. calcd. for C_17_H_27_N_3_O_2_ (305.2): C 66.85, H 8.91, N 13.76; Found: C 66.58, H 8.86, N 13.46.

#### *N*-tert-butyl-2-(*N*-(4-(dimethylamino)benzyl)acetamido)butanamide (5e)

According to the GP and after chromatography on silica gel (*n*-hexane/ethyl acetate 2:1) and crystallization from hexane/ethyl acetate (5:0.5), compound **5e** (31.5 mg, 16%) was obtained as a dark yellow solid, Mp 106°C, R_*f*_ (*n*-hexane/ethyl acetate 1:1) = 0.30.

^1^H NMR (300 MHz, CDCl_3_): δ = 0.80 (t, *J* = 7.3 Hz, 3 H), 1.25 (s, 9 H), 1.731.98 (m, 2 H), 2.06 (s, 3 H), 2.88 (s, 6 H), 4.48 (s, 2 H), 4.584.67 (m, 1 H), 6.34 (s, 1 H), 6.64 (d, *J* = 8.7 Hz, 2 H), 7.00 (d, *J* = 8.6 Hz, 2 H). ^13^C NMR (75 MHz, CDCl_3_): δ = 11.1 (CH_3_), 21.9 (CH_2_), 22.6 (CH_3_), 28.8 (CH_3_), 40.6 (CH_3_), 48.9 (CH_2_), 51.2 (C_quat_), 60.5 (CH), 112.9 (CH), 125.3 (C_quat_), 127.2 (CH), 150.0 (C_quat_), 170.1 (C_quat_), 173.1 (C_quat_). MALDI-TOF-MS (*m*/*z*): 332.0 ([M]^+^). UV/Vis (CH_2_Cl_2_): λ_*max*_ (ε [Lmol^−1^ cm^−1^]) [nm] = 264 (14,500), 321 (1,300). Fluorescence (CH_2_Cl_2_): λ_*max*__*,em*_ [nm] = 356. Stokes shift Δ$\tilde{\nu }$ = 9,800 cm^−1^. Anal. calcd. for C_19_H_31_N_3_O_2_ (333.2): C 68.43, H 9.37, N 12.60; Found: C 68.18, H 9.36, N 12.35.

#### 2-(9-Anthryl)-*N*-(tert-butyl)-2-(*N*-methylacetamido)acetamide (5f)

According to the GP and after chromatography on silica gel (*n*-hexane/ethyl acetate 2:1) and crystallization from hexane/ethyl acetate (30:3), compound **5f** (145 mg, 57%) was obtained as a colorless solid, Mp 260–262°C, R_*f*_ (*n*-hexane/ethyl acetate 2:1) = 0.10.

^1^H NMR (300 MHz, CDCl_3_): δ = 1.21 (s, 9 H), 2.22 (s, 3 H), 2.85 (s, 3 H), 4.95 (s, 1 H), 7.397.58 (m, 4 H), 7.49 (s, 1 H), 7.988.05 (m, 2 H), 8.28 (d, *J* = 9.0 Hz, 2 H), 8.52 (s, 1 H). ^13^C NMR (75 MHz, CDCl_3_): δ = 22.4 (CH_3_), 28.7 (CH_3_), 33.3 (CH_3_), 51.86 (C_quat_), 56.6 (CH), 125.0 (CH), 125.3 (CH), 126.2 (C_quat_), 127.3 (CH), 129.6 (CH), 130.4 (CH), 131.7 (C_quat_), 132.2 (C_quat_), 170.6 (C_quat_), 171.3 (C_quat_). MALDI-TOF-MS (*m*/*z*): 363.2 ([M]^+^), 290.2 ([M – NHC(CH_3_)_3_]^+^). IR: $\tilde{\nu }$ [cm^−1^] = 3,327 (w); 3.046 (w); 2,967 (w); 2,930 (w); 2,899 (w); 2,870 (w); 1,684 (s); 1,632 (s); 1,533 (m); 1,472 (w); 1,452 (m); 1,375 (w); 1,358 (m); 1,335 (w); 1,323 (m); 1,281 (w); 1,256 (w); 1,227 (w); 1,188 (w); 1,163 (w); 1,130 (w); 1,107 (w); 1,055 (w); 1,020 (w); 1,001 (w); 991 (w); 961 (w); 941 (w); 926 (w); 897 (m); 874 (w); 851 (m); 820 (w); 787 (w); 733 (s); 638 (w). UV/Vis (CH_2_Cl_2_): λ_*max*_ (ε [Lmol^−1^ cm^−1^]) [nm] = 353 (5,200), 371 (7,700), 391 (6,900). Fluorescence (CH_2_Cl_2_): λ_*max*__,*em*_ [nm] = 420, 445, 475. Stokes shift Δ$\tilde{\nu }$ = 1,800 cm^−1^. Anal. calcd. for C_23_H_26_N_2_O_2_ (362.2): C 76.21, H 7.23, N 7.73; Found: C 75.93, H 7.18, N 7.72.

#### *N*-(tert-butyl)-2-(4-(dimethylamino)phenyl)-2-(*N*-(1-naphthylmethyl)acetamide) acetamide (5g)

According to the GP and after chromatography on silica gel (*n*-hexane/ethyl acetate 1:1) and crystallization from hexane/ethyl acetate (30:2) compound **5g** (91.7 mg, 42%) was obtained as a colorless solid, Mp 174°C, R_*f*_ (*n*-hexane/ethyl acetate 1:1) = 0.50.

^1^H NMR (300 MHz, CDCl_3_): δ = 1.34 (s, 9 H), 2.01 (s, 3 H), 2.77 (s, 6 H), 4.875.32 (m, 2 H), 5.53 (s, 1 H), 5.90 (s, 1 H), 6.42 (d, *J* = 8.5 Hz, 2 H), 7.19 (d, *J* = 8.5 Hz, 2 H), 7.267.34 (m, 2 H), 7.377.50 (m, 2 H), 7.557.67 (m, 1 H), 7.737.86 (m, 2 H). ^13^C NMR (75 MHz, CDCl_3_): δ = 22.3 (CH_3_), 28.8 (CH_3_), 40.4 (CH_3_), 47.9 (CH_2_), 51.7 (C_quat_), 62.9 (CH), 112.3 (CH), 122.1 (C_quat_), 122.3 (CH), 123.4 (CH), 125.5 (CH), 125.7 (CH), 126.1 (CH), 127.2 (CH), 128.9 (CH), 130.3 (C_quat_), 130.9 (CH), 133.0 (C_quat_), 133.5 (C_quat_), 150.4 (C_quat_), 169.9 (C_quat_), 172.9 (C_quat_). MALDI-TOF-MS (*m*/*z*): 430.1 ([M]^+^). IR: $\tilde{\nu }$ [cm^−1^] = 3,314 (w); 3,049 (w); 2,968 (w); 2,912 (w); 2,886 (w); 2,808 (w); 1,676 (m); 1,632 (s); 1,616 (m); 1,601 (w); 1,541 (m); 1,522 (m); 1,507 (w); 1,477 (w); 1,447 (w); 1,423 (m); 1,391 (w); 1,358 (m); 1,319 (w); 1,261 (w); 1,250 (w); 1,227 (m); 1,206 (w); 1,190 (m); 1,165 (w); 1,126 (w); 1,063 (w); 1,047 (w); 1,032 (w); 978 (w); 939 (w); 827 (w); 802 (m); 789 (s); 772 (s); 750 (w); 735 (w); 687 (w); 640 (w); 625 (w). UV/Vis (CH_2_Cl_2_): λ_*max*_ (ε [Lmol^−1^ cm^−1^]) [nm] = 270 (24,000). Fluorescence (CH_2_Cl_2_): λ_*max*__,*em*_ [nm] = 337, 455. Stokes shift Δ$\tilde{\nu }$ = 7,400 cm^−1^. Anal. calcd. for C_27_H_33_N_3_O_2_ (431.3): C 75.14, H 7.71, N 9.74; Found: C 75.15, H 7.58, N 9.67.

#### *N*-tert-butyl-2-(*N*-(4-(dimethylamino)benzyl)acetamido)-2-(1-naphthyl)acetamide (5h)

According to the GP and after chromatography on silica gel (*n*-hexane/ethyl acetate 1:1) and crystallization from hexane/ethyl acetate (33:2.5), compound **5h** (99.1 mg, 46%) was obtained as a light brown solid, Mp 179°C, R_*f*_ (*n*-hexane/ethyl acetate 1:1) = 0.50.

^1^H NMR (300 MHz, CDCl_3_): δ = 1.57 (s, 9 H), 2.31 (s, 3 H), 2.93 (s, 6 H), 4.714.90 (m, 2 H), 5.76 (s, 1 H), 6.39 (d, *J* = 8.7 Hz, 2 H), 6.57 (d, *J* = 8.7 Hz, 2 H), 7.548.03 (m, 7 H), 8.31 (d, *J* = 8.4 Hz, 1 H). ^13^C NMR (75 MHz, CDCl_3_): δ = 23.0 (CH_3_), 28.8 (CH_3_), 40.8 (CH_3_), 49.9 (CH_2_), 51.9 (C_quat_), 58.0 (CH), 112.4 (CH), 123.9 (CH), 125.2 (CH), 125.6 (C_quat_), 126.2 (CH), 127.1 (CH), 127.2 (CH), 127.9 (CH), 128.9 (CH), 129.7 (CH), 132.0 (C_quat_), 132.8 (C_quat_), 133.8 (C_quat_), 149.3 (C_quat_), 170.2 (C_quat_), 173.0 (C_quat_). MALDI-TOF-MS (*m*/*z*): 430.1 ([M]^+^). IR: $\tilde{\nu }$ [cm^−1^] = 3,647 (w); 3,283 (w); 3,233 (w); 3,069 (w); 2,972 (w); 2,909 (w); 2,801 (w); 2,309 (w); 1,684 (s); 1,632 (m); 1,618 (s); 1,597 (w); 1,558 (m); 1,456 (w); 1,412 (m); 1,364 (m); 1,350 (w); 1,337 (m); 1,314 (w); 1,273 (w); 1,260 (w); 1,225 (m); 1,192 (w); 1,180 (w); 1,142 (w); 1,053 (w); 1,024 (w); 976 (w); 941 (w); 910 (w); 795 (s); 777 (s); 754 (w); 735 (w); 667 (w). UV/Vis (CH_2_Cl_2_): λ_*max*_ (ε [Lmol^−1^ cm^−1^]) [nm] = 265 (19,000). Fluorescence (CH_2_Cl_2_): λ_*max*__,*em*_ [nm] = 334, 472. Stokes shift Δ$\tilde{\nu }$ = 7,800 cm^−1^. Anal. calcd. for C_27_H_33_N_3_O_2_ (431.3): C 75. 14, H 7.71, N 9.74; Found: C 74.88, H 7.67, N 9.46.

#### *N*-tert-butyl-2-(4-(dimethylamino)phenyl)-2-(*N*-(1-pyrenylmethyl)acetamido) acetamide (5i)

According to the GP and after chromatography on silica gel (*n*-hexane/ethyl acetate 1:1) and crystallization from hexane/ethyl acetate (45:3.5), compound **5i** (102 mg, 40%) was obtained as a light brown solid, Mp 217–219°C, R_*f*_ (*n*-hexane/ethyl acetate 1:1) = 0.20.

^1^H NMR (300 MHz, CDCl_3_): δ = 1.24 (t, *J* = 7.1 Hz, 0.6 H), 1.36 (s, 9 H), 2.02 (s, 0.6 H), 2.10 (s, 3 H), 2.51 (s, 6 H), 4.10 (q, *J* = 7.1 Hz, 0.4 H), 5.245.52 (m, 2 H), 5.57 (s, 1 H), 6.04 (s, 1 H), 6.25 (d, *J* = 8.6 Hz, 2 H), 7.17 (d, *J* = 8.5 Hz, 2 H), 7.748.17 (m, 9 H). ^13^C NMR (75 MHz, CDCl_3_): δ = 14.3 (CH_3_), 21.2 (CH_3_), 22.6 (CH_3_), 28.9 (CH_3_), 40.0 (CH_3_), 48.0 (CH_2_), 51.7 (C_quat_), 60.5 (CH_2_), 62.6 (CH), 112.1 (CH), 121.8 (C_quat_), 122.0 (CH), 123.8 (CH), 124.6 (C_quat_), 124.8 (CH), 125.1 (CH), 125.3 (CH), 126.0 (CH), 126.9 (CH), 127.2 (CH), 127.5 (C_quat_), 130.2 (CH), 130.7 (C_quat_), 130.9 (C_quat_), 131.3 (CH), 131.4 (C_quat_), 150.1 (C_quat_), 170.07 (C_quat_), 172.9 (C_quat_), 173.0 (C_quat_). MALDI-TOF-MS (*m*/*z*): 504.2 ([M]^+^). IR: $\tilde{\nu }$ [cm^−1^] = 3,289 (w); 3,040 (w); 2,961 (w); 2,893 (w); 2,805 (w); 1,739 (w); 1,670 (m); 1,612 (s); 1,587 (w); 1,547 (m); 1,522 (s); 1,433 (m); 1,414 (m); 1,391 (w); 1,360 (m); 1,339 (w); 1,304 (w); 1,267 (w); 1,254 (w); 1,225 (m); 1,204 (m); 1,167 (m); 1,132 (w); 1,063 (w); 1,032 (w); 980 (w); 934 (w); 843 (s); 812 (m); 797 (m); 710 (s); 640 (w). UV/Vis (CH_2_Cl_2_): λ_*max*_ (ε [Lmol^−1^ cm^−1^]) [nm] = 317 (13,000), 331 (27,000), 347 (36,000). Fluorescence (CH_2_Cl_2_): λ_*max*__,*em*_ [nm] = 383, 397, 499. Stokes shift Δ$\tilde{\nu }$ = 2,700 cm^−1^. Anal. calcd. for C_33_H_35_N_3_O_2_ 1/5 CH_3_CO_2_C_2_H_5_ (505.3 + 17.6): C 77.58, H 7.05, N 8.02; Found: C 77.11, H 7.00, N 8.02.

#### *N*-(tert-butyl)-2-(*N*-(4-(dimethylamino)benzyl)acetamido)-2-(1-pyrenyl)acetamide (5j)

According to the GP and after chromatography on silica gel (*n*-hexane/ethyl acetate 1:1) and crystallization from hexane/ethyl acetate (62:4.5), compound **5j** (160 mg, 63%) was obtained as a light brown solid, Mp 202–205°C, R_*f*_ (*n*-hexane/ethyl acetate 1:1) = 0.30.

^1^H NMR (300 MHz, CDCl_3_): δ = 1.201.25 (m, 1.2 H), 1.37 (s, 9 H), 2.00 (s, 1.2 H), 2.20 (s, 6 H), 2.21 (s, 3 H), 4.08 (q, *J* = 7.1 Hz, 0.8 H), 4.454.76 (m, 2 H), 5.70 (s, 1 H), 5.70 (d, *J* = 8.5 Hz, 2 H), 6.21 (d, *J* = 8.6 Hz, 2 H), 7.25 (s, 1 H), 7.888.27 (m, 9 H). ^13^C NMR (75 MHz, CDCl_3_): δ = 14.28 (CH_3_), 21.0 (CH_3_), 22.9 (CH_3_), 28.9 (CH_3_), 40.2 (CH_3_), 50.2 (CH_2_), 52.0 (C_quat_), 58.3 (CH), 60.5 (CH_2_), 111.8 (CH), 123.3 (CH), 124.5 (C_quat_), 124.7 (C_quat_), 124.8 (CH), 125.0 (C_quat_), 125.6 (CH), 126.1 (CH) 126.7 (CH), 127.2 (CH), 127.3 (CH), 127.9 (CH), 128.7 (CH), 129.1 (C_quat_), 130.8 (C_quat_), 130.9 (C_quat_), 131.2 (C_quat_), 131.8 (C_quat_), 148.5 (C_quat_), 170.1 (C_quat_), 170.4 (C_quat_), 172.8 (C_quat_). MALDI-TOF-MS (*m*/*z*): 504.2 ([M]^+^). IR: $\tilde{\nu }$ [cm^−1^] = 3,304 (w); 3,040 (w); 2,965 (w); 2,897 (w); 2,884 (w); 2,874 (w); 1,733 (w); 1,684 (m); 1,674 (w); 1,616 (s); 1,549 (m); 1,524 (m); 1,506 (w); 1,468 (w); 1,435 (m); 1423 (m), 1393 (w),1366 (w), 1341 (w), 1314 (w), 1277 (w), 1253 (w), 1225 (m), 1175 (w), 1126 (w), 1,053 (w); 1,034 (w); 980 (w); 972 (w); 943 (w); 910 (w); 845 (s); 812 (w); 799 (m); 758 (w); 710 (w); 681 (w). UV/Vis (CH_2_Cl_2_): λ_*max*_ (ε [Lmol^−1^ cm^−1^]) [nm] = 317 (14,000), 331 (28,000), 348 (38,000). Fluorescence (CH_2_Cl_2_): λ_*max*__,*em*_ [nm] = 380, 395, 538. Stokes shift Δ$\tilde{\nu }$ = 2,400 cm^−1^. Anal. calcd. for C_33_H_35_N_3_O_2_ 1/2 CH_3_CO_2_C_2_H_5_ (505.3 + 44.1): C 76.47, H 7.15, N 7.64; Found: C 76.51, H 7.15, N 8.10.

## Data Availability Statement

All datasets generated for this study are included in the article/Supplementary Material.

## Author Contributions

The project was conceptualized by TM for the MSc thesis of MO, who developed the synthetic approach and conducted the photophysical studies and their evaluation. BM performed all DFT and TDDFT calculations and assigned the experimental absorption and emission bands transitions. Based upon the master thesis of MO, the manuscript was written and corrected by TM and BM.

### Conflict of Interest

The authors declare that the research was conducted in the absence of any commercial or financial relationships that could be construed as a potential conflict of interest.

## Supplementary Material

Supplementary Information contains the optimization of the synthesis of compound **5a**, ^1^H and ^13^C NMR spectra of compounds **5**, the absorption and emission spectra of compounds **5**, the Lippert-Mataga analysis of dyad **5a** and the data and evaluation of the DFT and TDDFT calculations on the *syn*- and *anti*-structures **5a**, **5g**, and **5i**.
