# Supplementary material for: Unimolecular Exciplexes by Ugi Four-Component Reaction
Source: Front Chem. 2019 Nov 1;7:717. doi: 10.3389/fchem.2019.00717 (PMC6838752; doi:10.3389/fchem.2019.00717)
Supplement: Supplementary file 1 [file Data_Sheet_1.PDF]

Supplementary Material

Unimolecular Exciplexes by Ugi Four-component Reaction

Maria Ochs,[1,2] Bernhard Mayer,[1] and Thomas J. J. Müller[1]*

[1]Institut für Organische Chemie und Makromolekulare Chemie, Heinrich-Heine-Universität Düsseldorf, Universitätsstrasse 1, D-40225 Düsseldorf, Germany

[2]Ernst-Berl Institut für Technische und Makromolekulare Chemie, Technische Universität Darmstadt, Alarich-Weiss-Strasse 4, D-64287 Darmstadt, Germany

*** Correspondence:** Thomas J. J. Müller: ThomasJJ.Mueller@hhu.de

Table of contents

1. Optimization of the Synthesis of Compound **5a** 3

2. ^{1}H and ^{13}C NMR Spectra of Compounds **5** 6

2.1 2-(*N*-(9-Anthrylmethyl)acetamido)-*N*-(*tert*-butyl)-2-(4-(dimethylamino)phenyl) acetamide (**5a**) 6

2.2 2-(9-Anthryl)-*N*-(*tert*-butyl)-2-(*N*-(4-(dimethylamino)benzyl)acetamido)acetamide (**5b**) 7

2.3 2-(*N*-(9-Anthrylmethyl)acetamido)-*N*-*tert*-butylbutanamide (**5c**) 8

2.4 *N*-*tert*-Butyl-2-(4-(dimethylamino)phenyl)-2-(*N*-methylacetamido)acetamide (**5d**) 9

2.5 *N*-*tert*-Butyl-2-(*N*-(4-(dimethylamino)benzyl)acetamido)butanamide (**5e**) 10

2.6 2-(9-Anthryl)-*N*-(*tert*-butyl)-2-(*N*-methylacetamido)acetamide (**5f**) 11

2.7 *N*-(*tert*-Butyl)-2-(4-(dimethylamino)phenyl)-2-(*N*-(1-naphthylmethyl)acetamido) acetamide (**5g**) 12

2.8 *N*-*tert*-Butyl-2-(*N*-(4-(dimethylamino)benzyl)acetamido)-2-(1-naphthyl)acetamide (**5h**) 12

2.9 *N*-*tert*-Butyl-2-(4-(dimethylamino)phenyl)-2-(*N*-(1-pyrenylmethyl)acetamido) acetamide (**5i**) 14

2.10 *N*-(*tert*-Butyl)-2-(*N*-(4-(dimethylamino)benzyl)acetamido)-2-(1-pyrenyl)acetamide (**5j**) 14

3. Absorption and Emission Spectra of Compounds **5** 16

3.1 2-(*N*-(9-Anthrylmethyl)acetamido)-*N*-(*tert*-butyl)-2-(4-(dimethylamino)phenyl) acetamide (**5a**) 16

3.2 2-(9-Anthryl)-*N*-(*tert*-butyl)-2-(*N*-(4-(dimethylamino)benzyl)acetamido)acetamide (**5b**) 18

3.3 2-(*N*-(9-Anthrylmethyl)acetamido)-*N*-*tert*-butylbutanamide (**5c**) 19

3.4 *N*-*tert*-Butyl-2-(4-(dimethylamino)phenyl)-2-(*N*-methylacetamido)acetamide (**5d**) 20

3.5 *N*-*tert*-Butyl-2-(*N*-(4-(dimethylamino)benzyl)acetamido)butanamide (**5e**) 21

3.6 2-(9-Anthryl)-*N*-(*tert*-butyl)-2-(*N*-methylacetamido)acetamide (**5f**) 22

3.7 *N*-(*tert*-Butyl)-2-(4-(dimethylamino)phenyl)-2-(*N*-(1-naphthylmethyl)acetamido) acetamide (**5g**) 23

3.8 *N*-*tert*-Butyl-2-(*N*-(4-(dimethylamino)benzyl)acetamido)-2-(1-naphthyl)acetamide (**5h**) 24

3.9 *N*-*tert*-Butyl-2-(4-(dimethylamino)phenyl)-2-(*N*-(1-pyrenylmethyl)acetamido) acetamide (**5i**) 25

3.10 *N*-(*tert*-Butyl)-2-(*N*-(4-(dimethylamino)benzyl)acetamido)-2-(1-pyrenyl)acetamide (**5j**) 26

4. Lippert-Mataga Analysis of Compound **5a** 27

5. DFT and TDDFT Calculations on the *syn*- and *anti*-Structures **5a**, **5g**, and **5i** 28

5.1 Structure *anti*-**5a** 28

5.2 Structure *syn*-**5a** 30

5.3 Structure *anti*-**5g** 32

5.4 Structure *syn*-**5g** 34

5.5 Structure *anti*-**5i** 36

5.6 Structure *syn*-**5i** 39

5.7 TDDFT Calculations (Absorption Bands) of *syn*- and *anti*-Structures **5a**, **5g**, and **5i** 42

5.8 TDDFT Calculations (Absorption and Exciplex Emission Bands) of *syn*- and *anti*-Structures **5a** 47

5.8.1 *anti*-Structure **5a** 47

5.8.2 *syn*-Structure **5a** 51

1. Optimization of the Synthesis of Compound 5a

Table S1. Optimization of the Ugi-4CR synthesis of bichromophore **5a**.

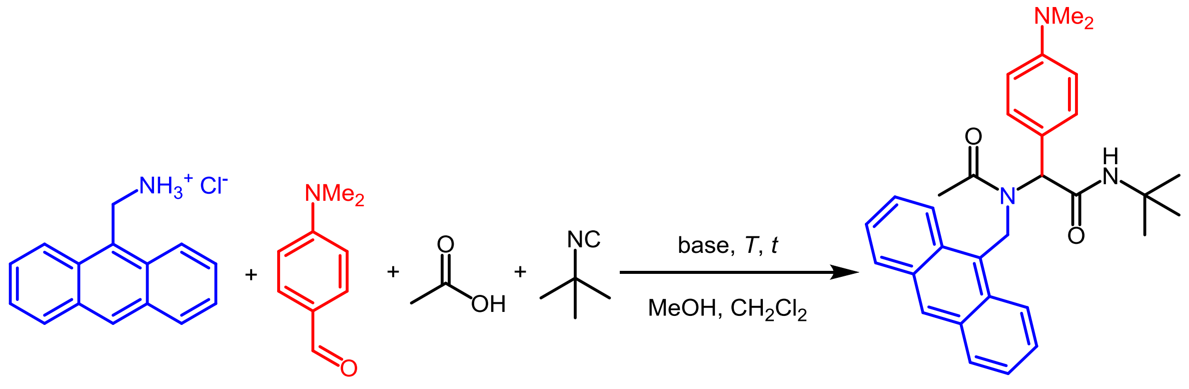

1a **2a** **3** **4** **5a**

Entry	c_0(1a)[a] [M]	c_0(2a)[b] [M]	Base	*T* [°C]	*t* [h]	Isolated yield of compound 5a [%]
1	0.25	0.50	KOH (1.00 equiv)	25	24	18
2	0.25	0.50	Et_3N (2.00 equivs)	25	24	53
3	0.33	1.00	Et_3N (2.00 equivs)	25	24	57
4	0.33	1.00	Et_3N (2.00 equivs)[c]	25	24	60
5	0.33	1.00	Et_3N (2.00 equivs)[d]	25	24	51
**6**	**0.50**	**2.00**	**Et_3N (1.00 equiv)**	**25**	**24**	**60**
7[e]	0.60	2.40	Et_3N (1.00 equiv)	25	24	71
8	0.50	2.00	Et_3N (1.00 equiv)	0	24	8
9	0.50	2.00	Et_3N (1.00 equiv)	40	24	60
10	0.25	-[f]	Et_3N (1.00 equiv)	60	24	22
11	0.50	2.00	Et_3N (1.00 equiv)	25	48	60
12	0.50	2.00	Et_3N (1.00 equiv)	25	98	58

[a]In MeOH. [b]In CH_2Cl_2. [c]$TiCl_4$ (5.00 mol%) was added as a catalyst. [d]$AlCl_3$ (5.00 mol%) was added as a catalyst. [e]The stoichiometric ratio of **1a**:**2a**:**3**:**4** was 1.2:1.2:1:1. [f]Compound **2a** was added neat.

After liberation of the free amine with an auxiliary base and addition of a dichloromethane solution of aldehyde **2**, acetic acid (**3**) and isonitrile **4** were successively added after an hour of stirring at room temp. All reactions were performed on an equistoichiometric scale (1:1:1:1) with respect to compounds **1a**, **2a**, **3** and **4** and the reported yields are isolated yields after chromatographic purification.

In our previous study on donor-acceptor conjugates potassium carbonate could successfully employed for liberating the amine, however, here we found that triethylamine is clearly superior with respect to isolated yields (Table S1, entries 1 and 2). As a consequence further optimization was conducted with triethylamine as a base, where the amount of base did not affect the yield of isolated compound **5a** (Table S1, entries 3-12). Also the addition of catalytic amounts of Lewis acid catalysts for increasing the electrophilicity of the imine intermediate essentially did not affect the yield (Table 1, entries 4 and 5). Expectedly, increasing the substrate concentrations led to a slight increase in yield (Table S1, entries 3 and 4), as well as increasing the ratio of amine and aldehyde to acetic acid and isonitrile (Table S1, entry 7). However, the latter causes a deviation from the preferred equistoichiometric ratio. The temperature window for full conversion within 24 h lies within room temp and 40 °C, with a significant decrease in yield at 0 °C and 60 °C (Table S1, entries 8 and 10). Finally, increasing the reaction time does not affect the yield of compound **5a** (Table S1, entries 6, 11 and 12).

Optimization of the Ugi-4CR synthesis of bichromophore 5a.

In a 25 mL Schlenk tube with a magnetic stir bar were placed methylamine hydrochloride **1a** (123 mg, 0.50), a base, and methanol and the mixture was stirred for 30 min (for experimental details see Table 4). Then, aldehyde **2a** (76.6 mg, 0.50 mmol) dissolved in dichloromethane was added dropwise and the reaction mixture was stirred for 1 h. Finally, acetic acid (**3**) (30 mg, 0.50 mmol) and *tert*-butyl isocyanide (**4**) (0.06 mL, 0.50 mmol) were added and reaction mixture was stirred at the indicated temperature *T* for the time *t*. After removal of the solvents the crude products were purified by flash chromatography on silica gel (*n*-hexane/ethyl acetate) to give analytically pure bichromophore **5a** as a light yellow solid.

Table S2. Experimental details of the Ugi-4CR synthesis of the optimization of the Ugi-4CR synthesis of bichromophore **5a**.

Entry	MeOH [mL]	CH_2Cl_2 [mL]	Base	*T* [°C]	*t* [h]	Yield of 5a [mg] (%)[a]
1	2.00	1.00	28 mg (0.5 mmol) of KOH	25	24	43 (18)
2	2.00	1.00	0.14 mL (1.00 mmol) of NEt_3	25	24	128 (53)
3	1.50	0.50	0.14 mL (1.00 mmol) of NEt_3	25	24	137 (57)
4	1.50	0.50	0.14 mL (1.00 mmol) of NEt_3[b]	25	24	145 (60)
5	1.50	0.50	0.14 mL (1.00 mmol) of NEt_3[c]	25	24	123 (51)
6	1.00	0.25	0.07 mL (0.50 mmol) of NEt_3	25	24	144 (60)
7[d]	1.00	0.25	0.08 mL (0.60 mmol) of NEt_3	25	24	205 (71)
8[b]	1.00	0.25	0.07 mL (0.50 mmol) of NEt_3	0	24	19 (8)
9	1.00	0.25	0.07 mL (0.50 mmol) of NEt_3	40	24	145 (60)
10	2.00	-	0.07 mL (0.50 mmol) of NEt_3	60	24	53 (22)
11	1.00	0.25	0.07 mL (0.50 mmol) of NEt_3	25	48	144 (60)
12	1.00	0.25	0.07 mL (0.50 mmol) of NEt_3	25	98	140 (58)

[a]Isolated yield after flash chromatography. [b]$TiCl_4$ (4.7 mg, 25 μmol) was added as a catalyst. [c]$AlCl_3$ (3.3 mg, 25 μmol) was added as a catalyst. [d]Methyl ammonium chloride **1a** (148 mg, 0.60 mmol), aldehyde **2a** (91.9 mg, 0.60 mmol), acetic acid (**3**) (30 mg, 0.50 mmol), and *tert*-butyl isocyanide (**4**) (0.06 mL, 0.50 mmol) were added in a stoichiometric ratio of 1.2:1.2:1:1.

2. ^{1}H and ^{13}C NMR Spectra of Compounds 5

2.1 2-(*N*-(9-Anthrylmethyl)acetamido)-*N*-(*tert*-butyl)-2-(4-(dimethylamino)phenyl) acetamide (5a)

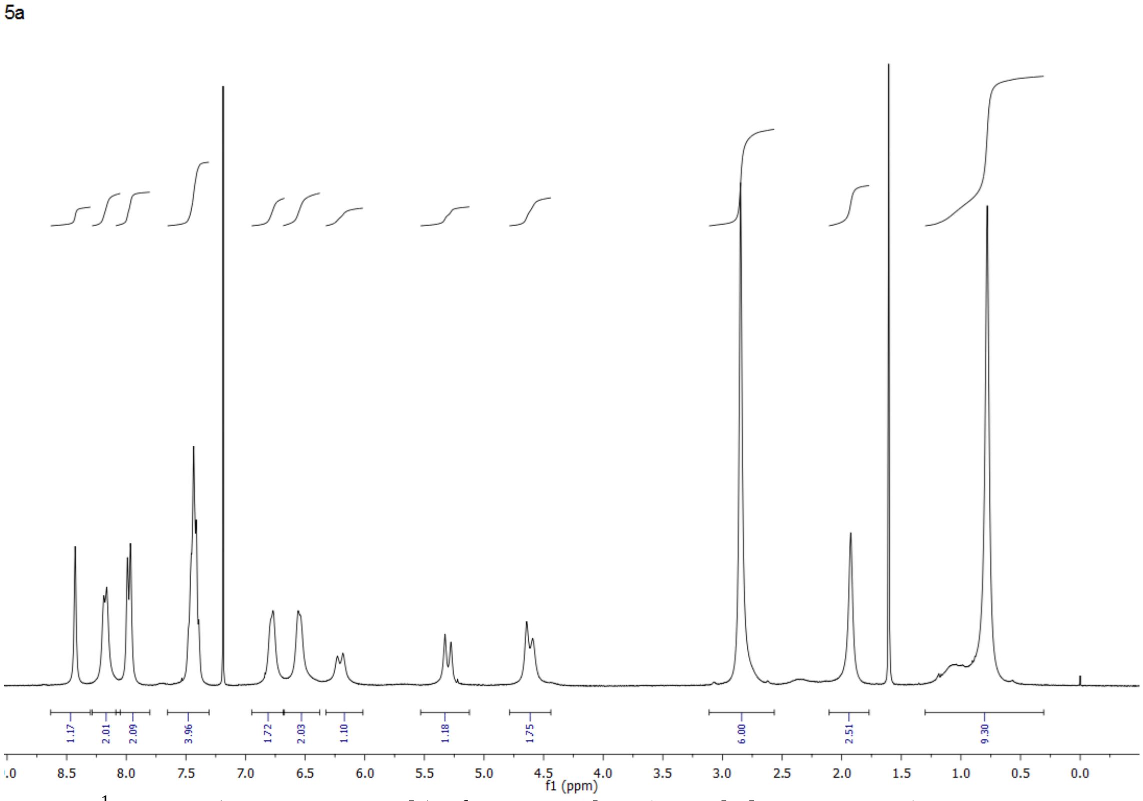

Figure S1. ^{1}H NMR (300 MHz, $CDCl_3$) of compound **5a** (recorded at T = 293 K).

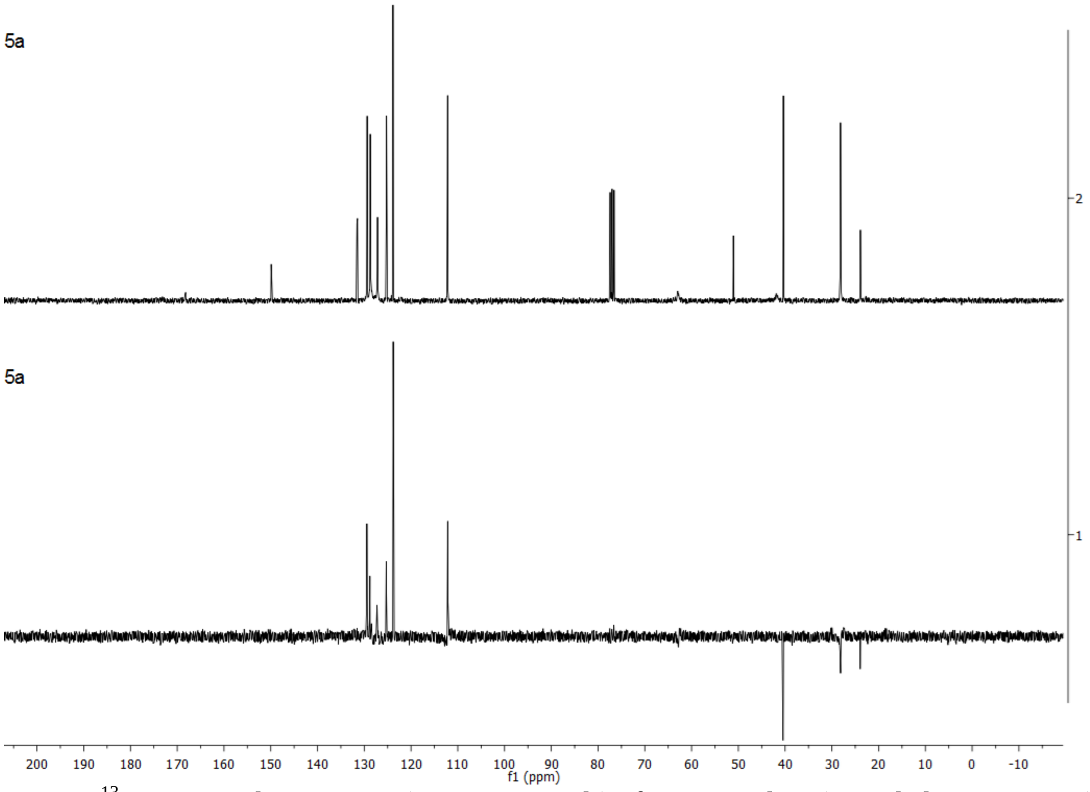

Figure S2. ^{13}C NMR and 135 DEPT (75 MHz, $CDCl_3$) of compound **5a** (recorded at T = 293 K).

2.2 2-(9-Anthryl)-*N*-(*tert*-butyl)-2-(*N*-(4-(dimethylamino)benzyl)acetamido)acetamide (5b)

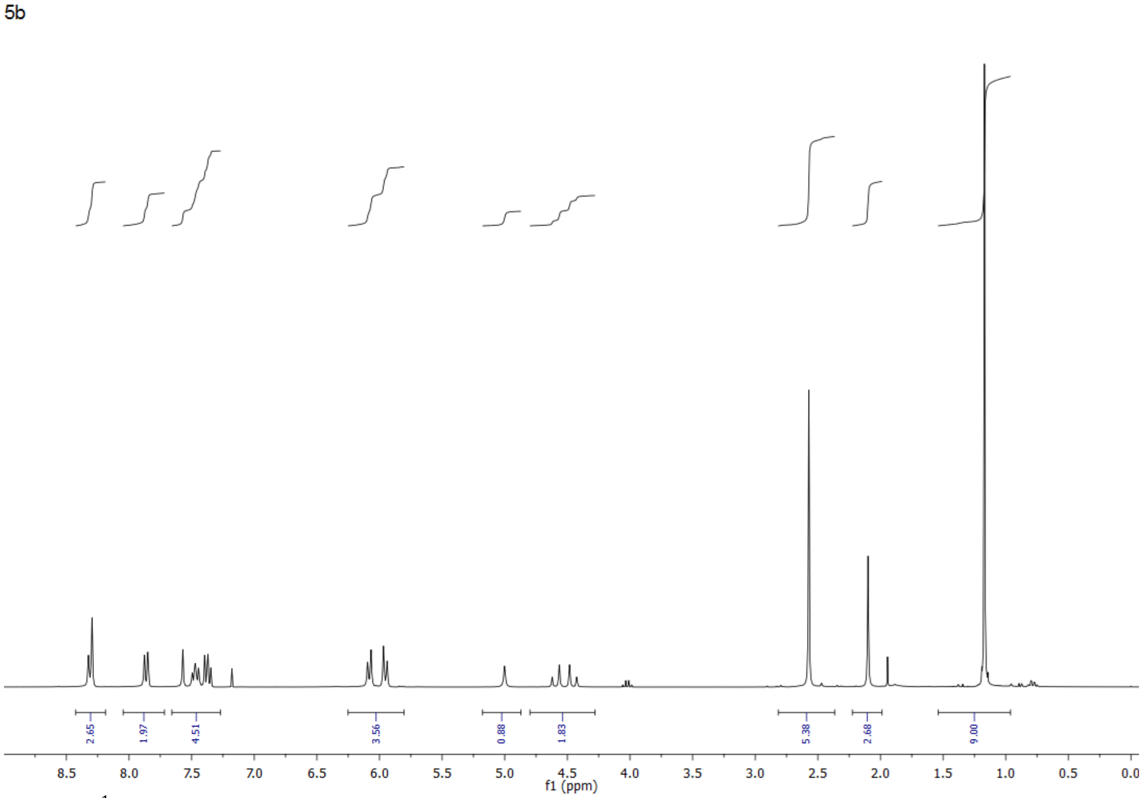

Figure S3. ^{1}H NMR (300 MHz, $CDCl_3$) of compound **5b** (recorded at T = 293 K).

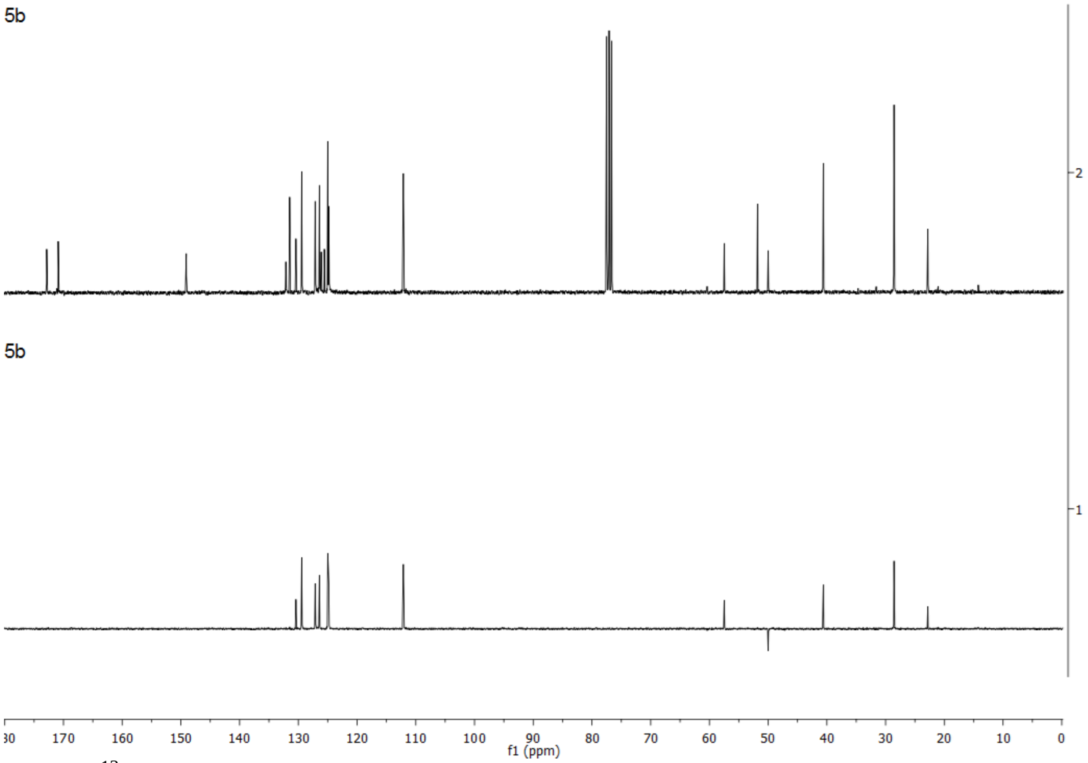

Figure S4. ^{13}C NMR and 135 DEPT (75 MHz, $CDCl_3$) of compound **5b** (recorded at T = 293 K).

2.3 2-(*N*-(9-Anthrylmethyl)acetamido)-*N*-*tert*-butylbutanamide (5c)

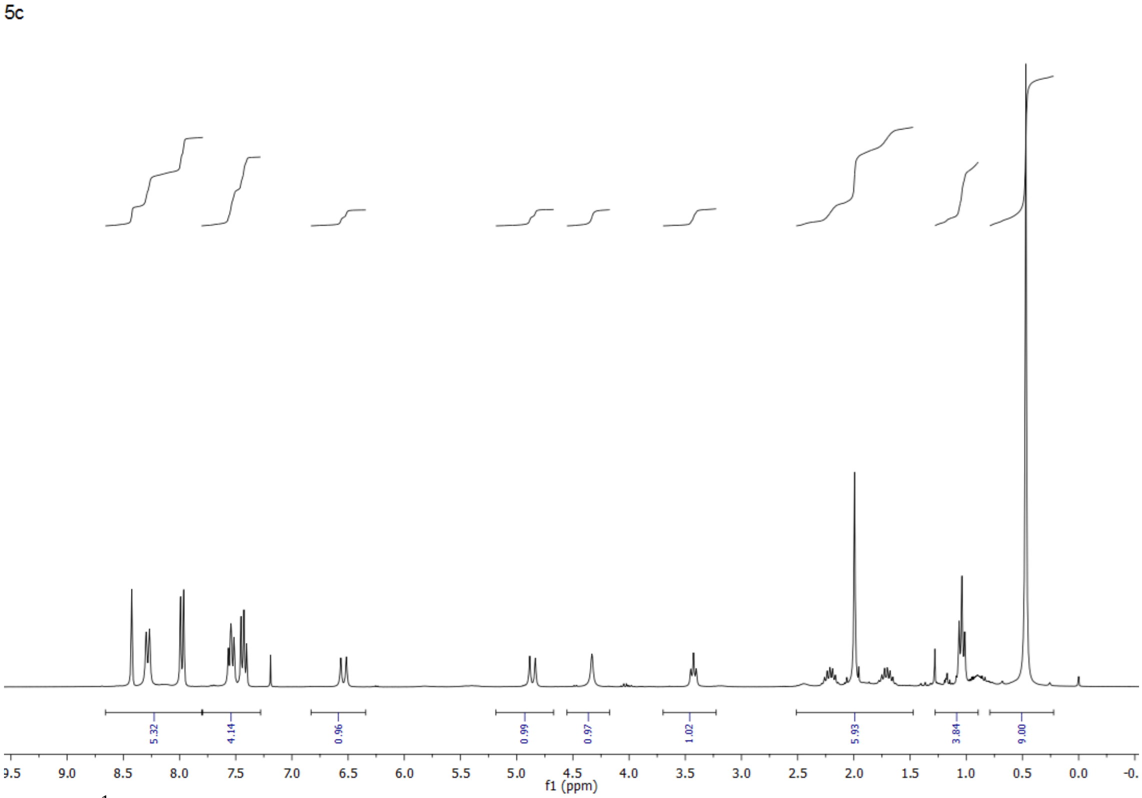

Figure S5. ^{1}H NMR (300 MHz, $CDCl_3$) of compound **5c** (recorded at *T* = 293 K).

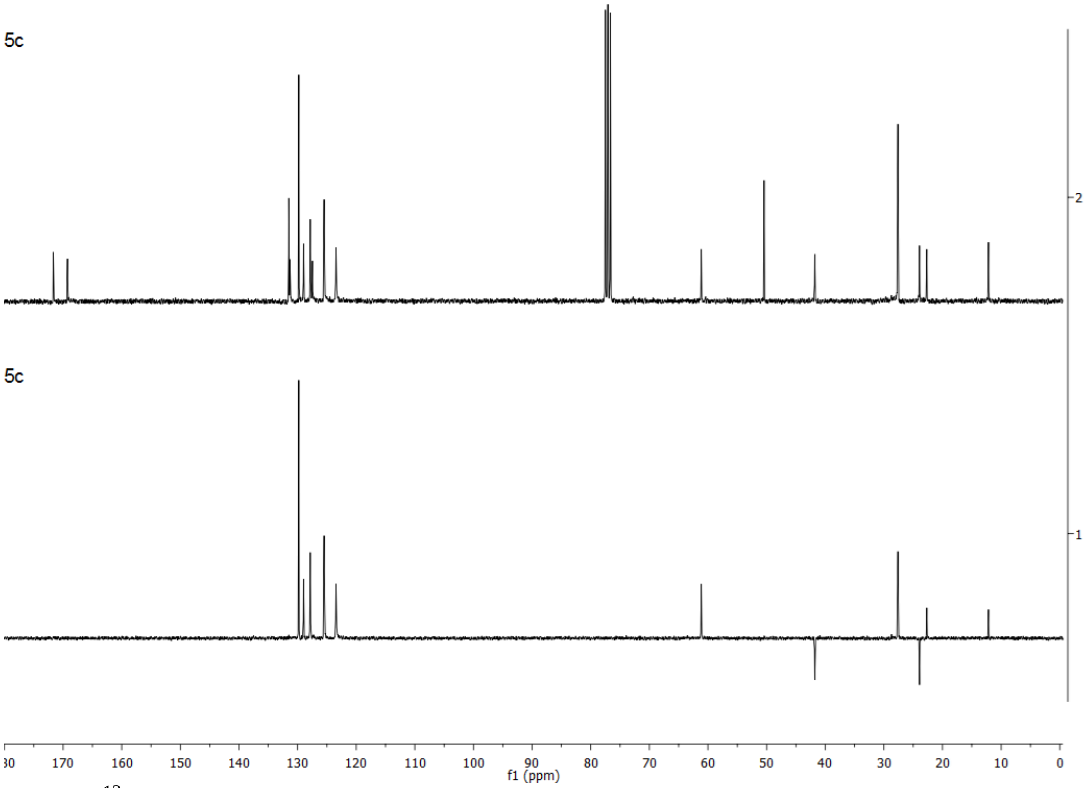

Figure S6. ^{13}C NMR and 135 DEPT (75 MHz, $CDCl_3$) of compound **5c** (recorded at *T* = 293 K).

2.4 *N*-*tert*-Butyl-2-(4-(dimethylamino)phenyl)-2-(*N*-methylacetamido)acetamide (5d)

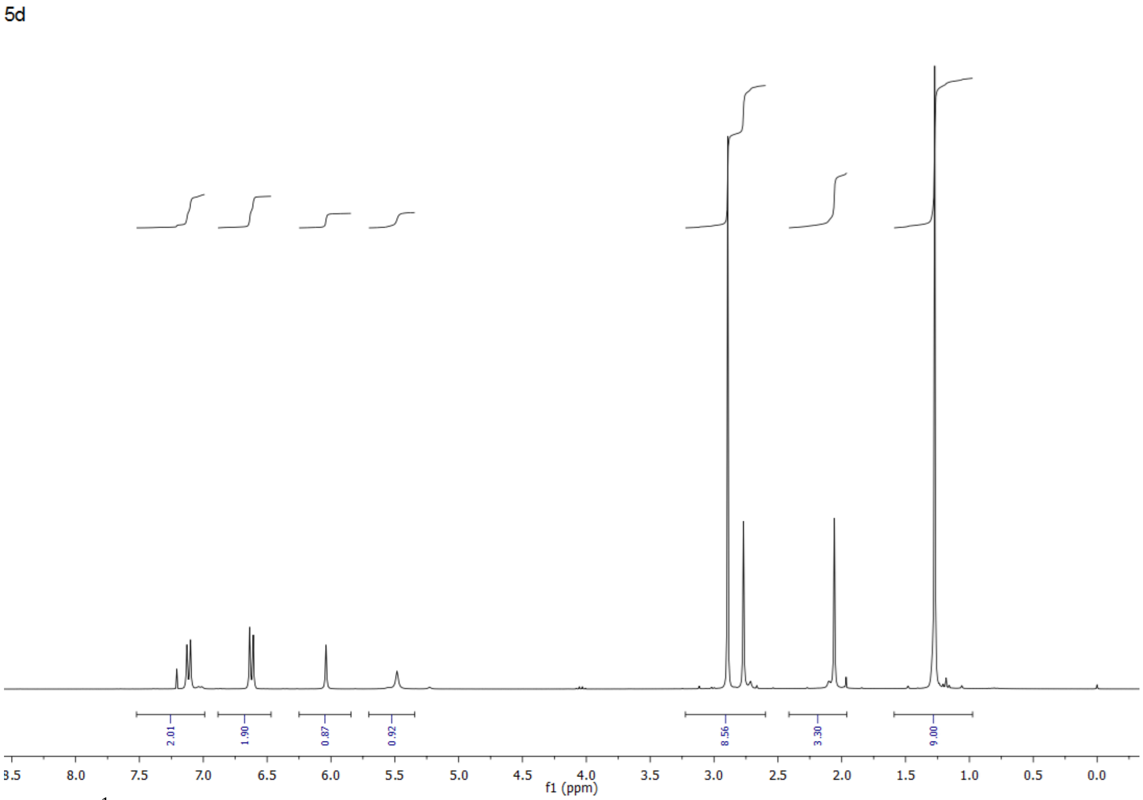

Figure S7. ^{1}H NMR (300 MHz, $CDCl_3$) of compound **5d** (recorded at T = 293 K).

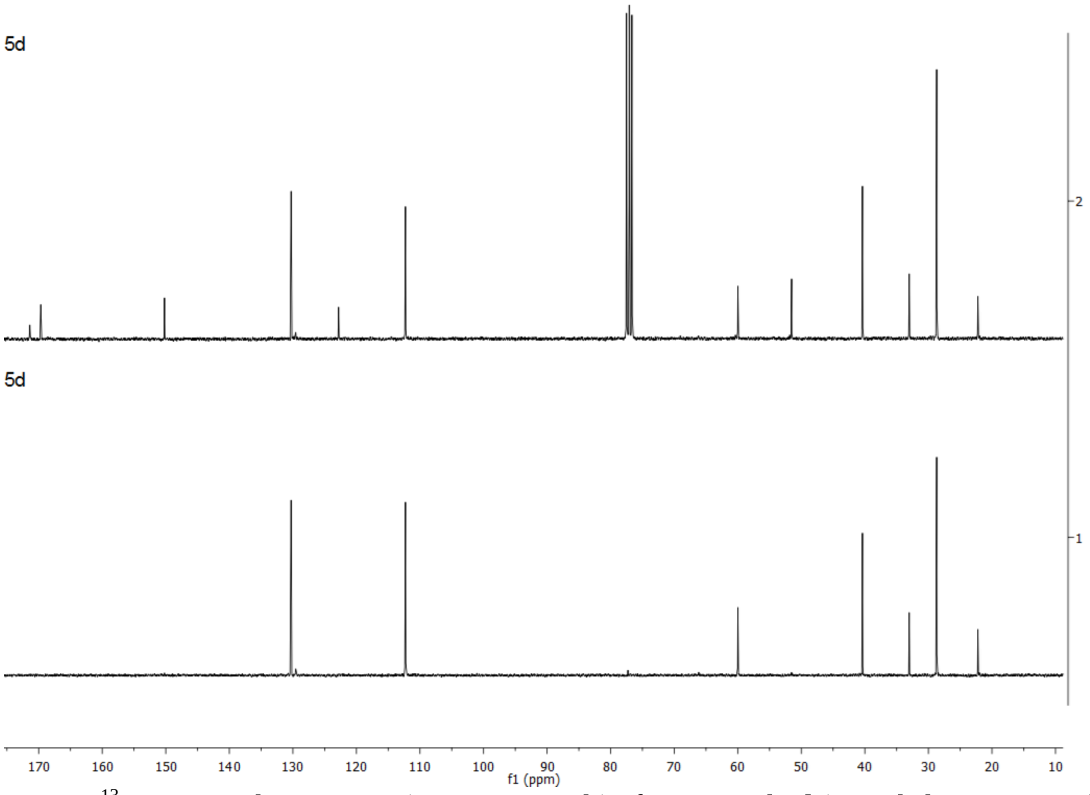

Figure S8. ^{13}C NMR and 135 DEPT (75 MHz, $CDCl_3$) of compound **5d** (recorded at T = 293 K).

2.5 *N-tert*-Butyl-2-(*N*-(4-(dimethylamino)benzyl)acetamido)butanamide (5e)

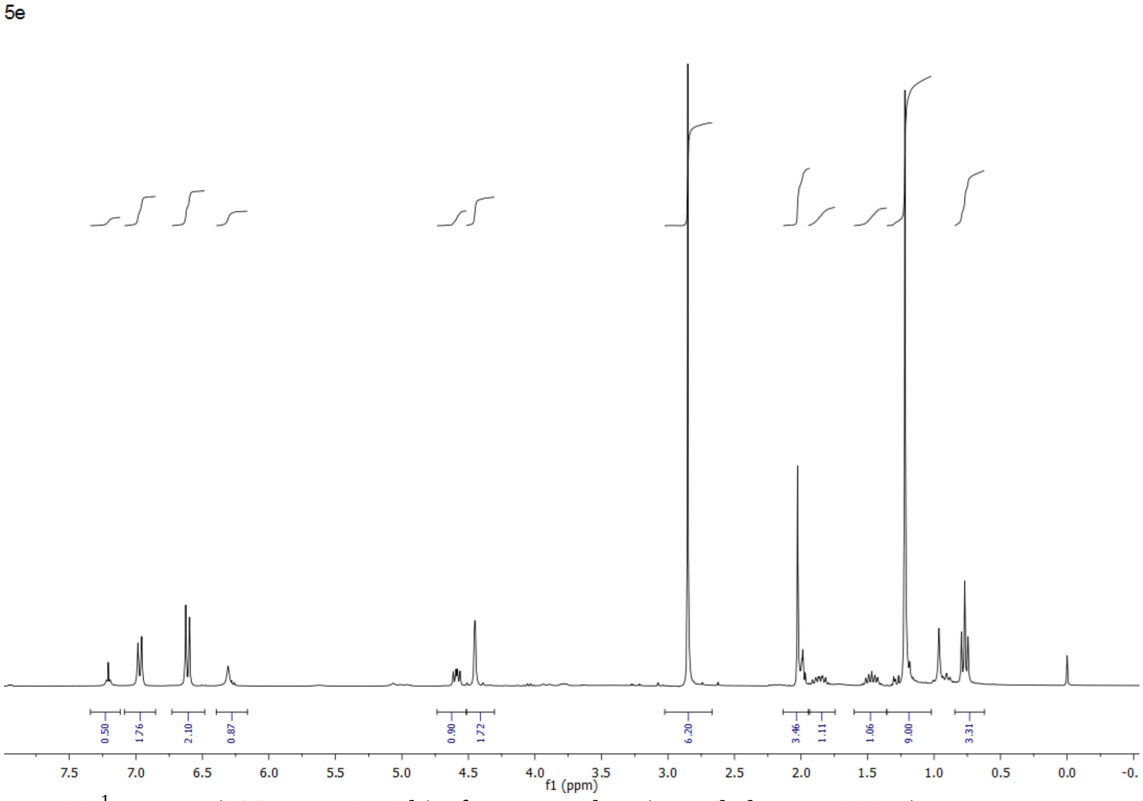

Figure S9. ^{1}H NMR (300 MHz, $CDCl_3$) of compound **5e** (recorded at T = 293 K).

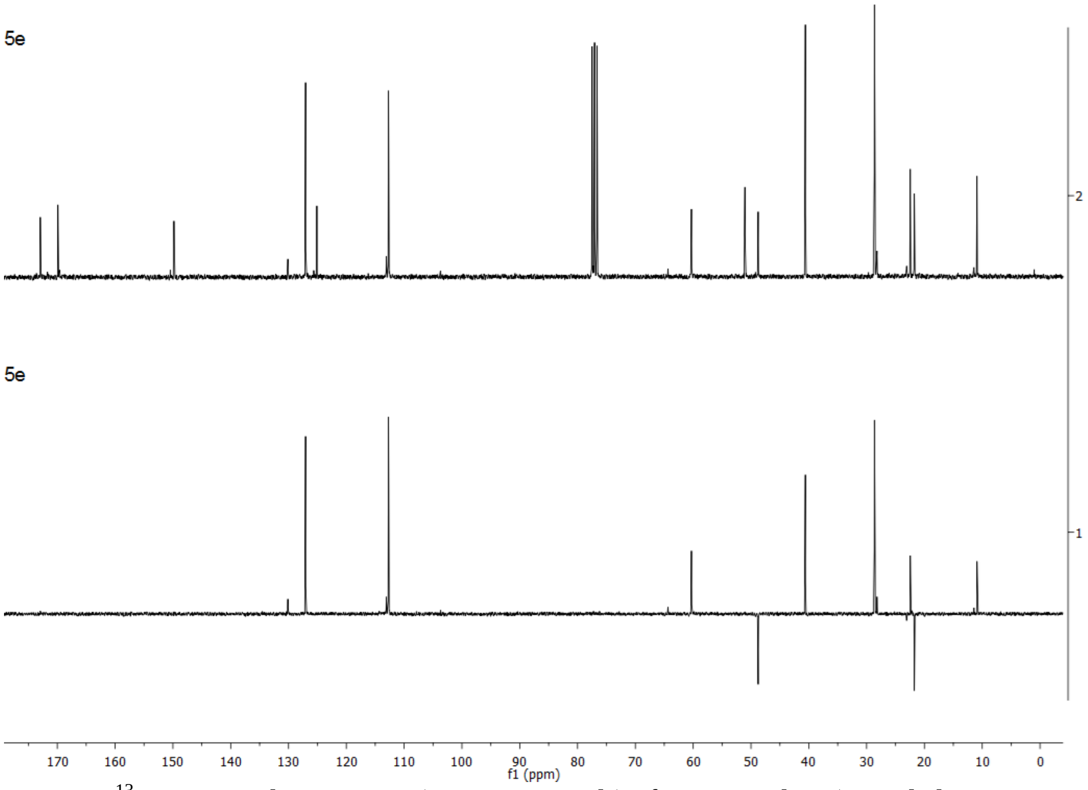

Figure S10. ^{13}C NMR and 135 DEPT (75 MHz, $CDCl_3$) of compound **5e** (recorded at T = 293 K).

2.6 2-(9-Anthryl)-*N*-(*tert*-butyl)-2-(*N*-methylacetamido)acetamide (5f)

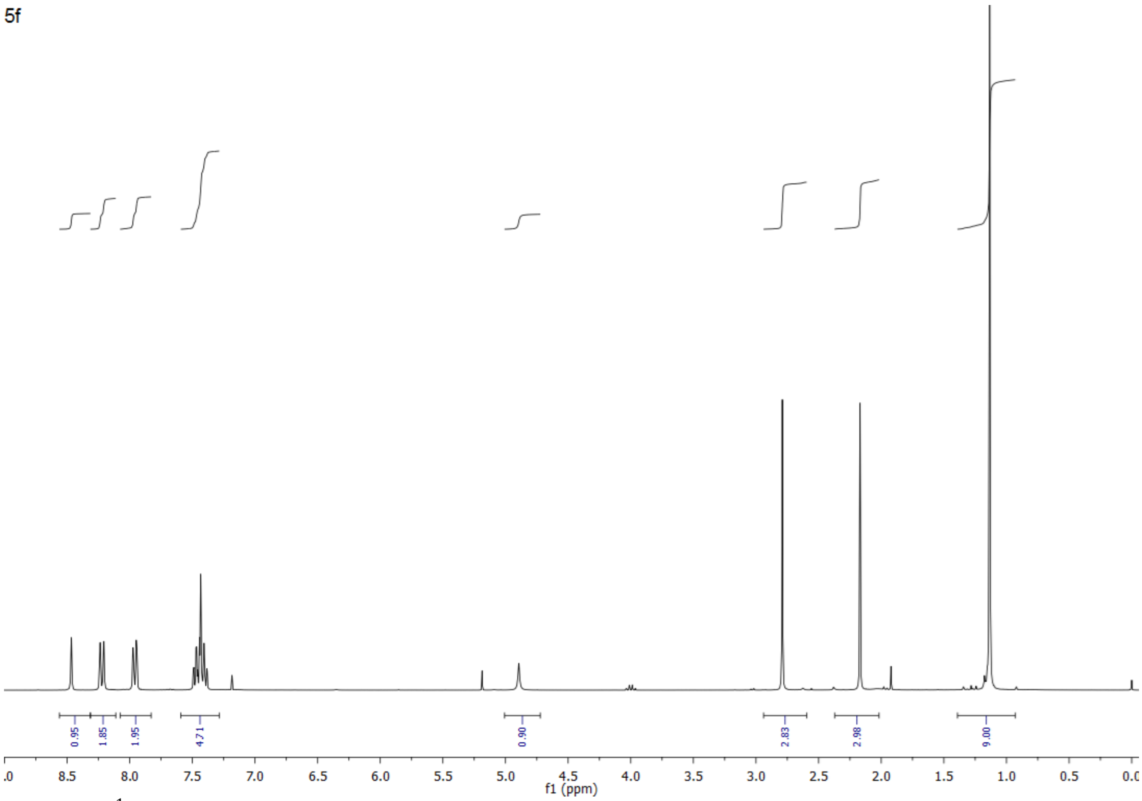

Figure S11. ^{1}H NMR (300 MHz, $CDCl_3$) of compound **5f** (recorded at T = 293 K).

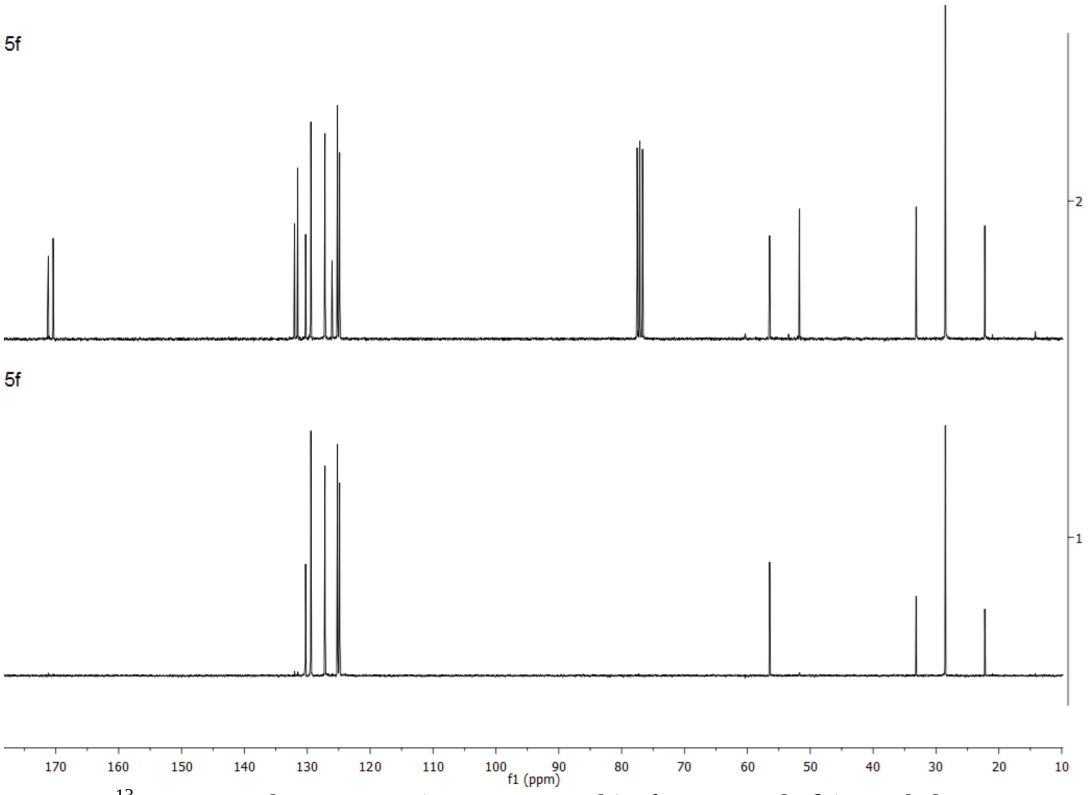

Figure S12. ^{13}C NMR and 135 DEPT (75 MHz, $CDCl_3$) of compound **5f** (recorded at T = 293 K).

2.7 *N*-(*tert*-Butyl)-2-(4-(dimethylamino)phenyl)-2-(*N*-(1-naphthylmethyl)acetamido) acetamide (5g)

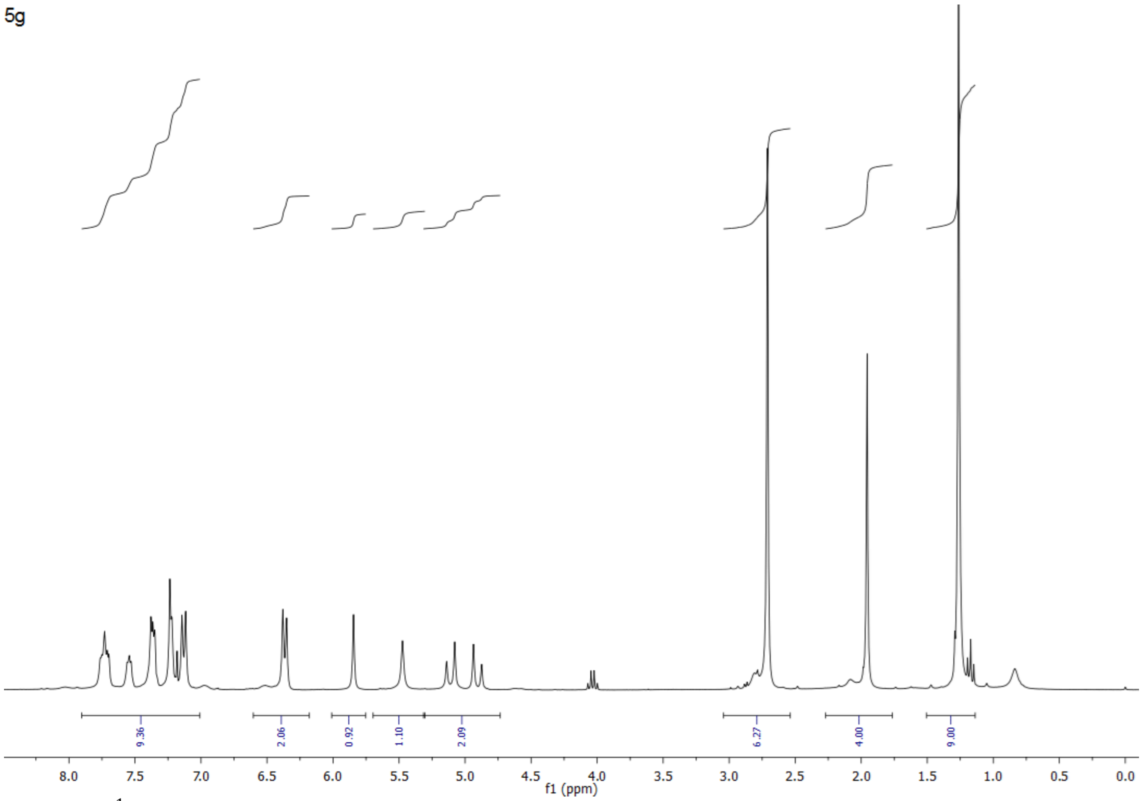

Figure S13. ^{1}H NMR (300 MHz, $CDCl_3$) of compound **5g** (recorded at T = 293 K).

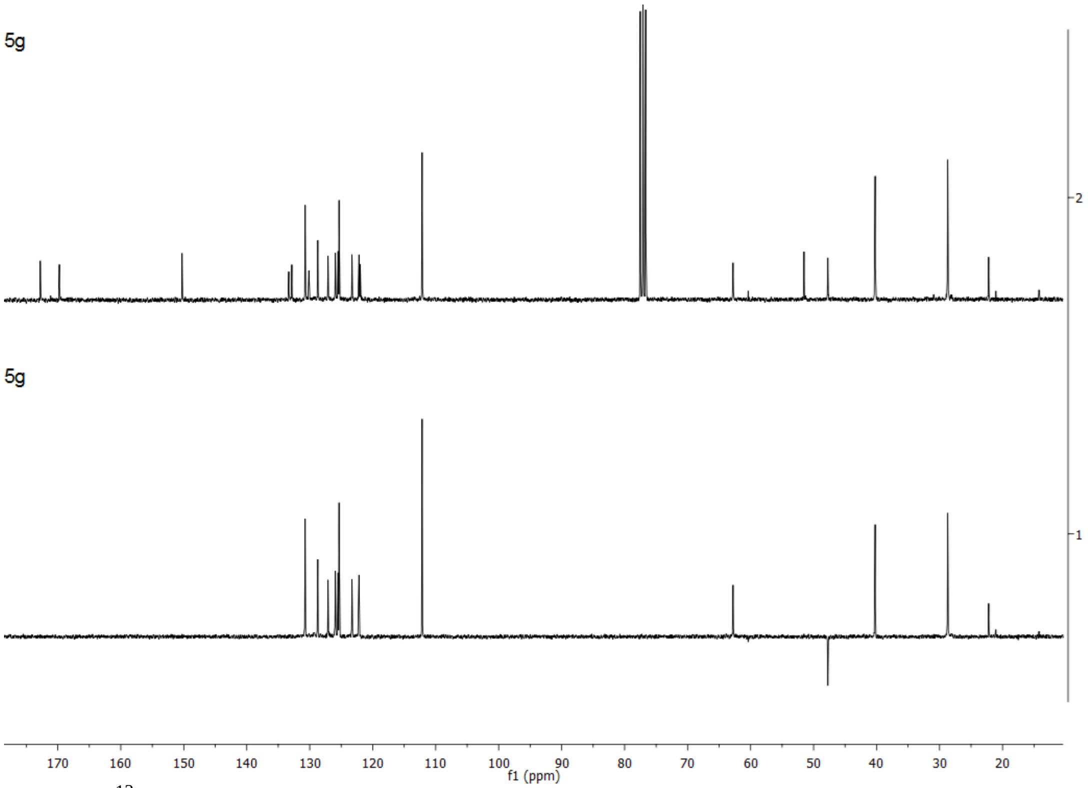

Figure S14. ^{13}C NMR and 135 DEPT (75 MHz, $CDCl_3$) of compound **5g** (recorded at T = 293 K).

2.8 *N*-*tert*-Butyl-2-(*N*-(4-(dimethylamino)benzyl)acetamido)-2-(1-naphthyl)acetamide (5h)

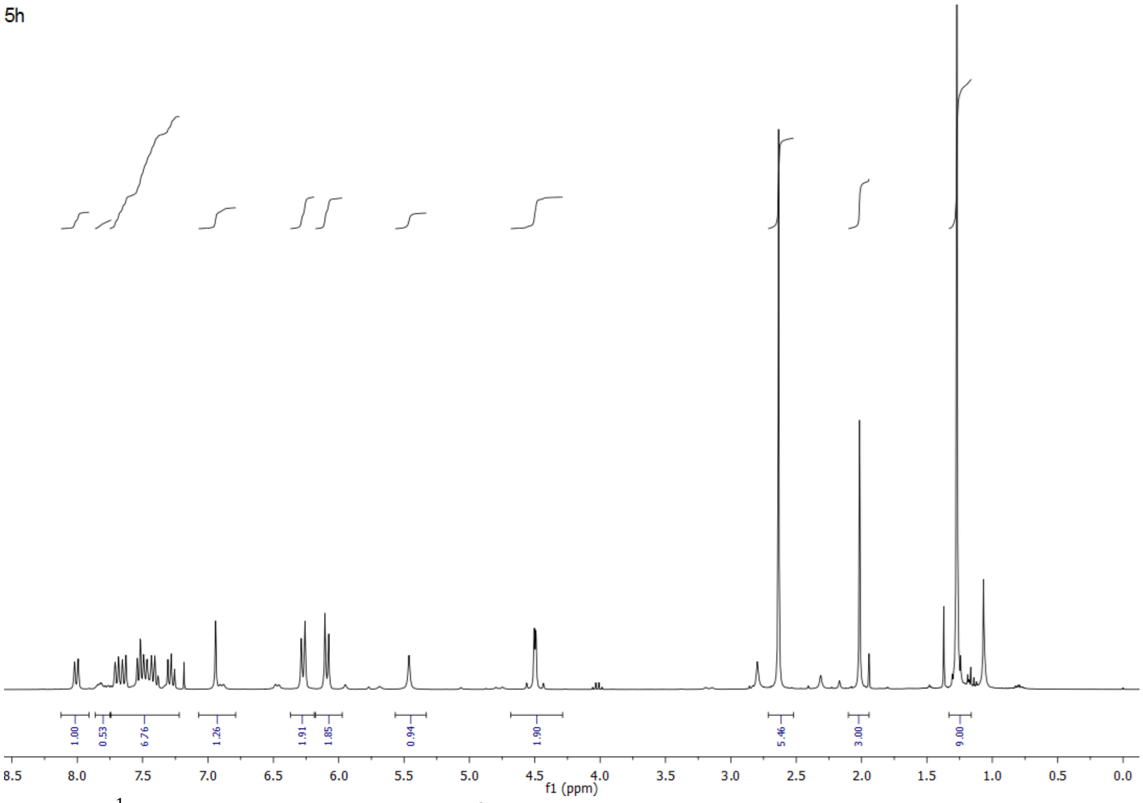

Figure S15. ^{1}H NMR (300 MHz, $CDCl_3$) of compound **5h** (recorded at T = 293 K).

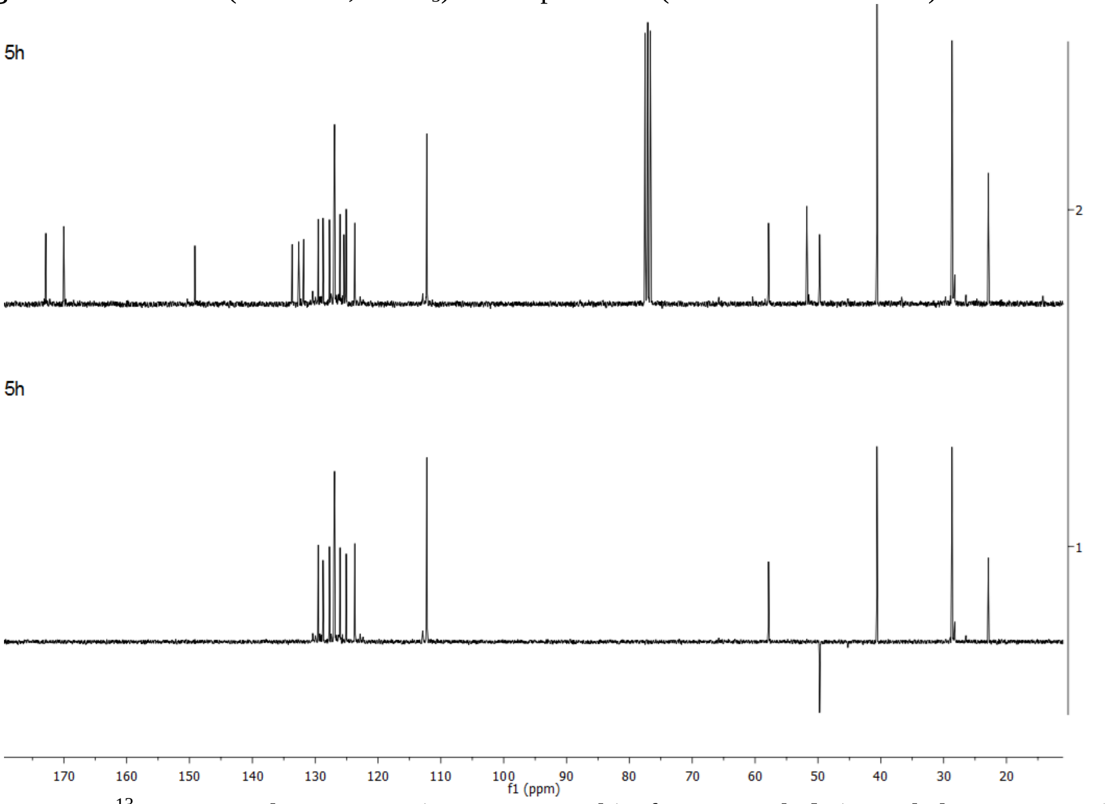

Figure S16. ^{13}C NMR and 135 DEPT (75 MHz, $CDCl_3$) of compound **5h** (recorded at T = 293 K).

2.9 *N*-*tert*-Butyl-2-(4-(dimethylamino)phenyl)-2-(*N*-(1-pyrenylmethyl)acetamido) acetamide (5i)

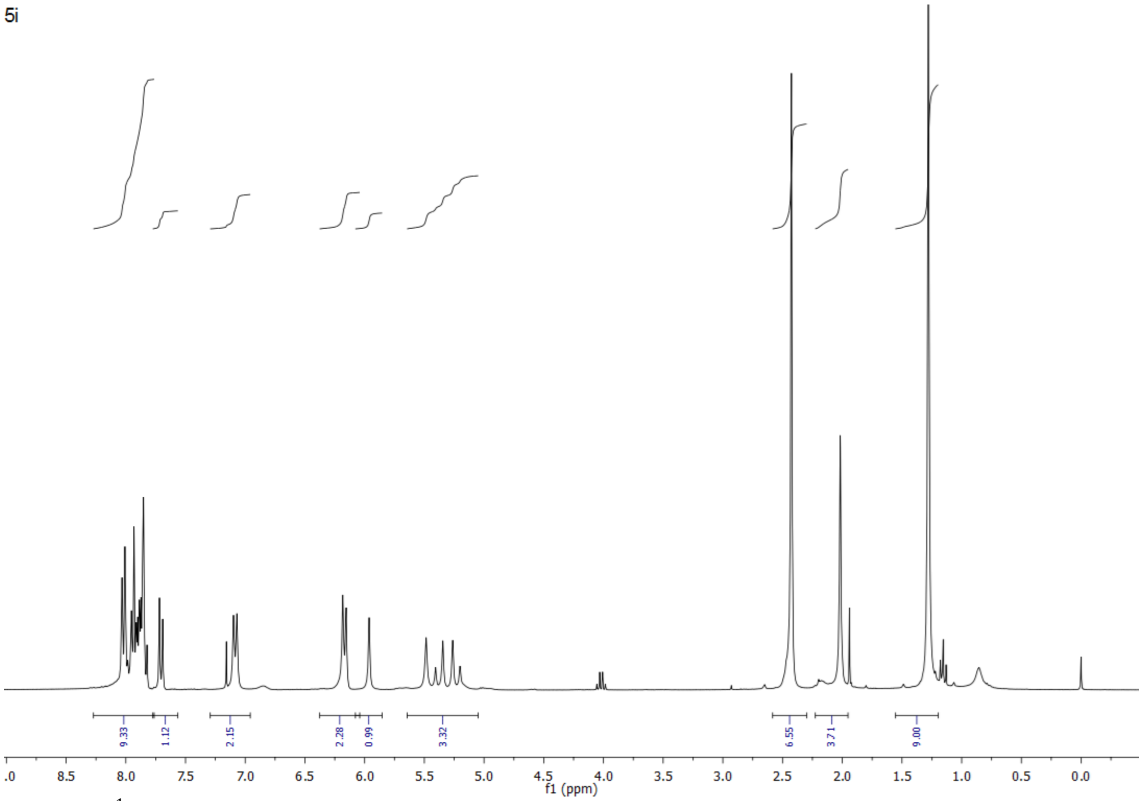

Figure S17. ^{1}H NMR (300 MHz, $CDCl_3$) of compound **5i** (recorded at *T* = 293 K).

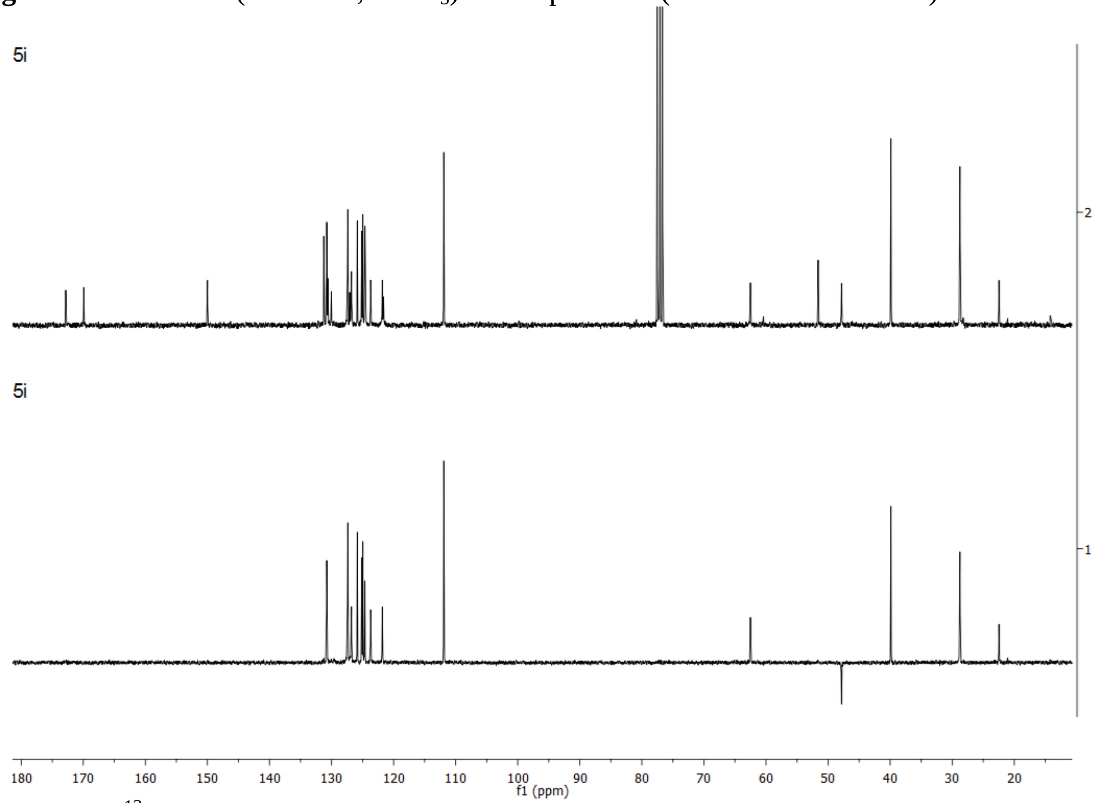

Figure S18. ^{13}C NMR and 135 DEPT (75 MHz, $CDCl_3$) of compound **5i** (recorded at *T* = 293 K).

2.10 *N*-(*tert*-Butyl)-2-(*N*-(4-(dimethylamino)benzyl)acetamido)-2-(1-pyrenyl)acetamide (5j)

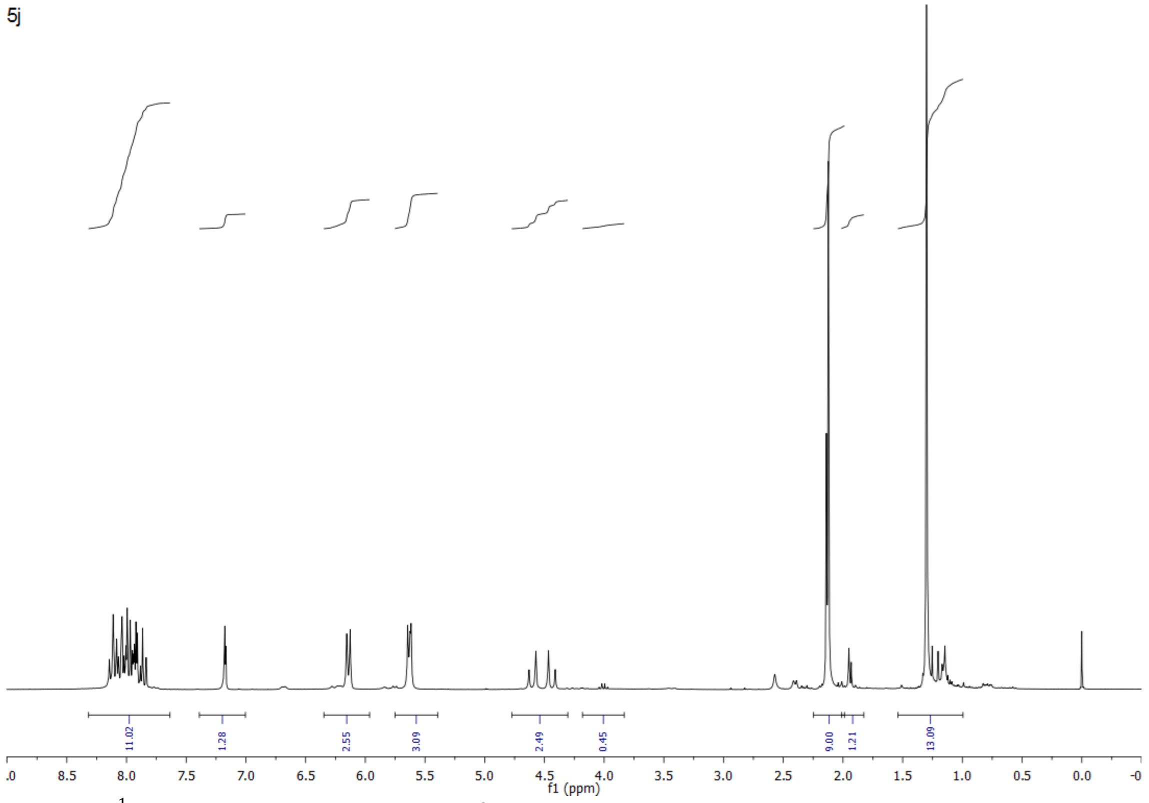

Figure S19. ^{1}H NMR (300 MHz, $CDCl_3$) of compound **5j** (recorded at T = 293 K).

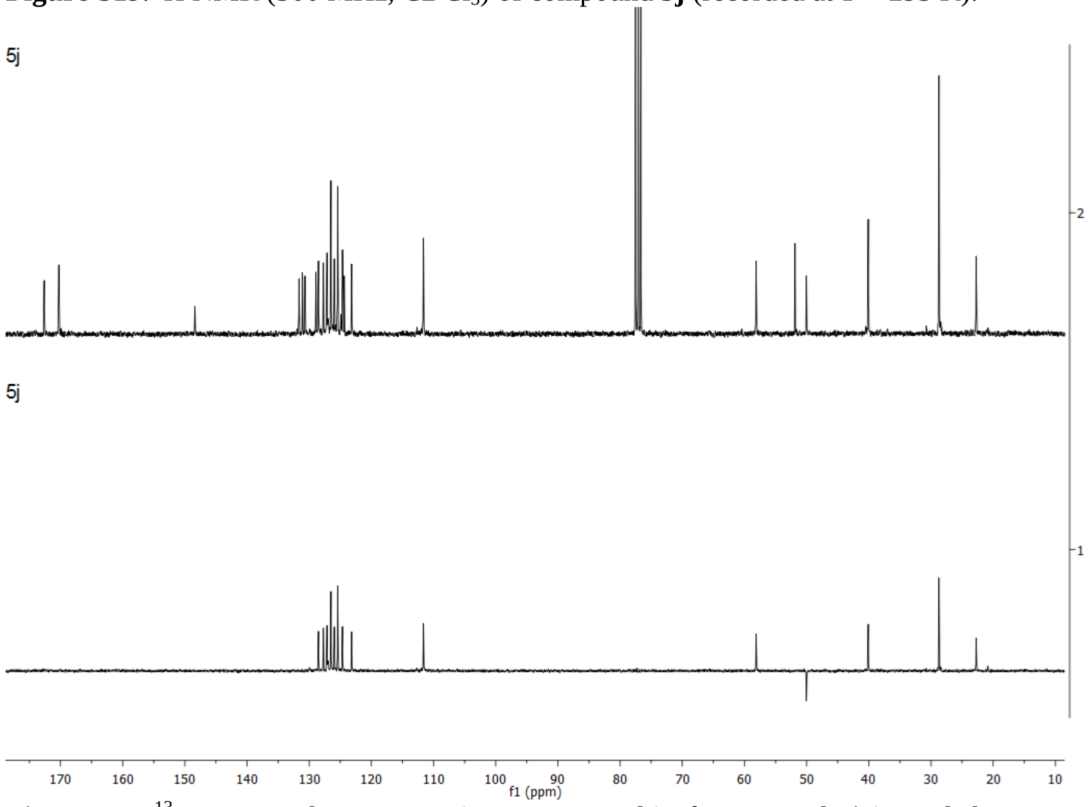

Figure S20. ^{13}C NMR and 135 DEPT (75 MHz, $CDCl_3$) of compound **5j** (recorded at T = 293 K).

3. Absorption and Emission Spectra of Compounds 5

3.1 2-(*N*-(9-Anthrylmethyl)acetamido)-*N*-(*tert*-butyl)-2-(4-(dimethylamino)phenyl) acetamide (5a)

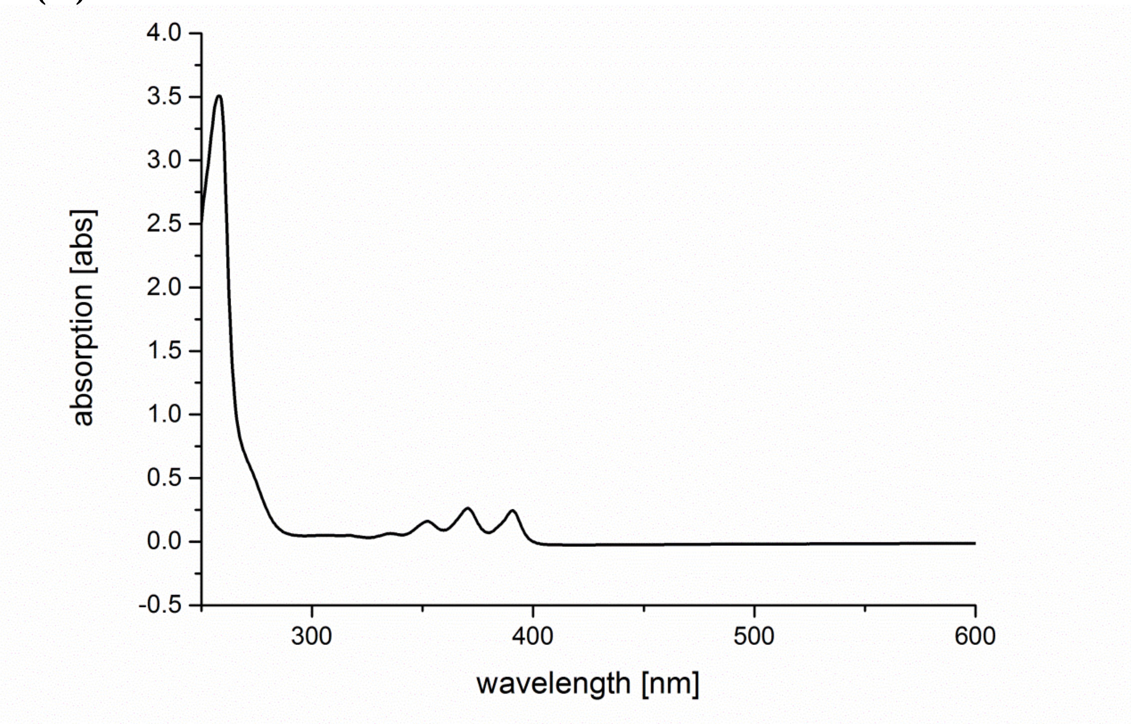

Figure S21. UV/Vis spectrum of compound **5a** (recorded in CH_2Cl_2 (chromasolv), *c*(**5a**) = 10^{-3} M, *T* = 293 K).

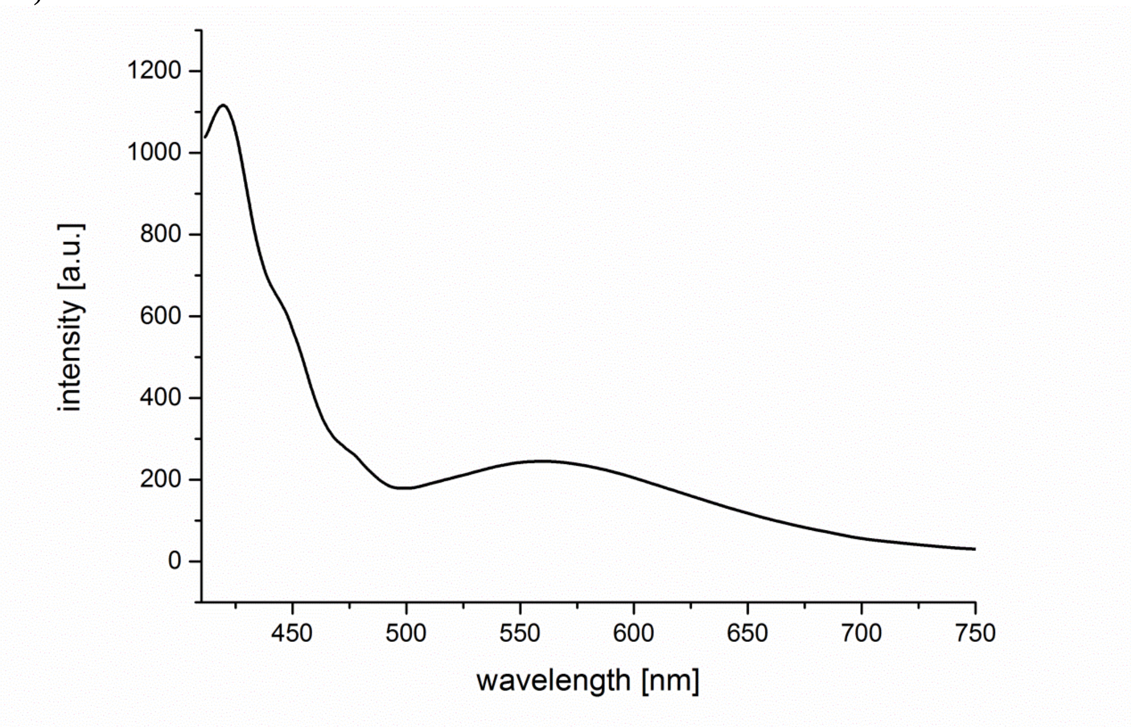

Figure S22. Emission spectrum of compound **5a** (recorded in CH_2Cl_2 (chromasolv), *c*(**5a**) = 10^{-5} M, *T* = 293 K, λ_{exc} = 391 nm).

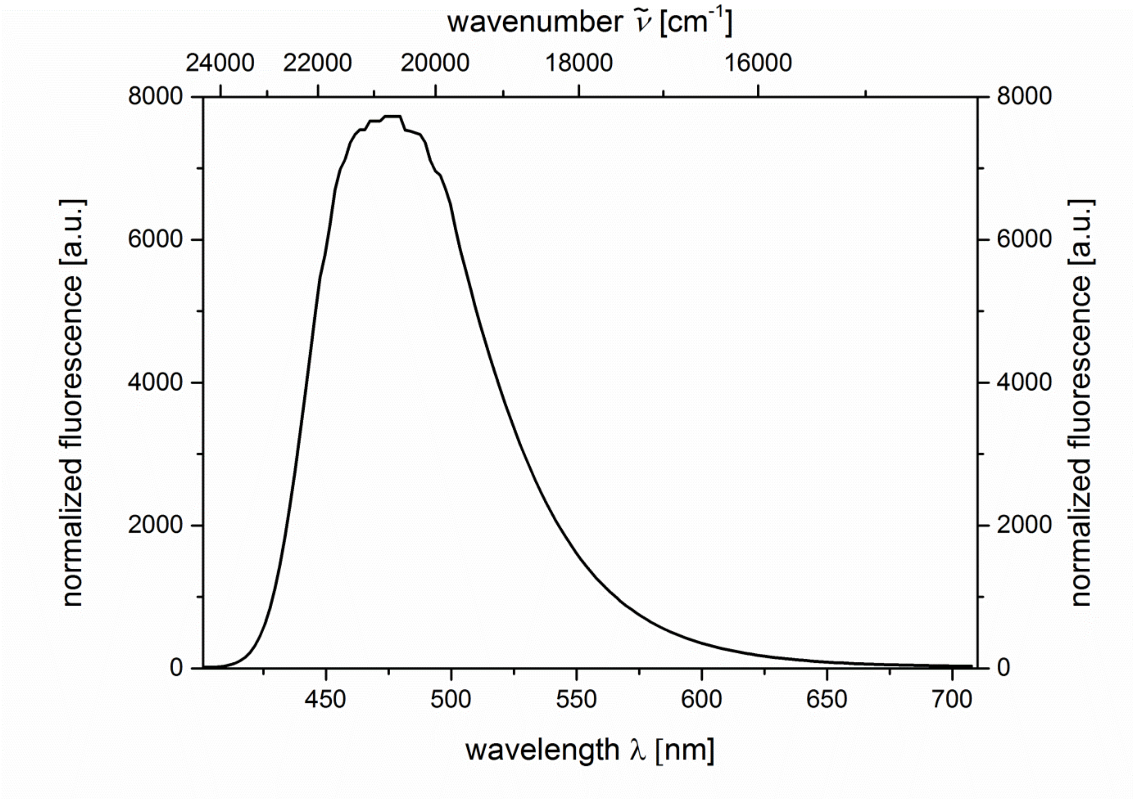

Figure S23. Solid state emission spectrum of dyad **5a** (λ_{exc} = 391 nm, T = 298 K).

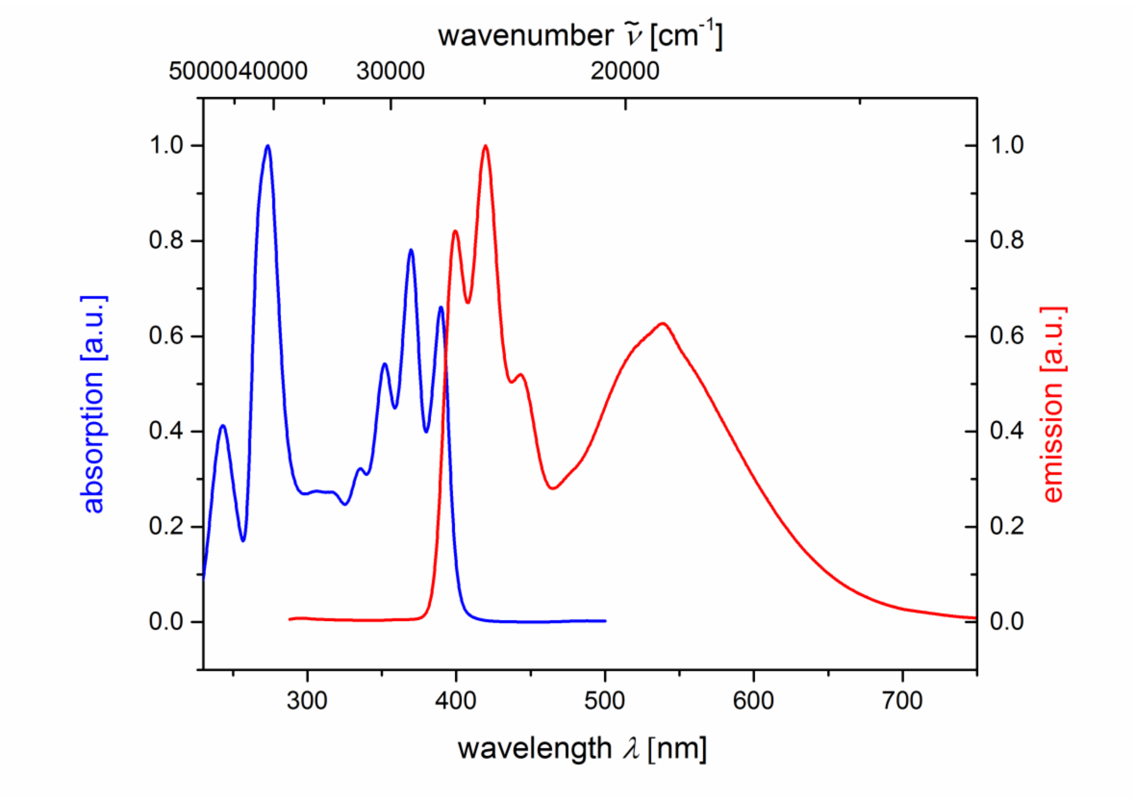

Figure S24. Normalized excitation (absorption) (blue lines) and emission (red lines) spectra of compound **5a** (recorded in CH_2Cl_2 (chromasolv), T = 298 K, λ_{exc} = 268 nm).

3.2 2-(9-Anthryl)-*N*-(*tert*-butyl)-2-(*N*-(4-(dimethylamino)benzyl)acetamido)acetamide (5b)

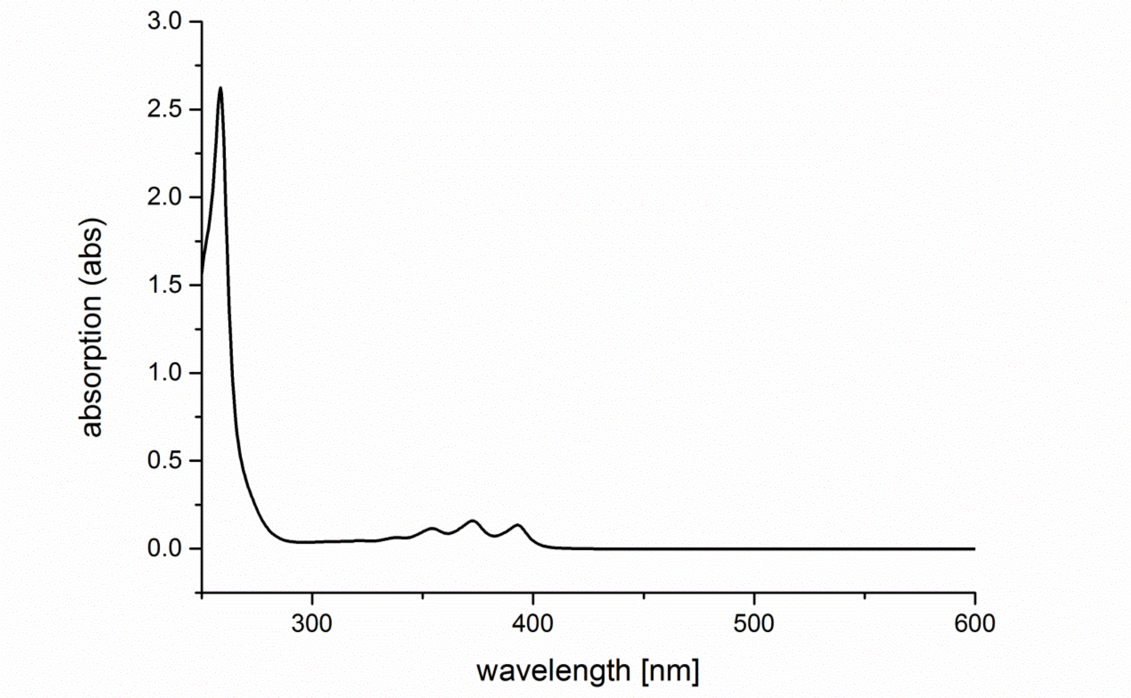

Figure S25. UV/Vis spectrum of compound **5b** (recorded in CH_2Cl_2 (chromasolv), *c*(**5b**) = 10^{-3} M, *T* = 293 K).

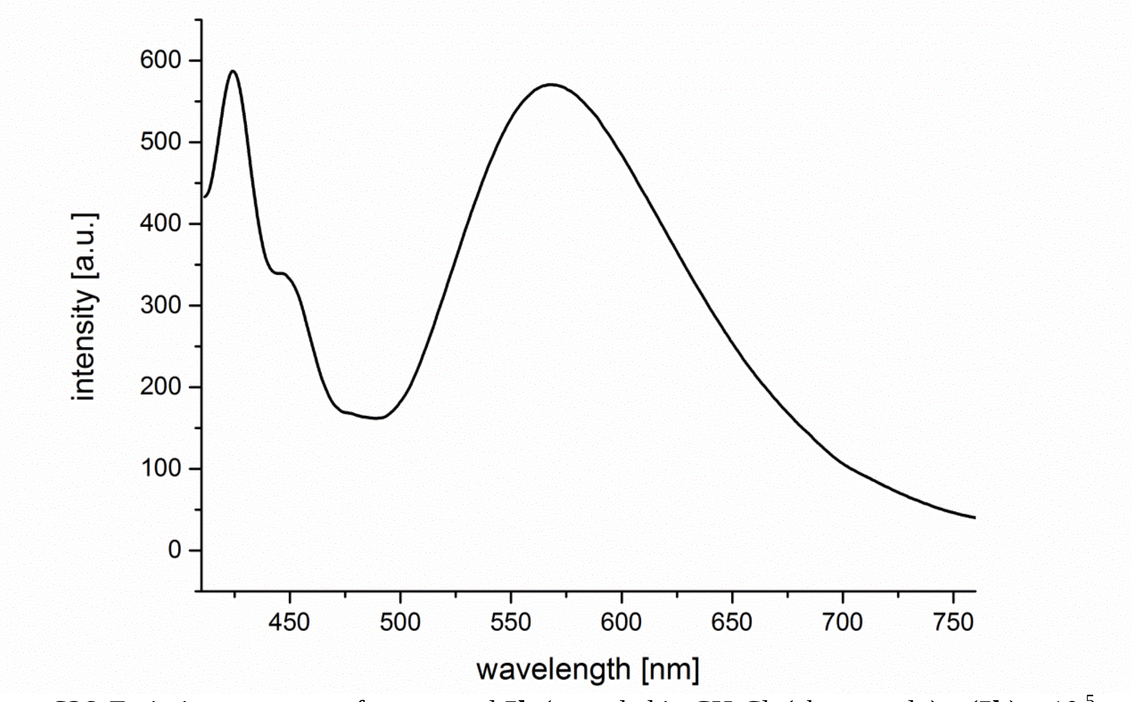

Figure S26. Emission spectrum of compound **5b** (recorded in CH_2Cl_2 (chromasolv), *c*(**5b**) = 10^{-5} M, *T* = 293 K, λ_{exc} = 391 nm).

3.3 2-(*N*-(9-Anthrylmethyl)acetamido)-*N*-*tert*-butylbutanamide (5c)

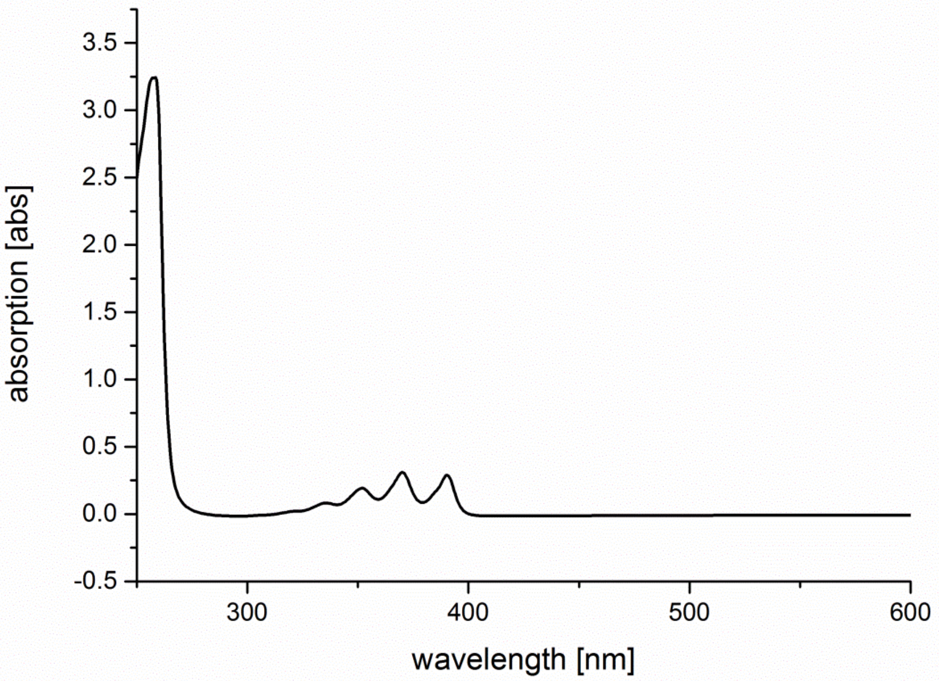

Figure S27. UV/Vis spectrum of compound **5c** (recorded in CH_2Cl_2 (chromasolv), *c*(**5c**) = 10^{-3} M, *T* = 293 K).

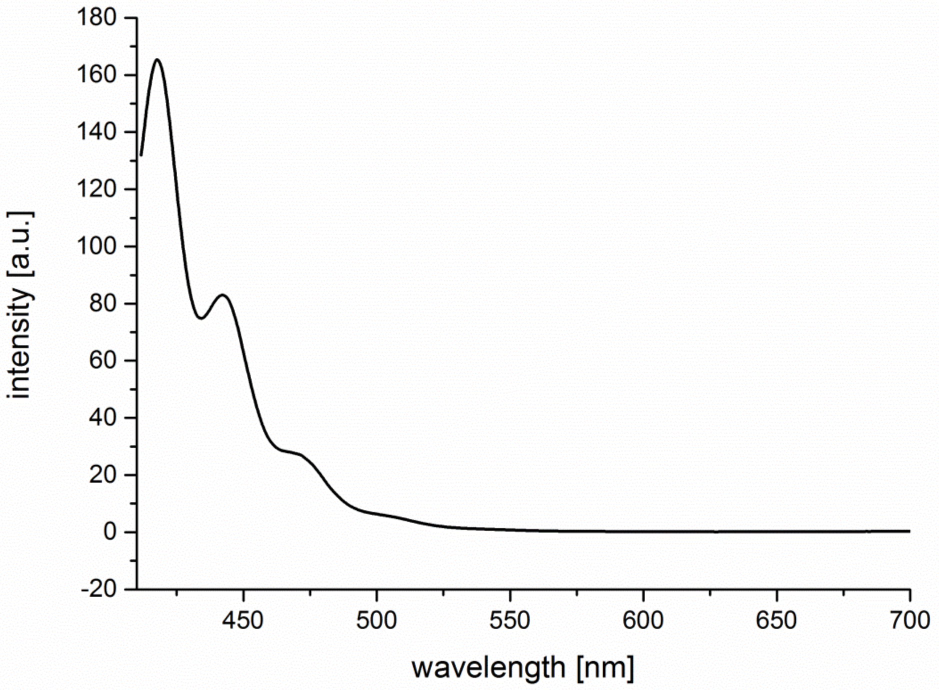

Figure S28. Emission spectrum of compound **5c** (recorded in CH_2Cl_2 (chromasolv), *c*(**5c**) = 10^{-5} M, *T* = 293 K, λ_{exc} = 391 nm).

3.4 *N-tert*-Butyl-2-(4-(dimethylamino)phenyl)-2-(*N*-methylacetamido)acetamide (5d)

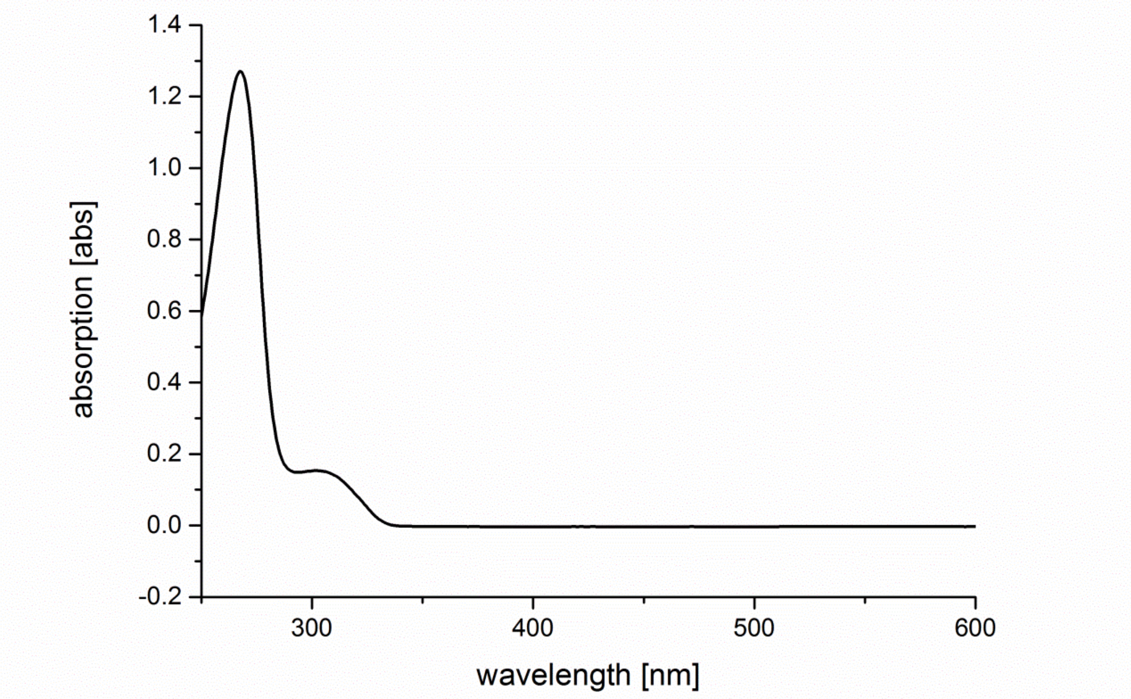

Figure S29. UV/Vis spectrum of compound **5d** (recorded in CH_2Cl_2 (chromasolv), *c*(**5d**) = 10^{-3} M, *T* = 293 K).

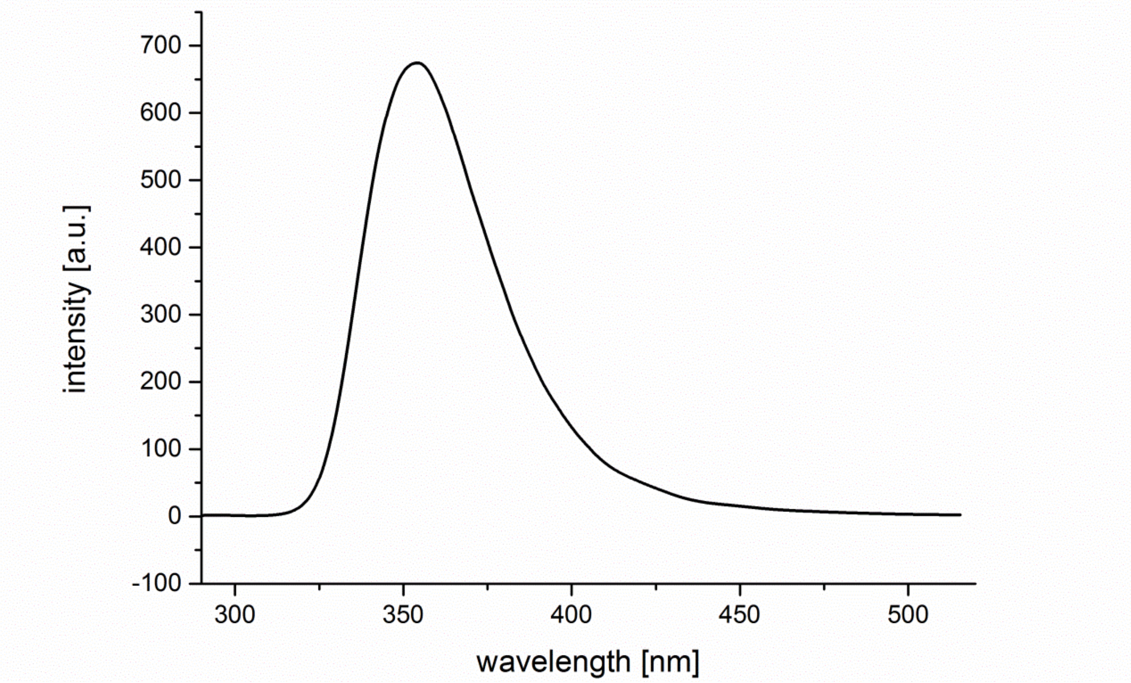

Figure S30. Emission spectrum of compound **5d** (recorded in CH_2Cl_2 (chromasolv), *c*(**5d**) = 10^{-5} M, *T* = 293 K, λ_{exc} = 268 nm).

3.5 *N-tert*-Butyl-2-(*N*-(4-(dimethylamino)benzyl)acetamido)butanamide (5e)

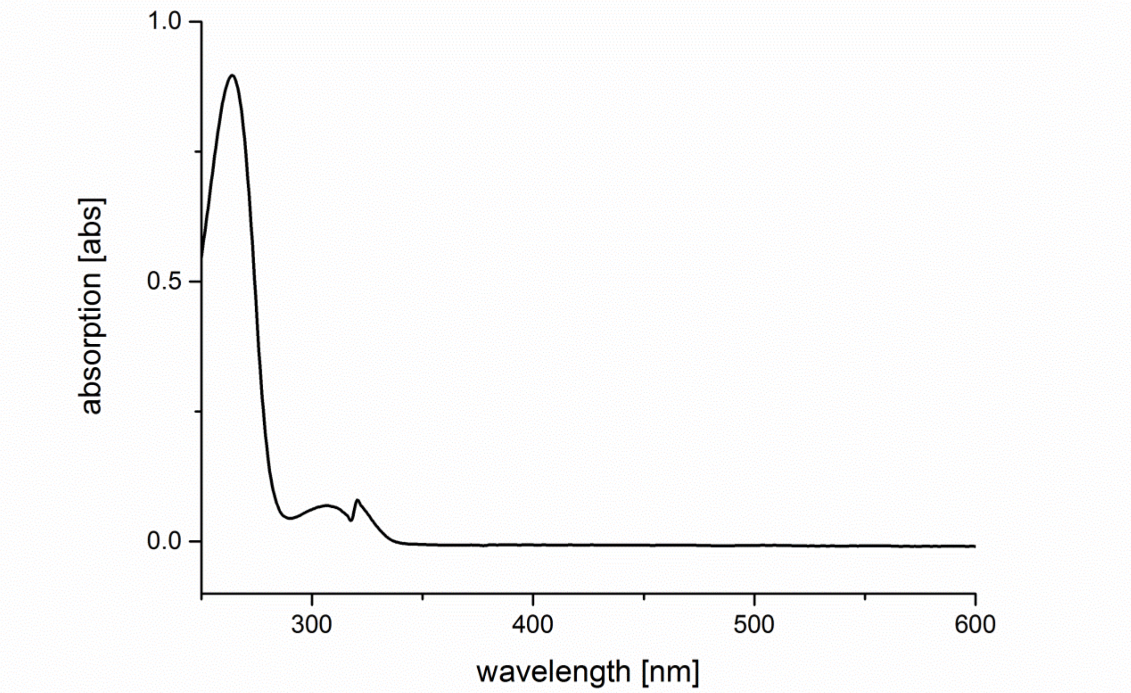

Figure S31. UV/Vis spectrum of compound **5e** (recorded in CH_2Cl_2 (chromasolv), *c*(**5e**) = 10^{-3} M, *T* = 293 K).

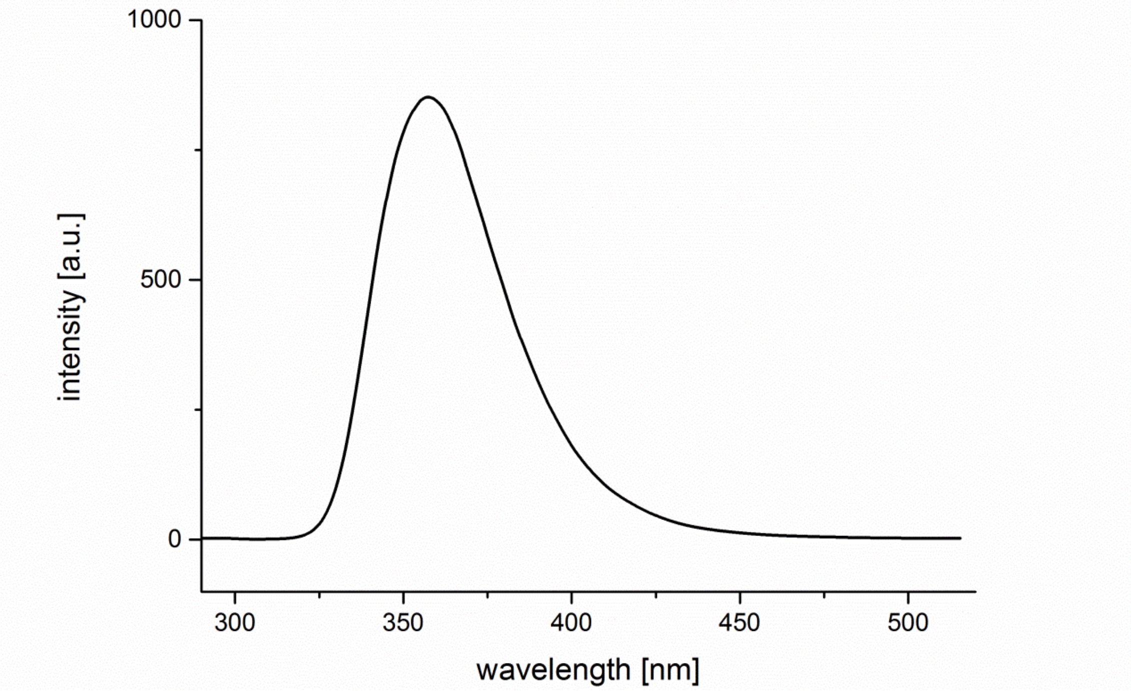

Figure S32. Emission spectrum of compound **5e** (recorded in CH_2Cl_2 (chromasolv), *c*(**5e**) = 10^{-5} M, *T* = 293 K, λ_{exc} = 268 nm).

3.7 2-(9-Anthryl)-*N*-(*tert*-butyl)-2-(*N*-methylacetamido)acetamide (5f)

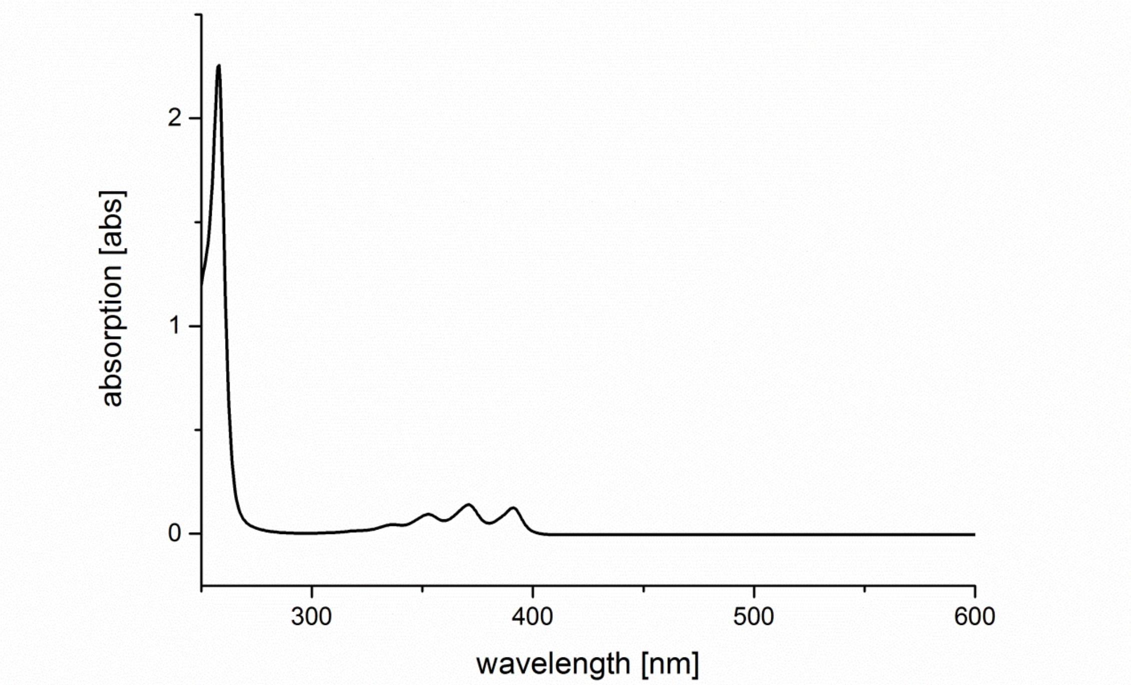

Figure S33. UV/Vis spectrum of compound **5f** (recorded in CH_2Cl_2 (chromasolv), *c*(**5f**) = 10^{-3} M, *T* = 293 K).

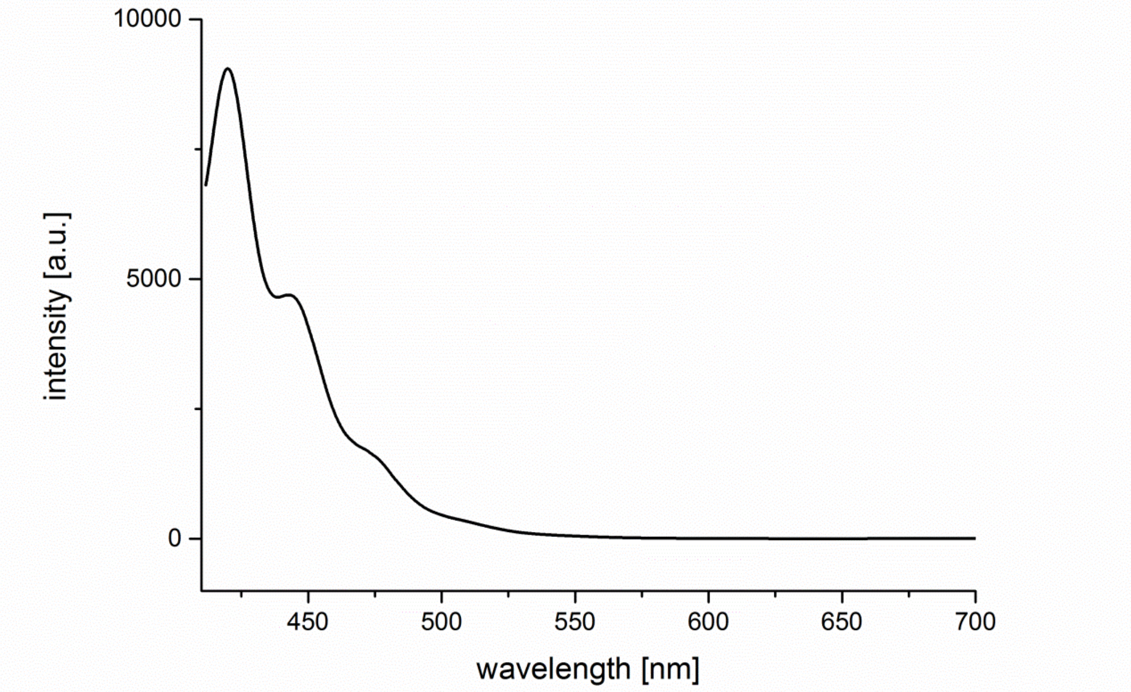

Figure S34. Emission spectrum of compound **5f** (recorded in CH_2Cl_2 (chromasolv), *c*(**5f**) = 10^{-5} M, *T* = 293 K, λ_{exc} = 391 nm).

3.8 *N*-(*tert*-Butyl)-2-(4-(dimethylamino)phenyl)-2-(*N*-(1-naphthylmethyl)acetamido) acetamide (5g)

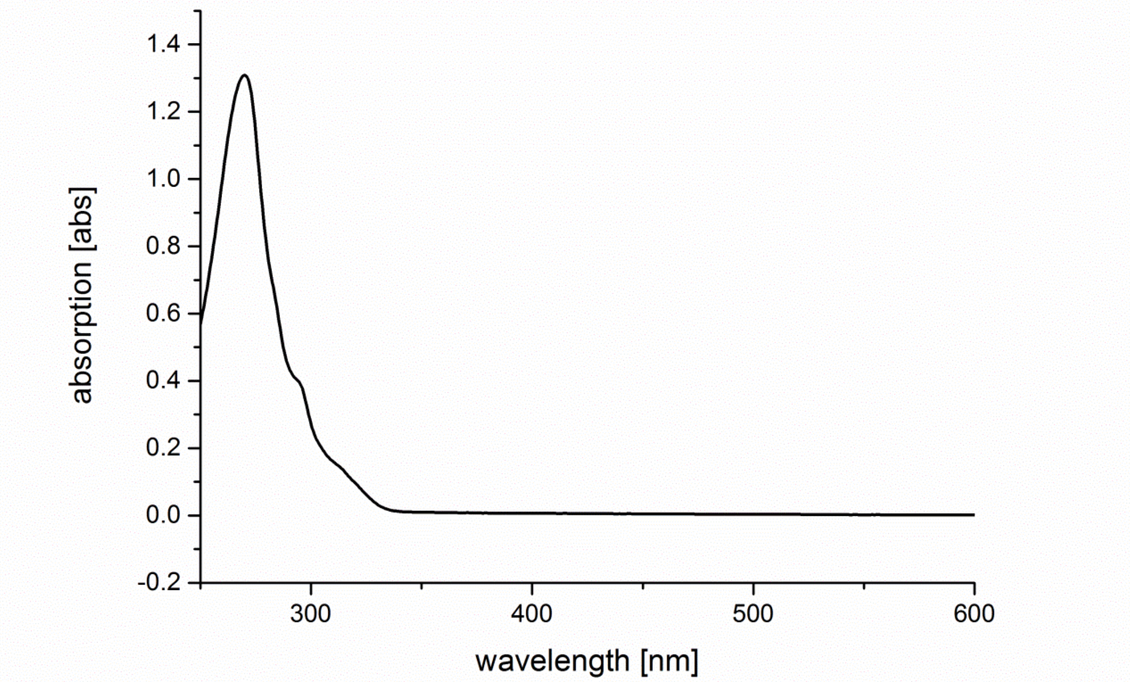

Figure S35. UV/Vis spectrum of compound **5g** (recorded in CH_2Cl_2 (chromasolv), *c*(**5g**) = 10^{-3} M, *T* = 293 K).

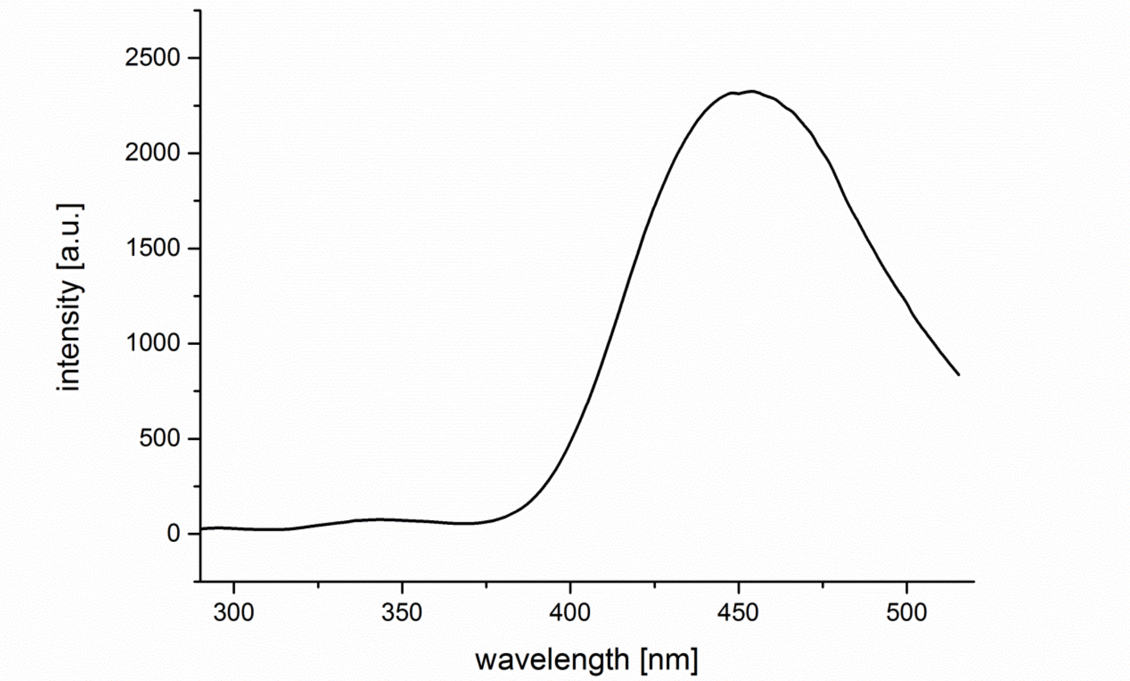

Figure S36. Emission spectrum of compound **5g** (recorded in CH_2Cl_2 (chromasolv), *c*(**5g**) = 10^{-5} M, *T* = 293 K, λ_{exc} = 268 nm).

3.10 *N-tert*-Butyl-2-(*N*-(4-(dimethylamino)benzyl)acetamido)-2-(1-naphthyl)acetamide (5h)

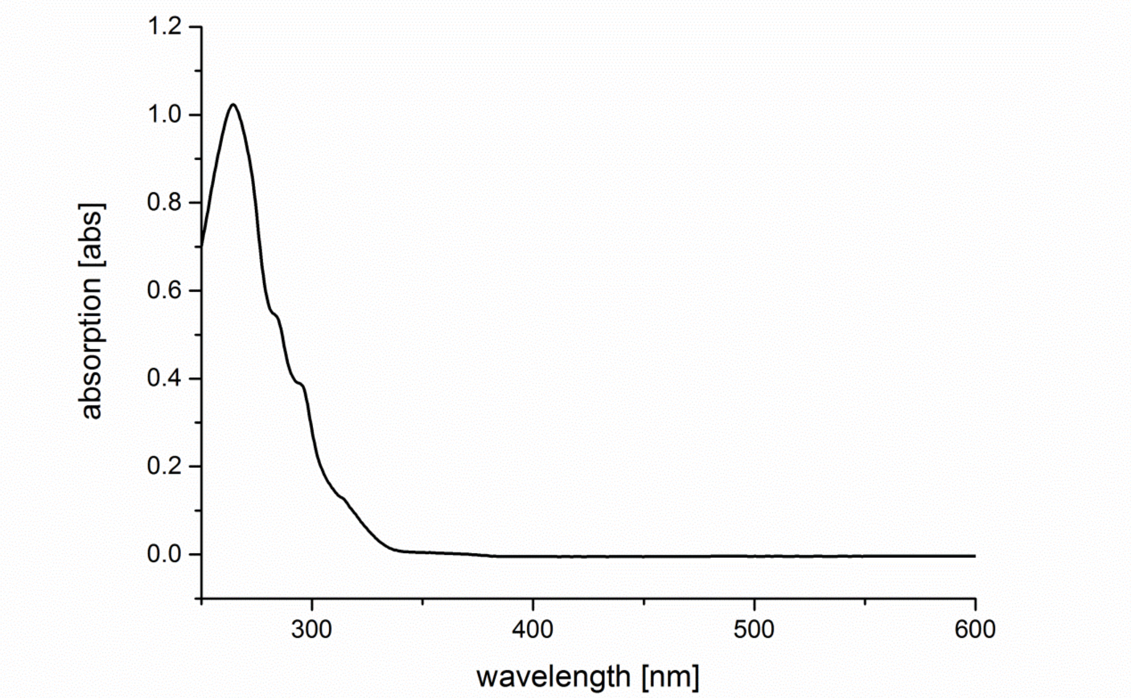

Figure S37. UV/Vis spectrum of compound **5h** (recorded in CH_2Cl_2 (chromasolv), c(**5h**) = 10^{-3} M, T = 293 K).

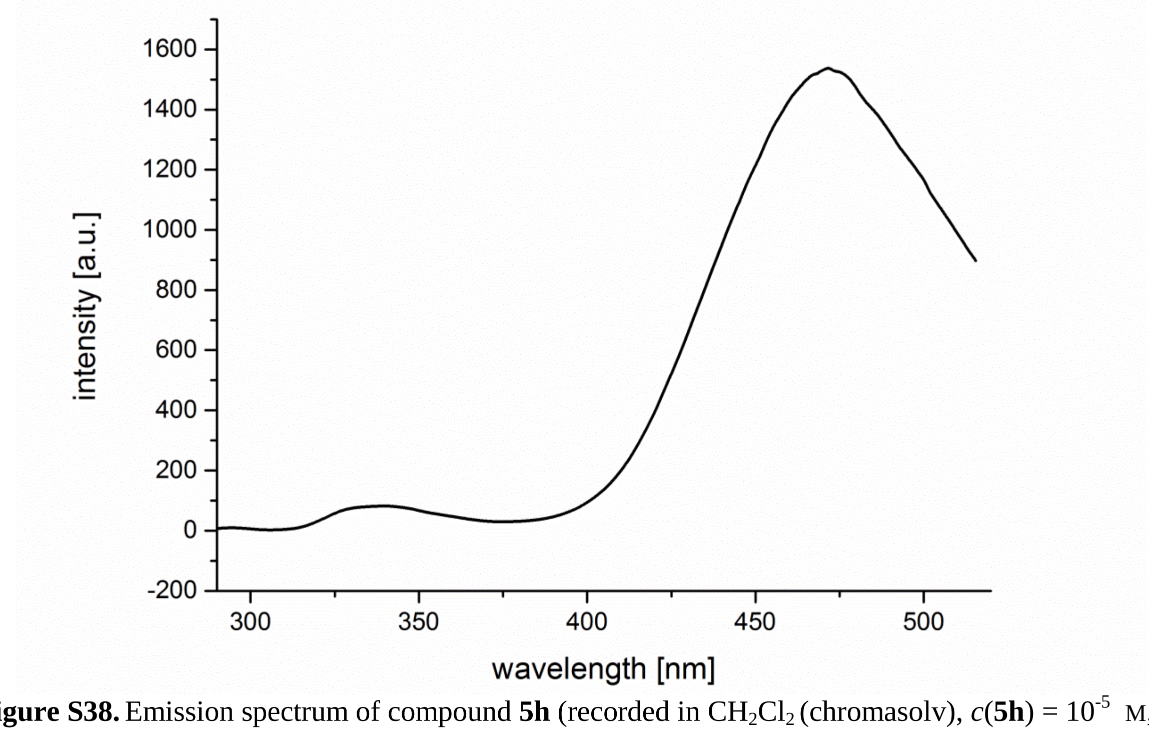

Figure S38. Emission spectrum of compound **5h** (recorded in CH_2Cl_2 (chromasolv), c(**5h**) = 10^{-5} M, T = 293 K, λ_{exc} = 268 nm).

3.11 *N-tert*-Butyl-2-(4-(dimethylamino)phenyl)-2-(*N*-(1-pyrenylmethyl)acetamido) acetamide (5i)

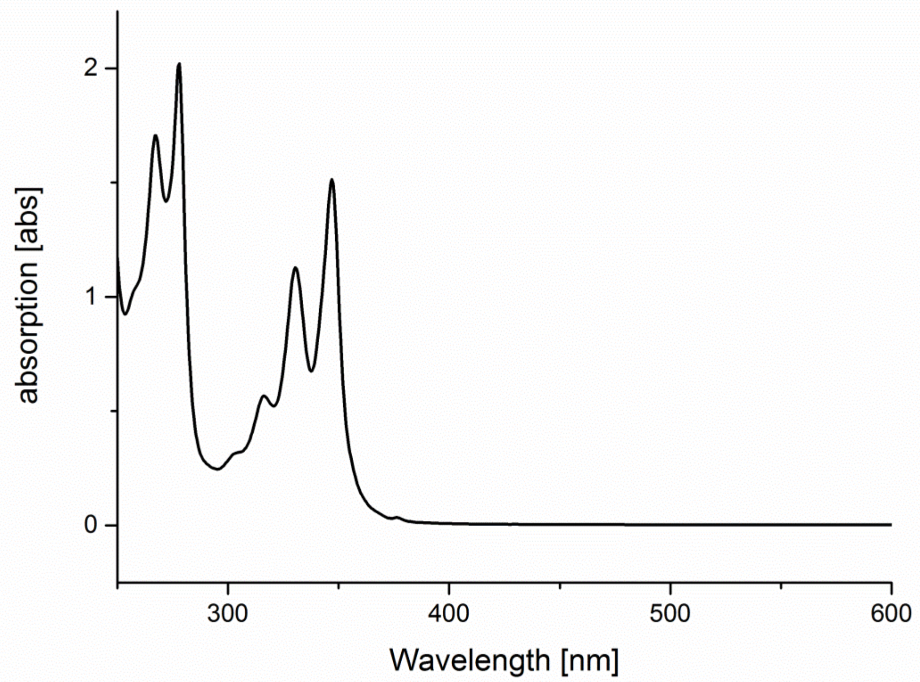

Figure S39. UV/Vis spectrum of compound **5i** (recorded in CH_2Cl_2 (chromasolv), c(**5i**) = 10^{-3} M, T = 293 K).

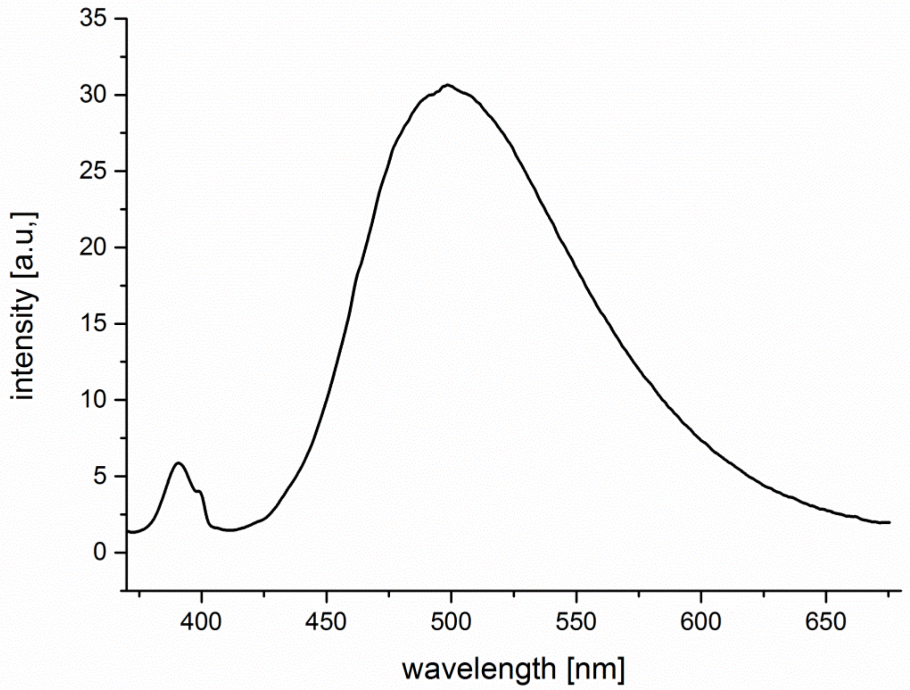

Figure S40. Emission spectrum of compound **5i** (recorded in CH_2Cl_2 (chromasolv), c(**5i**) = 10^{-5} M, T = 293 K, λ_{exc} = 348 nm).

3.12 *N*-(*tert*-Butyl)-2-(*N*-(4-(dimethylamino)benzyl)acetamido)-2-(1-pyrenyl)acetamide (5j)

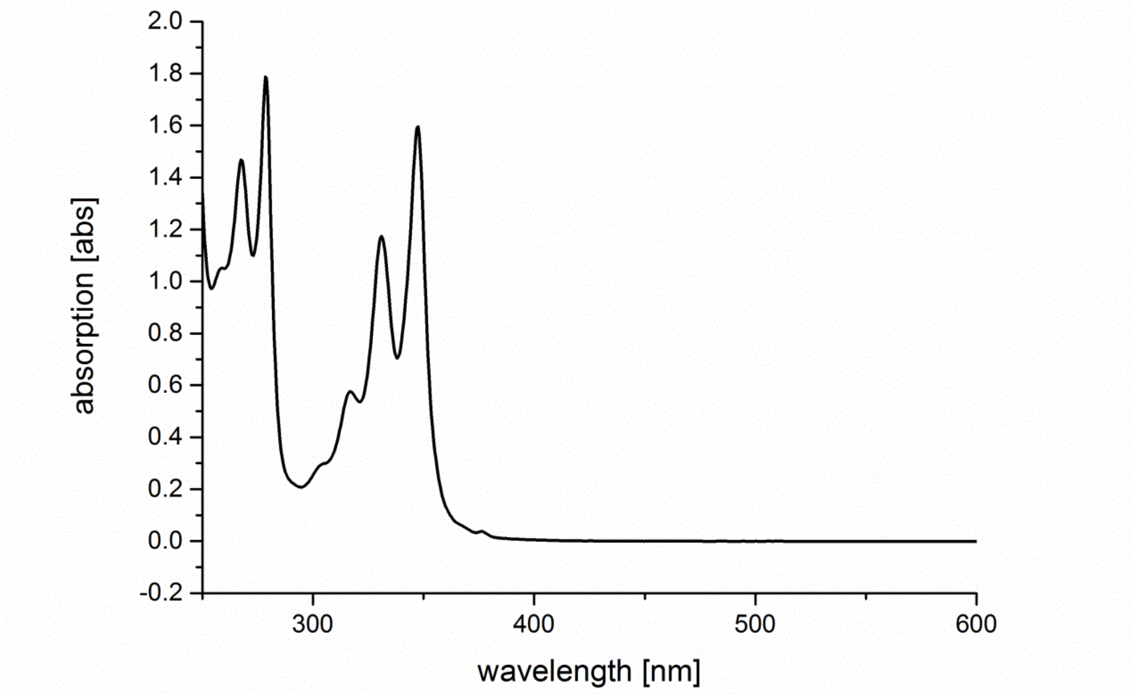

Figure S41. UV/Vis spectrum of compound **5j** (recorded in CH_2Cl_2 (chromasolv), *c*(**5j**) = 10^{-3} M, *T* = 293 K).

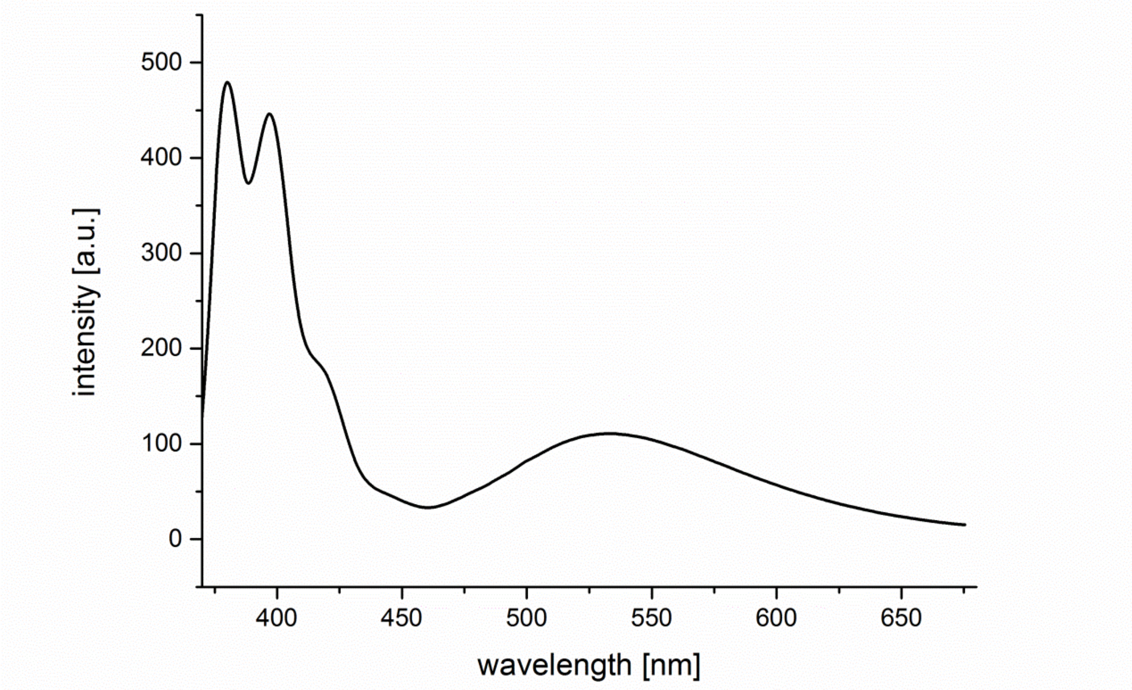

Figure S42. Emission spectrum of compound **5j** (recorded in CH_2Cl_2 (chromasolv), *c*(**5j**) = 10^{-5} M, *T* = 293 K, λ_{exc} = 348 nm).

4. Lippert-Mataga Analysis of Compound 5a

Table S3. Selected solvent parameters and absorptions, emissions and Stokes shifts.

**Solvent**	**Permittivity**[1] ε_r	**Refractive index**[2] n_D^{20}	**Δf**	**Absorption** λ_{max} [nm] (ε [$Lmol^{-1}cm^{-1}$])	**Emission** $\lambda_{max,em}$ [nm]	**Stokes shift** $\Delta\tilde{\nu}$ [cm^{-1}]
1,4-dioxane	2.25	1.4224	0.025	352 (6400), 370 (9900), 389 (8900)	418, **543**	5773
dichloromethane	8.93	1.4241	0.217	352 (5100), 371 (8300), 391 (7700)	417, 444, **561**	7750
acetone	20.7	1.3587	0.284	351 (6100), 369 (9400), 389 (8900)	418, 442, **560**	7850
acetonitrile	35.94	1.3441	0.305	351 (6200), 369 (9300), 389 (8700)	416, 442, **572**	8532

$$\Delta f = \frac{\varepsilon_r - 1}{2\varepsilon_r + 1} - \frac{n^2 - 1}{2n^2 + 1}$$

Lippert-Mataga equation $$\tilde{\nu}_a - \tilde{\nu}_e = \frac{2\Delta f}{4\pi\varepsilon_0 hca^3}(\mu_E - \mu_G)^2 + const$$

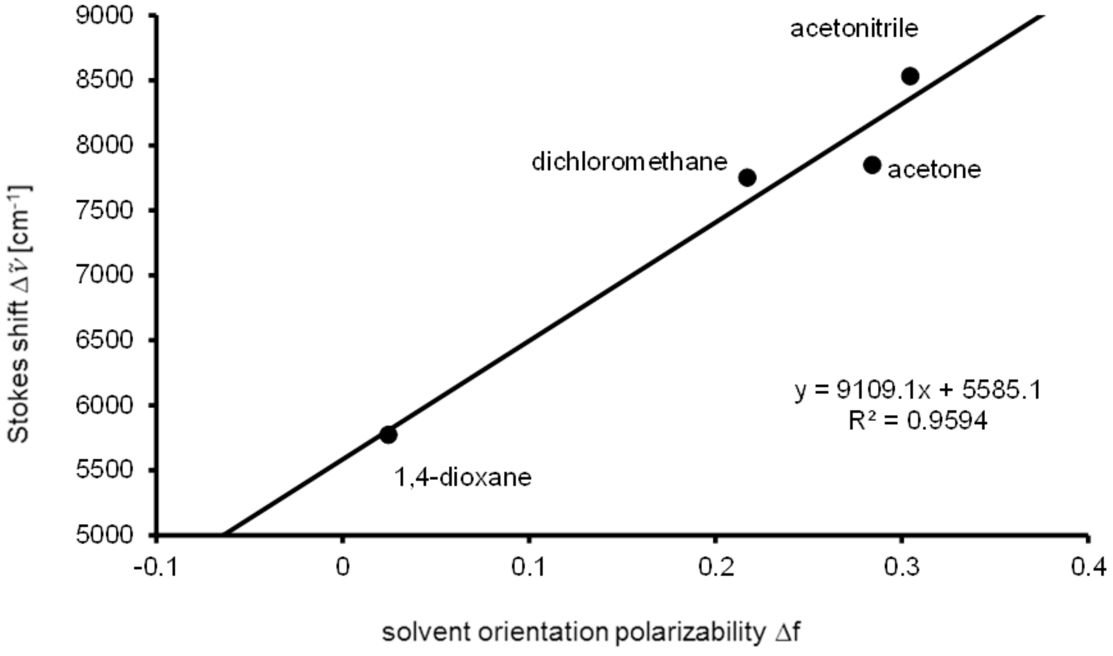

Figure S43. Lippert-Mataga-plot of dyad **5a** (Stokes shifts $\Delta\tilde{\nu}$ (= $\tilde{\nu}_a$ − $\tilde{\nu}_e$) were determined from absorption and emission spectra at T = 298 K, $\Delta f = \frac{\varepsilon_r - 1}{2\varepsilon_r + 1} - \frac{n^2 - 1}{2n^2 + 1}$).

5. DFT and TDDFT Calculations on the *syn*- and *anti*-Structures 5a, 5g, and 5i

5.1 Structure *anti*-5a

B3LYP 6-311G SCRF (solvent = dichloromethane) optimized S_0 ground state xyz-coordinates**

C	-1.514970	-1.487121	-2.394061
N	-0.221151	0.119214	-0.956352
C	1.063924	0.868975	-0.830911
C	1.079420	1.823446	0.390899
N	1.244453	3.125646	0.069779
C	1.410716	4.265014	1.013641
O	0.627420	-0.623616	-2.932106
O	0.996493	1.401497	1.543106
C	2.662532	4.050149	1.881335
C	1.587910	5.516411	0.141366
C	0.154632	4.412740	1.889409
C	2.283973	-0.035126	-0.697779
C	2.274877	-1.208858	0.056065
C	3.419244	-1.983429	0.212233
C	4.650035	-1.600614	-0.367121
C	4.648097	-0.419153	-1.142011
C	3.490705	0.336799	-1.293037
N	5.808749	-2.343872	-0.173764
C	6.969330	-2.073902	-1.012370
C	5.701889	-3.687542	0.380084
C	-1.429552	0.742766	-0.324945
C	-2.602996	-0.146368	0.054152
C	-2.457892	-1.064024	1.122487
C	-3.567909	-1.901586	1.508432
C	-4.790365	-1.765997	0.848729
C	-4.968878	-0.829758	-0.169997
C	-3.859040	0.001632	-0.583639
C	-6.238721	-0.690449	-0.814122
C	-6.427373	0.213670	-1.820057
C	-5.342473	1.031764	-2.240828
C	-4.112025	0.928979	-1.649663

C	-1.241434	-1.198145	1.866886
C	-1.129196	-2.103041	2.889207
C	-2.218222	-2.947171	3.243216
C	-3.404327	-2.842829	2.572179
H	-1.835312	-2.043545	-1.514878
H	-2.358225	-0.885747	-2.738679
H	-1.241493	-2.179039	-3.189299
H	1.196907	1.462647	-1.740602
H	1.293217	3.349770	-0.912846
H	2.799811	4.905546	2.547982
H	2.567453	3.149617	2.488054
H	3.553424	3.957518	1.254712
H	1.719186	6.395244	0.775466
H	0.710834	5.683988	-0.490754
H	2.469688	5.427928	-0.500062
H	-0.001102	3.525123	2.502474
H	0.266351	5.276254	2.550364
H	-0.731079	4.570345	1.268151
H	1.354981	-1.543874	0.518797
H	3.347472	-2.892154	0.793527
H	5.548347	-0.087087	-1.639937
H	3.534611	1.230806	-1.908089
H	7.791694	-2.712304	-0.692348
H	6.777290	-2.264330	-2.078752
H	7.297565	-1.037597	-0.904255
H	5.235250	-3.668066	1.367542
H	5.121150	-4.366882	-0.261338
H	6.702767	-4.100062	0.499486
H	-1.079904	1.199028	0.596374
H	-1.765437	1.561729	-0.960458
H	-5.625114	-2.394701	1.142510
H	-7.053326	-1.325023	-0.481363
H	-7.394112	0.311201	-2.300337
H	-5.493028	1.746469	-3.042151
H	-3.320354	1.570831	-2.010287

H	-0.400680	-0.557622	1.638535
H	-0.199107	-2.173889	3.442235
H	-2.105129	-3.661059	4.051201

Zero-point correction = 0.595967 (Hartree/particle)

Sum of electronic and zero-point energies = -1516.926684 a.u., -951871.49421 kcal/mol

Sum of electronic and thermal energies = -1516.892196 a.u., -951849.85299 kcal/mol

Sum of electronic and thermal enthalpies = -1516.891252 a.u., -951849.2606299999 kcal/mol

Sum of electronic and thermal free energies = -1516.994622 a.u., -951914.125305 kcal/mol

5.2 Structure *syn*-5a

B3LYP 6-311G SCRF (solvent = dichloromethane) optimized S_0 ground state xyz-coordinates**

C	-1.222807	-2.428771	-2.528425
C	-0.966391	-3.926751	-2.555851
N	-0.897852	-1.713119	-1.397286
C	-1.373477	-0.313397	-1.364911
C	-2.670090	-0.180907	-0.520260
N	-3.137806	1.082990	-0.415306
C	-4.428551	1.513854	0.184433
O	-1.716133	-1.889027	-3.522526
O	-3.251950	-1.169399	-0.077210
C	-4.483574	3.040130	0.022031
C	-5.602843	0.866491	-0.569830
C	-4.471413	1.147997	1.678040
C	-0.294942	0.740514	-1.147755
C	-0.044965	1.381891	0.069067
C	0.947597	2.346163	0.201044
C	1.748266	2.730557	-0.898538
C	1.502613	2.072649	-2.126067
C	0.502818	1.115629	-2.235891
N	2.712307	3.718475	-0.785606

C	3.675193	3.902649	-1.864079
C	3.092484	4.197808	0.536918
C	-0.466069	-2.408229	-0.160711
C	0.651490	-1.781923	0.664334
C	1.969708	-1.737885	0.149830
C	3.052611	-1.261746	0.976008
C	2.790435	-0.863563	2.287764
C	1.502441	-0.912748	2.819610
C	0.403607	-1.374890	1.998489
C	1.259012	-0.511145	4.171095
C	0.002165	-0.545592	4.704130
C	-1.088974	-0.980466	3.901436
C	-0.897867	-1.377479	2.605206
C	2.301041	-2.161814	-1.176788
C	3.587683	-2.123549	-1.644463
C	4.647155	-1.648622	-0.823617
C	4.381341	-1.228273	0.449621
H	0.083014	-4.179684	-2.389406
H	-1.559653	-4.444794	-1.798570
H	-1.258806	-4.286818	-3.539886
H	-1.734639	-0.164514	-2.384846
H	-2.559895	1.811098	-0.810228
H	-5.415888	3.425351	0.439716
H	-3.652871	3.522014	0.545877
H	-4.442543	3.325153	-1.033395
H	-6.550275	1.211106	-0.146755
H	-5.563293	-0.220155	-0.492565
H	-5.579009	1.143049	-1.627250
H	-3.639261	1.614084	2.212044
H	-5.405464	1.506156	2.119369
H	-4.414134	0.068402	1.816295
H	-0.629319	1.131424	0.946110
H	1.096322	2.798775	1.171352
H	2.087305	2.311111	-3.003482
H	0.343381	0.643242	-3.200199

H	4.301729	3.014274	-2.027255
H	3.171361	4.142927	-2.803530
H	4.325349	4.740476	-1.617278
H	2.229529	4.606454	1.068367
H	3.538961	3.410303	1.160531
H	3.819365	5.000892	0.425587
H	-1.346135	-2.567489	0.454905
H	-0.120667	-3.395035	-0.456800
H	3.608830	-0.516550	2.910994
H	2.102758	-0.175767	4.765132
H	-0.170601	-0.239889	5.729735
H	-2.088290	-0.992812	4.322106
H	-1.762408	-1.676564	2.029912
H	1.518993	-2.506500	-1.835940
H	3.802395	-2.453413	-2.654820
H	5.659034	-1.622976	-1.211726

Zero-point correction = 0.596034 (Hartree/particle)
Sum of electronic and zero-point energies = -1516.934380 a.u., -951876.32345 kcal/mol
Sum of electronic and thermal energies = -1516.899808 a.u., -951854.62952 kcal/mol
Sum of electronic and thermal enthalpies = -1516.898864 a.u., -951854.03716 kcal/mol
Sum of electronic and thermal free energies = -1517.002577 a.u., -951919.1170675 kcal/mol

5.3 Structure *anti*-5g

B3LYP 6-311G SCRF (solvent = dichloromethane) optimized S_0 ground state xyz-coordinates**

C	0.483071	0.420044	2.870917
C	1.385577	-0.558297	3.598236
N	0.281529	0.222280	1.526619
C	-0.784651	1.022533	0.866553
C	-0.214800	1.898503	-0.266834
N	-0.489497	3.217832	-0.133940
C	-0.136728	4.304515	-1.086036
O	-0.062612	1.337573	3.489215
O	0.413181	1.416025	-1.206841

C	1.391952	4.410617	-1.223199
C	-0.791357	4.041972	-2.453307
C	-0.697063	5.599792	-0.480583
C	-1.966514	0.170719	0.416954
C	-2.921041	-0.206326	1.371419
C	-4.015915	-0.995418	1.047222
C	-4.224422	-1.444481	-0.278875
C	-3.254529	-1.072249	-1.236503
C	-2.161188	-0.283086	-0.891628
N	-5.334987	-2.196159	-0.622738
C	-6.170417	-2.761545	0.429126
C	-5.388200	-2.832432	-1.932658
C	0.893600	-0.897945	0.804158
C	2.351937	-0.727097	0.398599
C	3.020295	-1.824471	-0.238529
C	4.394952	-1.677675	-0.620055
C	5.063655	-0.451343	-0.364349
C	4.398720	0.584674	0.240881
C	3.043628	0.441422	0.619764
C	2.379091	-3.063871	-0.518288
C	3.053943	-4.095205	-1.131768
C	4.409772	-3.946374	-1.501411
C	5.061310	-2.762329	-1.249889
H	2.328786	-0.732445	3.080850
H	1.583195	-0.158909	4.590823
H	0.877666	-1.521911	3.704994
H	-1.137410	1.675600	1.661480
H	-0.992795	3.498226	0.694536
H	1.646727	5.235288	-1.894315
H	1.851806	4.605900	-0.250761
H	1.810129	3.489711	-1.629239
H	-0.562423	4.864513	-3.136094
H	-1.877700	3.973033	-2.352029
H	-0.421331	3.114925	-2.891342
H	-0.253566	5.800525	0.499198

H	-0.468939	6.444298	-1.133797
H	-1.783708	5.543438	-0.367124
H	-2.807123	0.122259	2.399810
H	-4.715002	-1.253993	1.830256
H	-3.348226	-1.395167	-2.263967
H	-1.448367	-0.019886	-1.663321
H	-6.575476	-1.977201	1.073045
H	-7.013131	-3.277017	-0.028655
H	-5.627468	-3.479148	1.060825
H	-4.586455	-3.570787	-2.076853
H	-6.345291	-3.340265	-2.039763
H	-5.317411	-2.090766	-2.732045
H	0.789478	-1.807237	1.402534
H	0.295700	-1.059574	-0.092895
H	6.103358	-0.348954	-0.656590
H	4.906791	1.522933	0.433644
H	2.542855	1.277588	1.091372
H	1.339753	-3.207153	-0.251568
H	2.541470	-5.028924	-1.334712
H	4.930650	-4.765707	-1.983817

Zero-point correction = 0.549461 (Hartree/particle)
Sum of electronic and zero-point energies = -1363.320785 a.u., -855483.7925875 kcal/mol
Sum of electronic and thermal energies = -1363.288779 a.u., -855463.7088225 kcal/mol
Sum of electronic and thermal enthalpies = -1363.287835 a.u., -855463.1164625001 kcal/mol
Sum of electronic and thermal free energies = -1363.387155 a.u., -855525.4397625 kcal/mol

5.4 Structure *syn*-5g

B3LYP 6-311G SCRF (solvent = dichloromethane) optimized S_0 ground state xyz-coordinates**

C	-1.649528	-0.722843	2.003174
C	-1.813517	-1.862752	2.994736
N	-1.168718	-1.000311	0.748762
C	-1.099605	0.081801	-0.268668
C	-2.441695	0.854418	-0.456186

N	-3.556893	0.089573	-0.384533
C	-4.959243	0.543183	-0.577620
O	-1.973408	0.420930	2.321209
O	-2.441362	2.037844	-0.775230
C	-5.151677	1.058584	-2.014241
C	-5.841586	-0.692839	-0.348476
C	-5.317403	1.635155	0.445811
C	0.108671	0.997626	-0.169369
C	1.020540	1.034143	-1.226919
C	2.139038	1.861594	-1.215895
C	2.410310	2.698813	-0.112666
C	1.474906	2.675612	0.947489
C	0.360084	1.847038	0.914671
N	3.548237	3.494732	-0.062735
C	4.322049	3.696811	-1.280633
C	3.636967	4.540595	0.948402
C	-0.847917	-2.377045	0.298495
C	0.432496	-2.531401	-0.502982
C	1.729510	-2.427484	0.097794
C	2.889226	-2.647282	-0.716354
C	2.730526	-2.957830	-2.093337
C	1.478817	-3.058723	-2.645267
C	0.334445	-2.848433	-1.842153
C	1.927655	-2.124797	1.471409
C	3.190723	-2.049751	2.012122
C	4.330567	-2.268398	1.205921
C	4.179079	-2.559003	-0.128723
H	-2.218571	-1.439708	3.911518
H	-2.501364	-2.625903	2.622059
H	-0.865531	-2.353179	3.227484
H	-0.989966	-0.449204	-1.217878
H	-3.438330	-0.856418	-0.056244
H	-4.510869	1.918813	-2.206872
H	-4.913085	0.274921	-2.738506
H	-6.192258	1.358652	-2.164746

H	-6.892915	-0.430567	-0.482449
H	-5.718373	-1.081946	0.666804
H	-5.597771	-1.487657	-1.059677
H	-6.360119	1.937324	0.314721
H	-5.193100	1.262691	1.465730
H	-4.682830	2.512022	0.317449
H	0.860182	0.401713	-2.094652
H	2.800331	1.845197	-2.070911
H	1.610140	3.315431	1.808614
H	-0.334338	1.870336	1.742930
H	3.748000	4.205105	-2.069533
H	5.195923	4.303503	-1.047031
H	4.680376	2.744790	-1.678879
H	4.607508	5.028185	0.866756
H	2.854397	5.305746	0.838731
H	3.564661	4.120165	1.953961
H	-0.801986	-3.016061	1.176697
H	-1.673437	-2.750514	-0.317076
H	3.616146	-3.119374	-2.698779
H	1.358012	-3.301863	-3.694645
H	-0.647307	-2.943853	-2.294578
H	1.073362	-1.938363	2.107033
H	3.313754	-1.816197	3.063604
H	5.320529	-2.204351	1.642947

Zero-point correction = 0.549374 (Hartree/particle)

Sum of electronic and zero-point energies = -1363.314876 a.u., -855480.08469 kcal/mol

Sum of electronic and thermal energies = -1363.283078 a.u., -855460.131445 kcal/mol

Sum of electronic and thermal enthalpies = -1363.282134 a.u., -855459.539085 kcal/mol

Sum of electronic and thermal free energies = -1363.379721 a.u., -855520.7749275 kcal/mol

5.5 Structure *anti*-5i

B3LYP 6-311G SCRF (solvent = dichloromethane) optimized S_0 ground state xyz-coordinates**

C	-0.024493	-2.420930	0.261508

H	0.141161	-2.264577	1.326367
H	0.011683	-3.493988	0.082086
N	1.123111	-1.840234	-0.465938
C	1.408395	-0.422078	-0.164135
H	0.800923	-0.223430	0.722189
C	2.852628	-0.126905	0.241120
C	5.521874	0.393678	1.125814
C	3.656584	0.819991	-0.394209
C	3.408345	-0.810960	1.328551
C	4.705852	-0.573702	1.760352
C	4.958416	1.076616	0.025006
H	3.281217	1.369729	-1.249775
H	2.822769	-1.563620	1.847053
H	5.081747	-1.143219	2.598720
H	5.535242	1.812774	-0.516880
C	1.805000	-2.646321	-1.346733
C	3.030099	-2.113315	-2.058832
H	3.243877	-2.790746	-2.884444
H	3.883831	-2.111105	-1.377262
H	2.884073	-1.105773	-2.438335
O	1.447901	-3.812342	-1.541520
C	0.875096	0.531806	-1.270705
N	0.369792	1.690457	-0.789317
H	0.361372	1.805825	0.213579
C	-0.172187	2.828545	-1.580699
C	-0.599660	3.891571	-0.558326
H	0.251822	4.226800	0.041280
H	-1.370231	3.503302	0.113992
H	-1.010655	4.760323	-1.076146
C	-1.392426	2.368060	-2.396269
H	-2.165786	1.966075	-1.737075
H	-1.112611	1.597075	-3.113903
H	-1.812458	3.217107	-2.942256
C	0.917680	3.400738	-2.503142
H	1.776291	3.742967	-1.919295

H	0.519873	4.254904	-3.057382
H	1.256036	2.651907	-3.219590
O	0.932296	0.244582	-2.461827
N	6.803583	0.665449	1.572201
C	7.685813	1.484532	0.750835
H	7.901973	1.025521	-0.224482
H	8.627130	1.628040	1.278683
H	7.252672	2.472320	0.575332
H	-1.055150	-2.605108	-2.175532
C	-1.747556	-2.094268	-1.516423
C	-1.376325	-1.886026	-0.182763
C	-3.883358	-1.000541	-1.185641
C	-2.273326	-1.224276	0.688421
C	-2.968074	-1.666118	-2.013586
C	-3.537742	-0.775351	0.178913
C	-1.992401	-0.970167	2.079594
H	-3.223720	-1.845570	-3.052380
C	-2.875212	-0.325198	2.887852
H	-1.055478	-1.302720	2.505505
H	-2.628807	-0.152483	3.930330
C	-4.142036	0.137376	2.399993
C	-5.063925	0.804188	3.220873
C	-4.463840	-0.098010	1.030778
C	-6.285414	1.236758	2.710909
H	-4.815198	0.979617	4.262058
H	-6.987476	1.750587	3.357848
C	-6.612098	1.012681	1.376369
H	-7.565905	1.351229	0.985974
C	-5.720518	0.348820	0.518644
C	-6.032313	0.102567	-0.861045
H	-6.988450	0.442940	-1.244283
C	-5.153925	-0.542632	-1.673271
H	-5.399858	-0.724155	-2.714258
C	7.429348	-0.229291	2.537906
H	6.844437	-0.283791	3.459320

H 8.411801 0.162330 2.796288

Zero-point correction = 0.608416 (Hartree/particle)
Sum of electronic and zero-point energies = -1593.185127 a.u., -999723.6671925 kcal/mol
Sum of electronic and thermal energies = -1593.150525 a.u., -999701.9544375 kcal/mol
Sum of electronic and thermal enthalpies = -1593.149580 a.u., -999701.36145 kcal/mol
Sum of electronic and thermal free energies = -1593.253836 a.u., -999766.782090 kcal/mol

5.6 Structure *syn*-5i

B3LYP 6-311G SCRF (solvent = dichloromethane) optimized S_0 ground state xyz-coordinates**

C -0.385558 -1.762974 0.369176
H -0.682702 -1.401442 -0.615091
H -0.250876 -2.842751 0.280229
N -1.508426 -1.536109 1.282468
C -2.539093 -0.557205 0.902715
H -3.179362 -0.428705 1.774475
C -1.983725 0.822750 0.579628
C -0.902914 3.423161 0.075329
C -1.530990 1.191068 -0.692312
C -1.888837 1.785343 1.591063
C -1.362859 3.049271 1.360176
C -0.997193 2.447759 -0.945522
H -1.594950 0.491274 -1.518870
H -2.228438 1.542870 2.593554
H -1.311884 3.745829 2.185132
H -0.655683 2.668834 -1.946836
C -1.687523 -2.445552 2.293441
C -2.932739 -2.344876 3.156507
H -2.881364 -3.136736 3.900798
H -3.842998 -2.474123 2.566515
H -2.994925 -1.383484 3.672492
O -0.850635 -3.322893 2.510205
C -3.479270 -1.152344 -0.195985
N -4.428233 -0.294490 -0.630647

H	-4.358741	0.657593	-0.299897
C	-5.509167	-0.577553	-1.612849
C	-6.311724	0.724480	-1.749940
H	-5.681450	1.539050	-2.118955
H	-6.742686	1.023927	-0.790121
H	-7.129965	0.583952	-2.458911
C	-6.419548	-1.698468	-1.083178
H	-6.858162	-1.415093	-0.122637
H	-5.861240	-2.625535	-0.952645
H	-7.233077	-1.879699	-1.790577
C	-4.902046	-0.963176	-2.973023
H	-4.265994	-0.158772	-3.351905
H	-5.700517	-1.138465	-3.698895
H	-4.305006	-1.871464	-2.889280
O	-3.376705	-2.320661	-0.555775
N	-0.403271	4.686152	-0.175744
C	0.262148	4.955888	-1.444501
H	1.163626	4.343674	-1.585045
H	0.551499	6.004829	-1.475139
H	-0.410068	4.774626	-2.287081
H	0.229227	-0.361640	2.638272
C	1.083542	-0.449468	1.979751
C	0.938412	-1.128664	0.771845
C	3.419470	0.034605	1.531071
C	2.055109	-1.245648	-0.089922
C	2.293631	0.122463	2.357985
C	3.302070	-0.658599	0.289602
C	1.995319	-1.940160	-1.349631
H	2.370548	0.644002	3.306265
C	3.079952	-2.039059	-2.165572
H	1.066320	-2.401862	-1.658861
H	3.004233	-2.570532	-3.108489
C	4.340964	-1.455757	-1.809929
C	5.471251	-1.547492	-2.638035
C	4.439805	-0.762468	-0.567335

C	6.678882	-0.970156	-2.256634
H	5.393895	-2.076406	-3.581959
H	7.542808	-1.050031	-2.906897
C	6.787021	-0.291261	-1.044762
H	7.732372	0.154745	-0.754447
C	5.684366	-0.174913	-0.184811
C	5.765217	0.516321	1.072765
H	6.713454	0.959233	1.358649
C	4.683934	0.614917	1.890361
H	4.758513	1.137809	2.838221
C	-0.118255	5.580234	0.939610
H	-1.018255	5.772097	1.529351
H	0.226922	6.535145	0.547194

Zero-point correction = 0.608554 (Hartree/particle)

Sum of electronic and zero-point energies = -1593.190946 a.u., -999727.318615 kcal/mol

Sum of electronic and thermal energies = -1593.155632 a.u., -999705.15908 kcal/mol

Sum of electronic and thermal enthalpies = -1593.154688 a.u., -999704.56672 kcal/mol

Sum of electronic and thermal free energies = -1593.260765 a.u., -999771.1300375 kcal/mol

5.7 TDDFT Calculations (Absorption Bands) of *syn*- and *anti*-Structures 5a, 5g, and 5i

TDDFT calculations of the absorption maxima for the *syn*- and *anti*-dyads **5a**, **5g**, and **5i** (starting geometries see chapters 4.1.-4.6.) were calculated using the PBE1PBE[3]/6-31G**[4] functional and basis set applying the Polarizable Continuum Model (PCM)[5] for dichloromethane as a solvent. The data, also comparison to the experimental UV/Vis absorptions are summarized in Table S4.

Table S4. Selected UV/Vis absorption bands and TDDFT calculations of the absorption maxima for the *syn*- and *anti*-dyads **5a**, **5g**, and **5i** (calculated using the PBE1PBE/6-31G** functional and basis set applying the Polarizable Continuum Model (PCM) for dichloromethane as a solvent).

	$\lambda_{max,abs}$ [nm] (ε [L mol^{-1} cm^{-1}])[a]	$\lambda_{max,calcd}$ [nm] *syn*-**5**	Most dominant contributions	Oscillator strength	$\lambda_{max,calcd}$ [nm] *anti*-**5**	Most dominant contributions	Oscillator strength
**5a**	391 (7700)	433.1	HOMO → LUMO (98.9%)	0.0167	418.3	HOMO → LUMO (99.7%)	0.0030
	371 (8300)	388.1	HOMO-1 → LUMO (97.9%)	0.0850	393.4	HOMO-1 → LUMO (98.7%)	0.1362
	352 (5100)	322.7	HOMO-3 → LUMO (37.6%)	0.0002	321.0	HOMO-1 → LUMO+1 (47.3%)<br>HOMO-2 → LUMO (38.7%)	0.0005
		297.5	HOMO-2 → LUMO (84.4%)<br>HOMO → LUMO+1 (7.0%)	0.0037	290.5	HOMO-4 → LUMO (61.7%)<br>HOMO-2 → LUMO (32.8%)	0.0056
		294.3	HOMO → LUMO+1 (58.6%)<br>HOMO-1 → LUMO+1 (20.8%)	0.0159	287.9	HOMO → LUMO+1 (98.4%)	0.0007
		283.0	HOMO → LUMO+2 (57.6%)<br>HOMO → LUMO+3 (11.3%)	0.0406	286.3	HOMO → LUMO+2 (48.6%)<br>HOMO → LUMO+5 (21.6%)	0.0718
		280.7	HOMO-4 → LUMO (78.7%)<br>HOMO-3 → LUMO (7.8%)	0.0024	275.7	HOMO → LUMO+2 (43.1%)<br>HOMO → LUMO+5 (41.9%)	0.3140
		271.7	HOMO → LUMO+3 (65.6%)<br>HOMO → LUMO+2 (15.5%)	0.0713	273.8	HOMO-3 → LUMO (93.5%)<br>HOMO-2 → LUMO (2.4%)	0.0021
		270.2	HOMO → LUMO+4 (31.2%)<br>HOMO-6 → LUMO (17.8%)	0.0133	269.1	HOMO → LUMO+4 (43.5%)<br>HOMO → LUMO+6 (23.5%)	0.0091
		264.1	HOMO-5 → LUMO (48.1%)<br>HOMO → LUMO+4 (31.3%)	0.0049	268.4	HOMO-7 → LUMO (47.5%)<br>HOMO-1 → LUMO+3 (25.0%)	0.0043

**5g**	353 (200)	366.1	HOMO → LUMO (99.7%)	0.0023	321.3	HOMO → LUMO (99.2%)	0.0099
	316 (sh)	296.3	HOMO → LUMO+1 (99.0%)	0.0094	288.6	HOMO-1 → LUMO (95.3%)<br>HOMO-3 → LUMO+1 (3.1%)	0.1577
	309 (sh)	287.4	HOMO-1 → LUMO (95.1%)<br>HOMO-4 → LUMO+1 (3.1%)	0.1155	284.4	HOMO → LUMO+2 (86.3%)<br>HOMO → LUMO+3 (5.3%)<br>HOMO-4 → LUMO+3 (2.6%)	0.0532
		277.7	HOMO-1 → LUMO+1 (45.7%)<br>HOMO-4 → LUMO (41.8%)<br>HOMO-2 → LUMO (8.8%)	0.0002	277.7	HOMO-1 → LUMO+1 (49.4%)<br>HOMO-3 → LUMO (44.1%)<br>HOMO → LUMO+1 (3.3%)	0.0002
	295 (sh)	277.7	HOMO → LUMO+3 (79.3%)<br>HOMO → LUMO+2 (12.4%)	0.0380	267.4	HOMO → LUMO+1 (62.8%)<br>HOMO → LUMO+3 (28.4%)<br>HOMO → LUMO+5 (2.6%)	0.1180
	283 (sh)	268.2	HOMO → LUMO+4 (42.7%)<br>HOMO → LUMO+2 (38.6%)<br>HOMO → LUMO+5 (6.1%)<br>HOMO → LUMO+3 (4.0%)<br>HOMO → LUMO+8 (3.4%)	0.0568	264.7	HOMO → LUMO+3 (38.3%)<br>HOMO → LUMO+1 (30.6%)<br>HOMO → LUMO+4 (15.6%)<br>HOMO → LUMO+5 (7.5%)<br>HOMO → LUMO+2 (3.2%)	0.0535
		260.4	HOMO-2 → LUMO (84.3%)<br>HOMO-4 → LUMO (5.3%)<br>HOMO-3 → LUMO (3.7%)<br>HOMO-1 → LUMO+1 (2.6%)<br>HOMO-7 → LUMO (2.1%)	0.0037	256.5	HOMO → LUMO+4 (59.7%)<br>HOMO → LUMO+3 (20.1%)<br>HOMO → LUMO+5 (6.0%)<br>HOMO → LUMO+2 (4.8%)<br>HOMO → LUMO+6 (3.2%)	0.3341
	270 (24000)	255.9	HOMO → LUMO+4 (40.7%)<br>HOMO → LUMO+5 (29.1%)<br>HOMO → LUMO+2 (19.1%)<br>HOMO → LUMO+3 (3.4%)<br>HOMO-3 → LUMO (3.1%)	0.1987	254.4	HOMO-2 → LUMO (95.6%)	0.0085
		254.0	HOMO-3 → LUMO (89.8%)<br>HOMO-2 → LUMO (3.5%)	0.0126	241.4	HOMO → LUMO+7 (72.0%)<br>HOMO → LUMO+6 (15.4%)<br>HOMO → LUMO+5 (3.0%)<br>HOMO → LUMO+4 (2.2%)	0.0035

						HOMO → LUMO+8 (2.1%)	
		248.6	HOMO → LUMO+5 (53.4%)<br>HOMO → LUMO+2 (25.5%)<br>HOMO → LUMO+3 (5.4%)<br>HOMO → LUMO+6 (5.3%)<br>HOMO → LUMO+4 (3.9%)<br>HOMO → LUMO+8 (2.7%)	0.0670	240.4	HOMO → LUMO+5 (64.9%)<br>HOMO → LUMO+4 (12.1%)<br>HOMO → LUMO+9 (6.0%)<br>HOMO → LUMO+7 (5.2%)<br>HOMO → LUMO+8 (3.4%)<br>HOMO → LUMO+3 (2.8%)	0.0097
**5i**	377 (800)	365.3	HOMO → LUMO (94.7%)<br>HOMO-1 → LUMO (4.6%)	0.0435	363.2	HOMO → LUMO (98.8%)	0.0511
	347 (36000)	346.9	HOMO-1 → LUMO (90.8%)<br>HOMO-2 → LUMO+1 (4.7%)<br>HOMO → LUMO (3.9%)	0.4684	344.6	HOMO-1 → LUMO (93.8%)<br>HOMO-2 → LUMO+1 (4.7%)	0.4882
	331 (27000)	329.0	HOMO-1 → LUMO+1 (42.6%)<br>HOMO-2 → LUMO (41.7%)<br>HOMO → LUMO+1 (14.6%)	0.0017	330.3	HOMO-1 → LUMO+1 (53.6%)<br>HOMO-2 → LUMO (40.0%)<br>HOMO → LUMO+1 (3.6%)	0.0016
	317 (13000)	292.7	HOMO → LUMO+1 (72.1%)<br>HOMO-1 → LUMO+1 (15.8%)<br>HOMO → LUMO+2 (4.5%)<br>HOMO → LUMO+3 (4.0%)	0.0017	292.5	HOMO → LUMO+1 (93.9%)<br>HOMO-1 → LUMO+1 (2.3%)	0.0222
	300 (sh)	286.6	HOMO-1 → LUMO+2 (50.3%)<br>HOMO → LUMO+2 (20.5%)<br>HOMO-1 → LUMO+3 (19.4%)<br>HOMO → LUMO+3 (4.9%)	0.0079	288.5	HOMO-1 → LUMO+2 (85.2%)<br>HOMO → LUMO+2 (7.7%)<br>HOMO-1 → LUMO+3 (3.3%)	0.0046
		283.3	HOMO → LUMO+3 (49.3%)<br>HOMO → LUMO+2 (23.6%)<br>HOMO → LUMO+1 (7.2%)<br>HOMO-1 → LUMO+2 (5.7%)<br>HOMO-1 → LUMO+3 (5.6%)<br>HOMO-1 → LUMO+1 (3.4%)	0.0275	286.0	HOMO → LUMO+3 (80.6%)<br>HOMO → LUMO+4 (5.2%)<br>HOMO → LUMO+2 (5.1%)	0.0451
	279 (47600)	273.5	HOMO-2 → LUMO (51.4%)<br>HOMO-1 → LUMO+1 (33.7%)<br>HOMO → LUMO+1 (4.6%)	0.3257	278.9	HOMO-3 → LUMO (40.7%)<br>HOMO-2 → LUMO (37.8%)	0.1554

		HOMO-1 → LUMO+2 (3.1%)			HOMO-1 → LUMO+1 (17.2%)	
268 (39900)	265.1	HOMO → LUMO+4 (65.2%)<br>HOMO-3 → LUMO (11.3%)<br>HOMO-1 → LUMO+4 (7.2%)<br>HOMO → LUMO+5 (3.7%)<br>HOMO → LUMO+6 (2.8%)<br>HOMO → LUMO+9 (2.3%)	0.1141	271.7	HOMO-3 → LUMO (53.5%)<br>HOMO-1 → LUMO+1 (24.1%)<br>HOMO-2 → LUMO (17.9%)	0.2748
258 (sh)	264.8	HOMO-3 → LUMO (84.2%)<br>HOMO → LUMO+4 (8.0%)	0.0346	267.7	HOMO → LUMO+4 (47.2%)<br>HOMO → LUMO+2 (35.4%)<br>HOMO → LUMO+7 (5.2%)<br>HOMO → LUMO+6 (4.4%)<br>HOMO-1 → LUMO+2 (2.5%)	0.1567
	264.0	HOMO-5 → LUMO (95.7%)	0.0072	262.8	HOMO-6 → LUMO (88.2%)<br>HOMO-7 → LUMO (4.3%)<br>HOMO-5 → LUMO (2.2%)	0.0009

[a] Recorded in dichloromethane, T = 293 K, c(**5**) = 10^{-4} M.

5.8 TDDFT Calculations (Absorption and Exciplex Emission Bands) of *syn*- and *anti*-Structures 5a

5.8.1 *anti*-Structure 5a

PBE1PBE 6-31G SCRF (solvent = dichloromethane) optimized S_0 ground state xyz-coordinates**

C	-0.407284	-0.458895	-2.192047
C	-1.686554	-1.186476	-2.517543
N	-0.292845	0.222310	-0.993057
C	1.007692	0.907309	-0.870549
C	1.086667	1.832130	0.355270
N	1.394552	3.108614	0.061788
C	1.659394	4.180945	1.038755
O	0.534878	-0.499884	-2.975899
O	0.937821	1.405345	1.501372
C	2.848589	3.795930	1.921669
C	1.994824	5.429285	0.224847
C	0.410895	4.431121	1.888081
C	2.186986	-0.042895	-0.749050
C	2.102485	-1.272536	-0.099206
C	3.215547	-2.087353	0.059090
C	4.485095	-1.690893	-0.410614
C	4.561445	-0.451442	-1.079443
C	3.435703	0.345748	-1.234017
N	5.604019	-2.478022	-0.220025
C	6.821364	-2.150179	-0.926644
C	5.436186	-3.835148	0.248035
C	-1.464244	0.836236	-0.325785
C	-2.601896	-0.076504	0.083769
C	-2.376645	-1.049839	1.083551
C	-3.437067	-1.942730	1.470809
C	-4.697333	-1.800379	0.892502
C	-4.957378	-0.801835	-0.044007
C	-3.896030	0.081131	-0.464644
C	-6.264608	-0.654792	-0.598041

C	-6.535129	0.308063	-1.526333
C	-5.498173	1.178949	-1.953265
C	-4.232807	1.069149	-1.445294
C	-1.122429	-1.182661	1.756428
C	-0.928226	-2.150374	2.705227
C	-1.965714	-3.056541	3.051024
C	-3.188753	-2.946937	2.453284
H	-2.005026	-1.842088	-1.705037
H	-2.505745	-0.491954	-2.722009
H	-1.494107	-1.774643	-3.414835
H	1.164646	1.502014	-1.780659
H	1.479020	3.352814	-0.913316
H	3.062723	4.602955	2.629073
H	2.629729	2.885788	2.483315
H	3.740791	3.626321	1.310958
H	2.199904	6.266590	0.896776
H	1.159225	5.713384	-0.424088
H	2.882691	5.268688	-0.396348
H	0.146753	3.535459	2.453427
H	0.597869	5.248488	2.591371
H	-0.435871	4.710820	1.253313
H	1.142384	-1.615995	0.273244
H	3.089795	-3.043500	0.553746
H	5.502670	-0.106706	-1.491290
H	3.538007	1.292326	-1.761114
H	7.598831	-2.862062	-0.646570
H	6.704613	-2.180702	-2.020988
H	7.178764	-1.151369	-0.652775
H	4.953830	-3.855404	1.231415
H	4.837030	-4.454913	-0.436788
H	6.417657	-4.298258	0.357339
H	-1.085028	1.288785	0.590742
H	-1.835790	1.659706	-0.940556

H	-5.498821	-2.473805	1.187851
H	-7.042840	-1.333551	-0.258753
H	-7.533393	0.411742	-1.940488
H	-5.713297	1.944093	-2.693449
H	-3.477595	1.758881	-1.803872
H	-0.320388	-0.483897	1.540343
H	0.031763	-2.220459	3.208972
H	-1.786517	-3.821808	3.800467

Electronic energy: –1515.415992 a.u. = –41236.5907247088 eV (set to 0.00 eV)

TDDFT-calculated Franck-Condon longest wavelength absorption $S_0 \rightarrow S_1$*: 2.9878 eV (414.97 nm)

PBE1PBE 6-31G SCRF (solvent = dichloromethane) vibrationally excited S_0 state xyz-coordinates**

C	-0.199047	-0.790680	-1.919151
C	-1.430554	-1.579931	-2.255815
N	-0.199680	0.144968	-0.924061
C	1.054152	0.899076	-0.886739
C	1.129912	1.846894	0.335460
N	1.069784	3.151454	0.023027
C	1.128571	4.270109	0.985041
O	0.850359	-1.007514	-2.540701
O	1.276702	1.397570	1.468335
C	2.467177	4.242536	1.725825
C	1.014559	5.550484	0.160098
C	-0.040875	4.172221	1.967062
C	2.253563	-0.006865	-0.803317
C	2.266091	-1.119688	0.068215
C	3.398244	-1.867970	0.240033
C	4.602260	-1.530150	-0.449222
C	4.591044	-0.391472	-1.311023
C	3.446510	0.343116	-1.470462
N	5.719787	-2.263214	-0.289866

C	6.925072	-1.970857	-1.054186
C	5.758897	-3.375942	0.649453
C	-1.400533	0.755330	-0.280321
C	-2.544770	-0.145557	0.063779
C	-2.391670	-1.038770	1.167099
C	-3.494538	-1.893246	1.540624
C	-4.703777	-1.796770	0.829341
C	-4.882780	-0.895547	-0.237133
C	-3.781248	-0.051042	-0.644630
C	-6.115963	-0.800271	-0.927958
C	-6.294703	0.066655	-1.995155
C	-5.228387	0.868893	-2.413150
C	-4.003416	0.803028	-1.754981
C	-1.201476	-1.133919	1.929949
C	-1.077160	-2.023287	2.995283
C	-2.141788	-2.863281	3.338387
C	-3.327857	-2.793360	2.622667
H	-1.770352	-2.153612	-1.391219
H	-2.258875	-0.933930	-2.556532
H	-1.165322	-2.250883	-3.073250
H	1.150376	1.487635	-1.809712
H	0.919686	3.392507	-0.945207
H	2.521694	5.086431	2.420101
H	2.575083	3.315943	2.292884
H	3.299160	4.323921	1.019341
H	1.055896	6.420081	0.820735
H	0.065304	5.586245	-0.385286
H	1.836920	5.631953	-0.558814
H	0.014126	3.245609	2.541911
H	-0.012140	5.016944	2.661925
H	-0.995537	4.199137	1.432654
H	1.354877	-1.389475	0.588548
H	3.369251	-2.726474	0.897837

H	5.490307	-0.092436	-1.832928
H	3.455762	1.206979	-2.126952
H	7.642300	-2.773501	-0.902155
H	6.697727	-1.906845	-2.119813
H	7.373818	-1.030444	-0.720972
H	5.405424	-3.060314	1.632754
H	5.140763	-4.204108	0.290546
H	6.785428	-3.720791	0.745675
H	-1.024170	1.194777	0.648002
H	-1.729116	1.591886	-0.904759
H	-5.536676	-2.434061	1.120658
H	-6.934025	-1.438288	-0.599390
H	-7.253148	0.115695	-2.505361
H	-5.348117	1.544619	-3.255905
H	-3.198151	1.435337	-2.117563
H	-0.366220	-0.478972	1.702096
H	-0.148887	-2.057701	3.559805
H	-2.045146	-3.562248	4.165417

Electronic energy: –1515.400116 a.u. = –41236.1587165224 eV (equals ΔE = 0.4320081864 eV)

TDDFT-calculated Franck-Condon longest wavelength absorption $S_0* \rightarrow S_1$: 2.1747 eV (570.11 nm)

5.8.2 *syn*-Structure 5a

PBE1PBE 6-31G SCRF (solvent = dichloromethane) optimized S_0 ground state xyz-coordinates**

C	-1.106430	3.030999	1.813590
C	-0.883502	4.466546	1.388992
N	-0.868069	2.028853	0.905673
C	-1.304777	0.692369	1.309120
C	-2.614802	0.305489	0.599266
N	-3.021294	-0.958807	0.823686
C	-4.305289	-1.545418	0.404060
O	-1.490279	2.791292	2.958113

O	-3.265359	1.136244	-0.034907
C	-4.295684	-2.992358	0.894178
C	-5.463853	-0.781595	1.050538
C	-4.425471	-1.514951	-1.121610
C	-0.199939	-0.344947	1.363179
C	0.002000	-1.346067	0.409807
C	1.052170	-2.248775	0.509332
C	1.966250	-2.190520	1.581931
C	1.766124	-1.173982	2.541007
C	0.704813	-0.289980	2.428504
N	3.001074	-3.092822	1.695639
C	4.037522	-2.857042	2.675978
C	3.300331	-3.964641	0.581820
C	-0.560317	2.325218	-0.496479
C	0.488041	1.465676	-1.174682
C	1.841961	1.577613	-0.787072
C	2.862316	0.873031	-1.516288
C	2.502663	0.085726	-2.609756
C	1.172744	-0.043847	-3.003830
C	0.138063	0.649908	-2.274425
C	0.825251	-0.858779	-4.123191
C	-0.474761	-1.009992	-4.509711
C	-1.503347	-0.353015	-3.782880
C	-1.211034	0.446404	-2.711576
C	2.262623	2.382534	0.315039
C	3.581877	2.487730	0.662609
C	4.582411	1.790363	-0.064118
C	4.227012	1.003497	-1.122790
H	0.144885	4.663137	1.070656
H	-1.547081	4.748293	0.566111
H	-1.104977	5.092583	2.252463
H	-1.624161	0.856174	2.346671
H	-2.401864	-1.545369	1.364451

H	-5.228838	-3.487313	0.612648
H	-3.466091	-3.552677	0.449605
H	-4.203834	-3.038517	1.984832
H	-6.418488	-1.227027	0.753309
H	-5.452231	0.264366	0.737229
H	-5.389434	-0.823040	2.141738
H	-3.591504	-2.052215	-1.584163
H	-5.359685	-1.994618	-1.430350
H	-4.422161	-0.485458	-1.484487
H	-0.665798	-1.427281	-0.442699
H	1.164981	-2.996544	-0.266725
H	2.441134	-1.072921	3.382646
H	0.579979	0.479319	3.187548
H	4.570777	-1.908386	2.512596
H	3.625377	-2.843833	3.690981
H	4.763318	-3.669628	2.629532
H	2.447409	-4.610712	0.346229
H	3.570951	-3.412379	-0.330834
H	4.135758	-4.612100	0.850264
H	-1.491839	2.305840	-1.061518
H	-0.199575	3.353586	-0.537581
H	3.276605	-0.435859	-3.168668
H	1.626742	-1.361226	-4.658848
H	-0.728340	-1.633960	-5.361538
H	-2.539181	-0.491297	-4.079458
H	-2.030569	0.902518	-2.167899
H	1.518358	2.899958	0.906622
H	3.868397	3.106134	1.508316
H	5.624528	1.884608	0.226393

Electronic energy: −1515.42559 a.u. = −41236.851899726 eV (set to 0.00 eV)

TDDFT-calculated Franck-Condon longest wavelength absorption $S_0 \rightarrow S_1$*: 2.8834 eV (429.99 nm)

PBE1PBE 6-31G SCRF (solvent = dichloromethane) vibrationally excited S_0 state xyz-coordinates**

C	-1.279669	-3.487227	0.219569
C	-1.049507	-4.444577	1.365073
N	-0.965983	-2.166937	0.403899
C	-1.394901	-1.268895	-0.653374
C	-2.698669	-0.535359	-0.284931
N	-3.121889	0.354577	-1.207323
C	-4.418755	1.057838	-1.197909
O	-1.732054	-3.902507	-0.849173
O	-3.323074	-0.833166	0.727646
C	-4.427207	1.955185	-2.434242
C	-5.562461	0.043221	-1.277297
C	-4.542955	1.913737	0.063962
C	-0.235744	-0.471720	-1.203472
C	-0.083968	0.921297	-1.084442
C	1.073206	1.538938	-1.490482
C	2.131214	0.782302	-2.072299
C	1.918700	-0.605037	-2.315631
C	0.769603	-1.203422	-1.872748
N	3.318008	1.359573	-2.349322
C	4.396136	0.599425	-2.963923
C	3.592332	2.720490	-1.918060
C	-0.506041	-1.622552	1.705211
C	0.510671	-0.519514	1.638564
C	1.857951	-0.850485	1.294402
C	2.862849	0.187417	1.277616
C	2.491126	1.500153	1.625008
C	1.178231	1.837460	2.013549
C	0.156445	0.816096	2.017535
C	0.840389	3.157889	2.393494
C	-0.450588	3.505903	2.761507
C	-1.450435	2.528369	2.749423

C	-1.151630	1.219275	2.383390
C	2.270704	-2.157285	0.945481
C	3.588072	-2.451279	0.594365
C	4.553543	-1.442282	0.578379
C	4.191343	-0.142873	0.918420
H	0.006769	-4.507238	1.644642
H	-1.611898	-4.148558	2.254896
H	-1.383715	-5.428664	1.038429
H	-1.703519	-1.962247	-1.451982
H	-2.575080	0.440943	-2.051884
H	-5.369498	2.506614	-2.486644
H	-3.608976	2.682300	-2.398137
H	-4.331717	1.364978	-3.352588
H	-6.525223	0.563734	-1.285080
H	-5.535688	-0.629211	-0.417267
H	-5.486247	-0.552391	-2.192500
H	-3.714911	2.626283	0.130035
H	-5.482036	2.475201	0.037029
H	-4.533722	1.286356	0.956774
H	-0.855757	1.510470	-0.604619
H	1.183258	2.603771	-1.337197
H	2.682737	-1.204473	-2.791984
H	0.637092	-2.272181	-2.006248
H	4.779609	-0.147152	-2.261354
H	4.045868	0.101695	-3.870277
H	5.199109	1.281352	-3.234141
H	2.980950	3.436366	-2.475630
H	3.380576	2.819420	-0.848823
H	4.641061	2.945256	-2.096519
H	-1.389642	-1.309469	2.264323
H	-0.071096	-2.465467	2.247406
H	3.258779	2.272505	1.653783
H	1.629151	3.907857	2.391074

H	-0.681305	4.527707	3.051036
H	-2.470408	2.784792	3.023967
H	-1.962038	0.499100	2.362558
H	1.539303	-2.957806	0.921570
H	3.858254	-3.471142	0.332946
H	5.583134	-1.670611	0.314246

Electronic energy: −1515.41285067 a.u. = −41236.505245eV (equals ΔE = 0.346655 eV)

TDDFT-calculated Franck-Condon longest wavelength absorption $S_0^* \rightarrow S_1$: 2.1698 eV (571.4 nm)

---

[1] Reichardt, C. (2010). Solvents and Solvent Effects in Organic Chemistry. Wiley-VCH Publishers, 4th ed., Weinheim. DOI: 10.1002/9783527632220

[2] Smallwood, I. M. (1996). *Handbook of organic solvent properties*, Arnold, London.

[3] a) Perdew, P., Burke, K., and Ernzerhof, M. (1996). Generalized gradient approximation made simple. Phys. Rev. Lett. 77, 3865-68. DOI: 10.1103/PhysRevLett.77.3865. b) Perdew, J. P., Burke, K., and Ernzerhof, M. (1997). Errata: Generalized gradient approximation made simple. Phys. Rev. Lett. 78, 1396. DOI: 10.1103/PhysRevLett.78.1396 c) Adamo, C., and Barone, V. (1999). Toward reliable density functional methods without adjustable parameters: The PBE0 model. J. Chem. Phys. 110, 6158–6170. DOI: 10.1063/1.478522 d) Ernzerhof, M., and Scuseria, G. E., (1999). Assessment of the Perdew-Burke-Ernzerhof exchange-correlation functional. J. Chem. Phys. 110, 5029-5036. DOI: 10.1063/1.478401

[4] Krishnan, R., Binkley, J. S., Seeger, R., and Pople J. A. (1980). Self-consistent molecular orbital methods. XX. A basis set for correlated wave functions. J. Chem. Phys. 72, 650−654. DOI: 10.1063/1.438955

[5] Scalmani, G., and Frisch, M. J. (2010). Continuous surface charge polarizable continuum models of solvation. I. General formalism. J. Chem. Phys. 132, 114110. DOI: 10.1063/1.3359469
